# A Solid‐State Intramolecular Wittig Reaction Enables Efficient Synthesis of Endofullerenes Including Ne@C_60_, ^3^He@C_60_, and HD@C_60_

**DOI:** 10.1002/anie.202100817

**Published:** 2021-03-04

**Authors:** Gabriela Hoffman, Mark C. Walkey, John Gräsvik, George R. Bacanu, Shamim Alom, Sally Bloodworth, Mark E. Light, Malcolm H. Levitt, Richard J. Whitby

**Affiliations:** ^1^ Chemistry, Faculty of Engineering and Physical Sciences University of Southampton Southampton SO17 1BJ UK; ^2^ Current address: Iggesund Paperboard AB Iggesunds Bruk LSKA 82580 Iggesund Sweden

**Keywords:** endohedral fullerene, NMR spectroscopy, phosphorus ylid, synthetic methods, X-ray diffraction

## Abstract

An open‐cage fullerene incorporating phosphorous ylid and carbonyl group moieties on the rim of the orifice can be filled with gases (H_2_, He, Ne) in the solid state, and the cage opening then contracted in situ by raising the temperature to complete an intramolecular Wittig reaction, trapping the atom or molecule inside. Known transformations complete conversion of the product fullerene to C_60_ containing the endohedral species. As well as providing an improved synthesis of large quantities of ^4^He@C_60_, H_2_@C_60_, and D_2_@C_60_, the method allows the efficient incorporation of expensive gases such as HD and ^3^He, to prepare HD@C_60_ and ^3^He@C_60_. The method also enables the first synthesis of Ne@C_60_ by molecular surgery, and its characterization by crystallography and ^13^C NMR spectroscopy.

## Graphical abstract

Solid‐state filling of a stable phosphorus ylid open‐cage fullerene is followed by heating, inducing an in situ intramolecular Wittig reaction to reduce the size of the cage opening and trapping a single gas atom or molecule inside. Expensive gases can be compressed to high pressure, leading to high incorporation of the endohedral species. Cage closure steps allow preparation of gram‐scale endohedral fullerenes including ^3^He@C_60_, HD@C_60_, and Ne@C_60_.

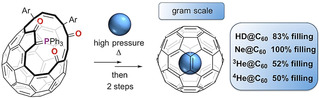

## Introduction

Compounds in which atoms or small molecules (A) are trapped in the cavity of cage fullerenes such as C_60_ are known as endofullerenes and denoted A@C_60_. They are of interest for study of their material properties and the properties of the isolated endohedral species.[^1a^, ^1b^, ^1c^]

The noble gas endofullerenes of C_60_ have been the object of sustained theoretical studies[^2a^, ^2b^, ^2c^, ^2d^, ^2e^, ^2f^, ^2g^, ^2h^, ^2i^, ^2j^, ^2k^] of their geometry, reactivity and electronic structure, and subject to a recent review.^[3]^ Encapsulation of a noble gas in C_60_ was first detected by mass spectrometry in the formation of ^4^He@C_60_ from collision of accelerated C_60_^+.^ with helium gas,^[4]^ and ^4^He@C_60_ was also observed at the part‐per‐million level in C_60_ formed by the carbon arc discharge method using He as the buffer gas.^[5]^ High temperature and pressure exposure of C_60_ to the noble gases allows direct incorporation, and isolation of samples containing approximately 0.1 % of the endohedral atom He, Ne, Ar, or Kr, or 0.03 % of Xe.^[6]^ Incorporation is improved by addition of KCN, to levels of 1 % for He and circa 0.3 % for Ar, Kr, or Xe, albeit at the cost of lower recovery.[^7a^, ^7b^, ^7c^] High‐energy helium bombardment of C_60_ under explosive conditions has also been used for direct encapsulation of the noble gas.^[8]^ Removal of empty C_60_ using many cycles of preparative HPLC has been reported for the heavier noble gas endofullerenes (Ar@C_60_, 1.3 mg, 98 % filled;[^7c^, ^9^] Kr@C_60_, 0.14 mg, 90 % filled;^[10]^ Xe@C_60_ 0.32 mg, 50 % filled^[7b]^), but direct encapsulation methods are not practical for synthesis of larger amounts of a noble gas endofullerene, and cannot be used to prepare molecular endofullerenes such as H_2_@C_60_.

Yet, many applications exist to benefit from the availability of larger‐scale synthetic methods. Noble gas endofullerenes encapsulating the ^3^He or ^129^Xe isotopes, with nuclear spin=1/2 , are potentially valuable as biosensors for detection by magnetic resonance, or as tools to monitor the course of fullerene reactions by NMR.[^11a^, ^11b^, ^11c^, ^11d^] Determining the quantised rotational and translational energy levels of an endohedral species, using inelastic neutron scattering and IR/THz spectroscopy, provides a powerful test of current models of non‐bonding interactions.[^12a^, ^12b^, ^12c^] Endofullerenes in which a trapped molecule exhibits nuclear spin isomerism are of importance for the study of spin isomer interconversion, allotrope enrichment, and have potential applications to chemical and clinical magnetic resonance.^[13]^ Here, nuclear spin conversion in H_2_@C_60_[^14a^, ^14b^, ^14c^, ^14d^] and H_2_O@C_60_[^15a^, ^15b^] has been studied to date.

**Figure 1:**
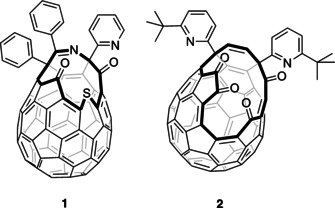
Open‐cage fullerenes used in the reported syntheses of ^4^He@C_60_, H_2_@C_60_, H_2_O@C_60_, and HF@C_60_.

To address these needs, a great deal of progress has been made in the synthesis of endofullerenes by multi‐step routes (termed “molecular surgery”) in which a hole is chemically opened in the fullerene, an atom or small molecule enters the cavity, and the opening is then repaired to restore the original carbon cage with the atom or small molecule entrapped. Following the first insertion of He and H_2_ into an open fullerene by Rubin et al.,^[16]^ cage closure was pioneered by Komatsu and Murata who reported syntheses of H_2_@C_60_ and ^4^He@C_60_ from open fullerene **1**, by insertion of H_2_ directly into **1** (Figure 1), and of ^4^He into the sulfoxide derivative, under high pressure (^4^He at 1230 atm, H_2_ at 800 atm) followed by a series of chemical reactions to re‐form the C_60_ cage.[^17a^, ^17b^, ^17c^] The method allows larger‐scale synthesis of the noble gas endofullerene than is possible using direct encapsulation, 38 mg of ^4^He@C_60_ with 30 % ^4^He filling was obtained. Similarly, the molecular endofullerene H_2_@C_60_ was prepared with 118 mg mass recovery and >90 % H_2_ filling. Murata and co‐workers also developed the synthesis and orifice‐suture of open‐cage fullerene **2** in their preparation of H_2_O@C_60_,^[18]^ and we have since reported an optimised procedure to obtain H_2_O@C_60_, and syntheses of H_2_@C_60_ and HF@C_60_ that also rely upon encapsulation of the endohedral molecule by **2**.[^19a^, ^19b^] Recently, we used an open‐cage fullerene with a larger opening to prepare Ar@C_60_ and CH_4_@C_60_.[^20a^, ^20b^]

We now report a phosphorus ylid derivative of **2** that can be filled in the solid state and the orifice closed in situ by raising the temperature, enabling efficient synthesis of H_2_@C_60_ and ^4^He@C_60_, and their expensive isotopologues HD@C_60_, D_2_@C_60_, and ^3^He@C_60_, as well as the first “molecular surgery” synthesis of Ne@C_60_.

## Results and Discussion

**Figure 2:**
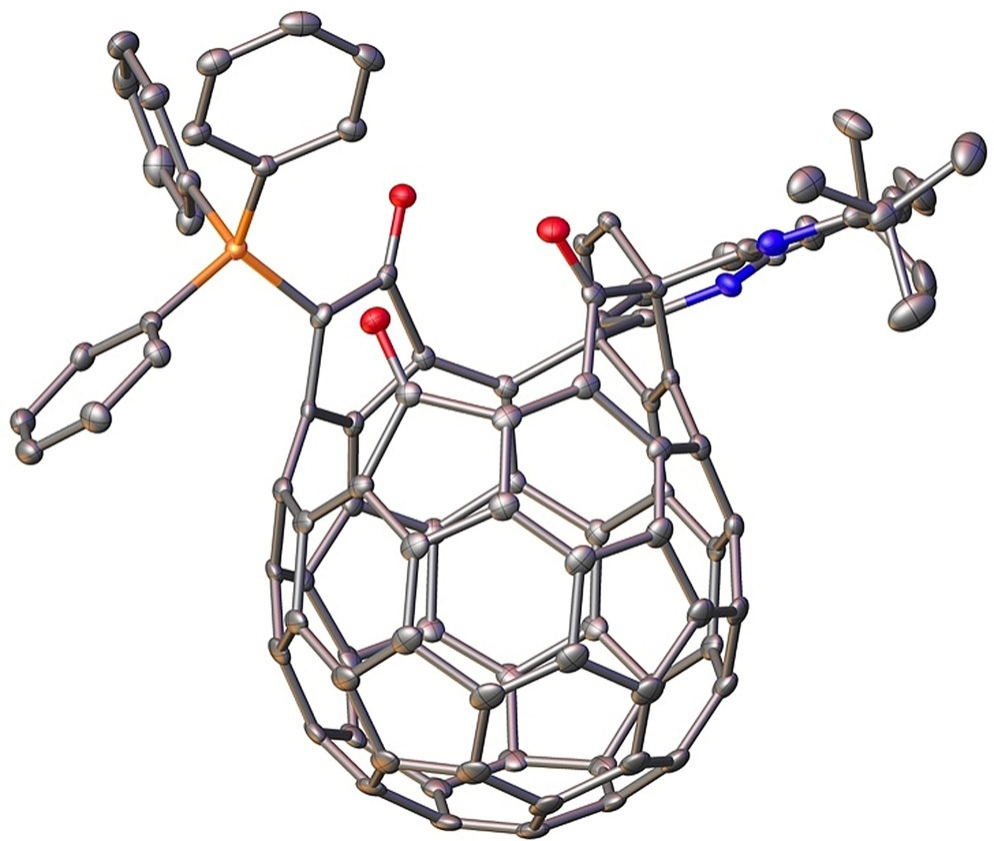
Crystal structure of phosphorus ylid **4**. Thermal ellipsoids are shown at the 50 % probability level and hydrogen atoms are omitted for clarity. Deposition Number 1953259 contains the supplementary crystallographic data for this paper. These data are provided free of charge by the joint Cambridge Crystallographic Data Centre and Fachinformationszentrum Karlsruhe Access Structures service www.ccdc.cam.ac.uk/structures. Structure details are reported in Section S3.1 of the Supporting Information.

**Scheme 1:**
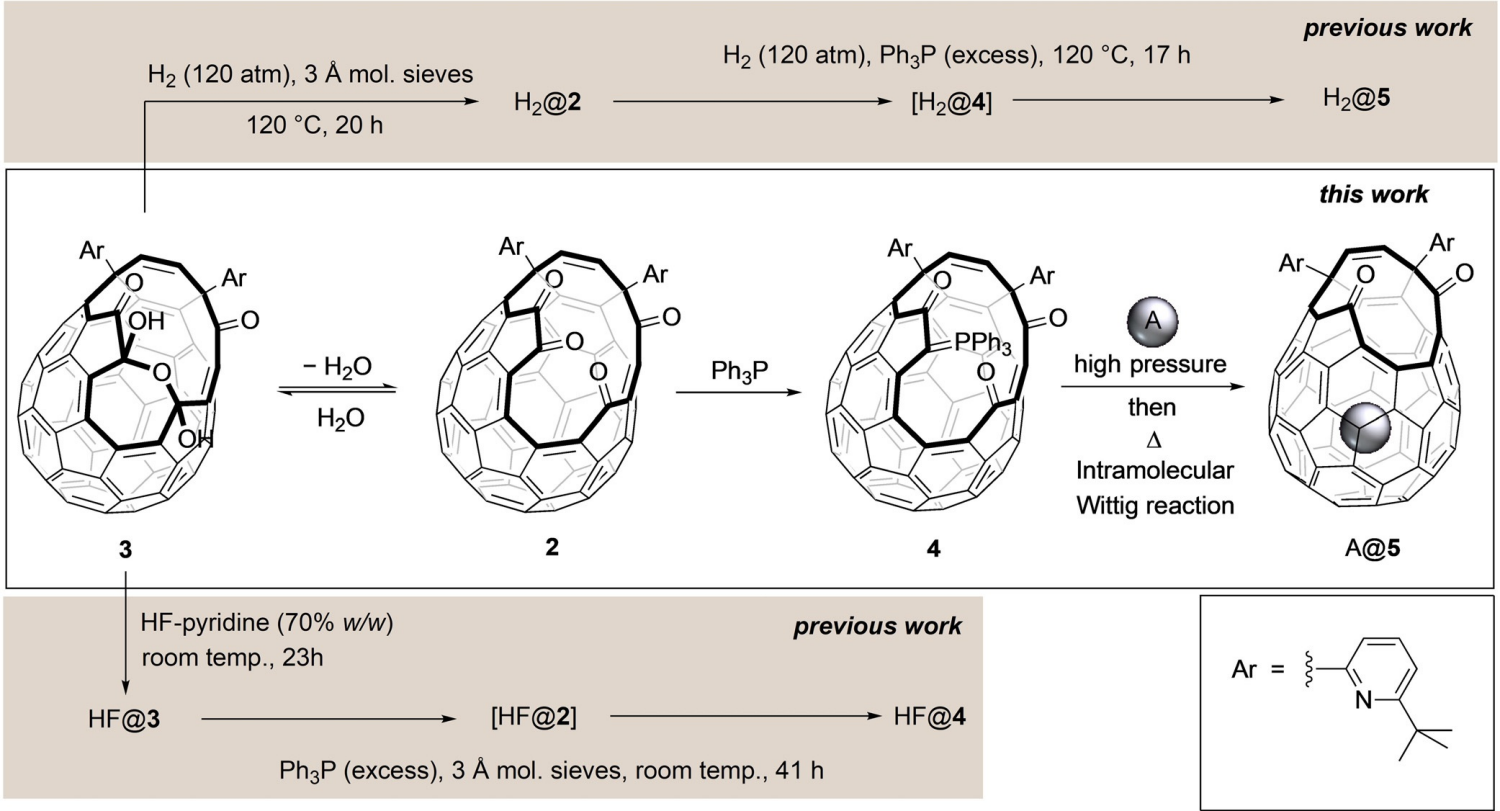
Phosphorus ylid **4** is a precursor in reported syntheses of endofullerenes, including H_2_@C_60_, and may be prepared from known (bis)hemiketal **3** under mild conditions. Stable, and isolable, **4** is a suitable intermediate for encapsulation and in situ entrapment of an endohedral species A.

Our reported synthesis of H_2_@C_60_ involved trapping of H_2_ inside open‐cage fullerene **2**.^[19b]^ In this key step, **2** was formed in situ upon heating its hydrate, bis(hemiketal) **3**, in solution with 3 Å molecular sieves under a high‐pressure atmosphere of H_2_, to give H_2_@**2** with 60 % H_2_ incorporation. Then, heating H_2_@**2** with Ph_3_P induced the first stage of ring closure, giving H_2_@**5**, but also had to be conducted under a high pressure of H_2_ to avoid loss of the endohedral molecule (Scheme 1). We later showed that HF@**3** undergoes dehydration and slow reaction with Ph_3_P at room temperature, to form a stable phosphorus ylid that is an intermediate in the ring closure leading to **5** but, unfortunately, only undergoes the necessary intramolecular Wittig reaction upon heating—leading to complete loss of HF. The structure of the phosphorus ylid, HF@**4**, was suggested by close agreement of a calculated ^13^C NMR spectrum of **4** with the experimental spectrum of the regioisomer shown in Scheme 1,^[19a]^ and we have now confirmed the structure of **4** by X‐ray crystallography (Figure 2). The crystal structure reveals that the C=O bond (1.223 Å) of the carbonyl group adjacent to the phosphorus ylid moiety is longer than those of the remote carbonyl groups (1.209 Å and 1.202 Å), suggesting that the ylid has substantial enolate character which may explain its good stability.^[21]^

If entry of H_2_ or another species into **4** occurred at a temperature lower than that required for the intramolecular Wittig reaction, it would be possible to fill the ylid **4** then induce the Wittig reaction that traps the endohedral species simply by raising the temperature. This would avoid the need to vent gas, add Ph_3_P reagent, and re‐pressurise as in our synthesis of H_2_@C_60_. Comparison of the activation enthalpies for entry of some small atoms and molecules (He, Ne and H_2_) through the 16‐membered openings of **2** and **4** was made by density functional theory calculations and, in each case, the barrier to entry into **4** was only around 10 kJ mol^−1^ higher than that for entry into **2** (Table 1).

**Table 1:** Binding and activation energies for entry and exit of He, Ne, and H_2_ into open fullerenes **2** and **4**.^[a]^

	Δ*H* (binding) [kJ mol^−1^]	Δ*H* ^≠^ (entry) [kJ mol^−1^]	Δ*H* ^≠^ (exit) [kJ mol^−1^]
H_2_+**2 a**⇌H_2_@**2 a**	−22.3	50.6	72.9
He+**2 a**⇌He@**2 a**	−10.4	31.5	41.9
Ne+**2 a**⇌Ne@**2 a**	−20.1	52.8	72.9
H_2_+**4 a**⇌H_2_@**4 a**	−21.0	60.6	81.6
He+**4 a**⇌He@**4 a**	−10.3	39.0	49.4
Ne+**4 a**⇌Ne@**4 a**	−19.2	64.6	83.8

[a] Energies were calculated with density functional theory using the M06‐2X functional and cc‐pVTZ basis set at M06‐2X/cc‐pVDZ geometries. Model structures **2 a** and **4 a**, in which the 6‐*tert*‐butylpyridyl groups are replaced by methyl substituents, were used to represent **2** and **4**. For full details and references see the Supporting Information.

So, under the same conditions of our solution‐phase H_2_ filling of **2** (120 atm of H_2_, 120 °C in 1,2‐dichlorobenzene)^[19b]^ we obtained comparable 62 % H_2_ encapsulation by **4**, implying that closure of **4** does not occur before equilibration of H_2_ between the cavity and outside. Since the Wittig closure reaction is now unimolecular, a solvent should not be necessary and, indeed, heating solid **4** gave **5** in excellent yield. The closure was significantly slower than in solution, for example, heating **4** for 3 h at 120 °C gave 55 % conversion to **5** in 1,2‐dichlorobenzene‐*d*_4_ solution, but only 4 % conversion in the solid state.

At 160 °C closure of solid **4** was complete in less than 1 h, and heating the solid phosphorus ylid **4** under approximately 500 atm H_2_ at 160 °C gave H_2_@**5** in 88 % yield with 80 % encapsulation, demonstrating that endohedral incorporation of the gas is faster than ring closure in the solid state.

Open fullerenes have been filled in the solid state before,[^17b^, ^17c^, ^22a^, ^22b^, ^22c^] but this is the first example where contraction of the cage opening can be carried out in situ, and there are huge practical advantages. The pressure reactor can be small in volume, allowing higher pressures to be used safely, and the volume of gas needed to achieve the high pressure is much smaller—essential if the gas is expensive and/or rare (such as for encapsulation of ^3^He, see below). To apply this method of solid‐state filling and in situ closure for large‐scale synthesis, stainless steel pressure reactors with volumes between 1.2–5.0 mL and with pressure ratings of 2400–4000 atm were constructed as part of a bespoke apparatus for compression of gas using a manual intensifier (see Supporting Information). Results reporting the preparation of A@**5**, where A=Ne and isotopologues of H_2_ and He, are summarised in Table 2, and described in detail below.

**Table 2:** Solid‐state filling of **4** and in situ ring contraction.^[a]^

Entry	Gas A	Pressure [atm]	A@**5** filling factor [%]^[b]^	Yield of isolated A@**5** [%]^[c]^
1	HD	520	83	75
2	D_2_	423	73	72
3	H_2_	1806	95	79
4	^4^He	2374	50	84
5	^3^He	2315	52	79
6	Ne	1742	63	82

[a] All reactions were performed under the stated pressure of gas A, at temperatures in the range 140–186 °C and for 0.75–14 h. For full details see the Supporting Information. [b] Filling factors were calculated by comparison of integrals in the ^1^H NMR spectrum (A=H_2_) or by comparison of peak intensities for the filled and empty species in the ^13^C NMR spectrum (A=Ne, He, HD, or D_2_). [c] Yield of isolated product, following purification by column chromatography.

### H_2_@C_60_, HD@C_60_, and D_2_@C_60_

HD@C_60_ is an interesting isotopologue of H_2_@C_60_ as it lacks the nuclear symmetry, and thus selection rules in the rotational energy levels. HD@C_60_ has been prepared before, and its IR and inelastic neutron scattering (INS) spectra acquired, but as a mixture with H_2_@C_60_ and D_2_@C_60_.[^23a^, ^23b^] Detailed predictions of the variable temperature INS spectra of HD@C_60_ have been made,^[24]^ but to test them experimentally requires a large sample of pure material. Although the use of solid‐state filling helps overcome the problem of the high cost of the gas, the low pressure (10 atm) at which HD is available limits the pressure our intensifier could generate to 80–85 atm. With an initial pressure of 82 atm HD gas at room temperature, heating with **4** at 160 °C for 2 h gave HD@**5** with only 44 % incorporation. The problem was alleviated by cooling the pressure reactor in liquid nitrogen before charging to 119 atm HD. Upon warming to room temperature, a pressure of 420 atm was achieved and at 140 °C, 520 atm. HD@**5** was obtained in 75 % yield and with an 83 % filling factor of HD (Table 2, entry 1). Of concern was the known disproportionation of HD to H_2_+D_2_ in contact with iron.^[25]^ Under the described conditions, disproportionation was limited to <1 %, but when higher temperatures and pressures were used for the filling/closure it became significant (after overnight exposure of **4** to 800 atm of HD at 180 °C, 35 % disproportion, as measured inside the cage, had occurred).

Similarly, cryogenic charging of the pressure reactor enabled heating of phosphorus ylid **4** under 423 atm of D_2_ gas, or circa 1800 atm of H_2_. Respectively, D_2_@**5** was obtained with 73 % filling and H_2_@**5** with 95 % filling, both in good yield (Table 2, entries 2 and 3).

**Scheme 2:**
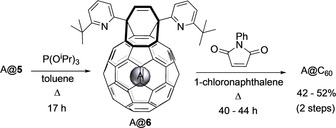
Cage closure of A@**5** to prepare A@C60, where A=Ne, ^3^He, ^4^He H_2_, D_2_, or HD. After reduction to A@**6**, the second step involves sequential [4+2], retro[4+2], and [2+2+2] cycloaddition.^[19b]^

Closure of the cage opening of each compound, HD@**5**, D_2_@**5**, and H_2_@**5**, was carried out according to the two‐step procedure previously reported (Scheme 2).^[19b]^ From a single high‐pressure filling experiment we were easily able to prepare 100–150 mg of HD@C_60_ (83 % filling), D_2_@C_60_ (73 % filling), or H_2_@C_60_ (approx. 95 % filling), and obtain these endofullerenes on gram scale over several batches.

### ^4^He@C_60_ and ^3^He@C_60_

Helium has much less favourable enthalpy of binding into open fullerene **4** than H_2_ (Table 1) so a higher pressure is required for good incorporation. Our pressure intensifier has a limit of 1000 atm, but the cryogenic method described above enabled pressures well above 2000 atm to be generated in the reactor at a temperature of 180 °C. Filling and ring contraction of **4** under almost 2400 atm of ^4^He lead to 50 % helium incorporation in ^4^He@**5** (Table 2, entry 4) while, for comparison, 600 atm gave a filling factor of only 21 %. Synthesis of ^4^He@C_60_ with 30 % ^4^He encapsulation has been reported by Komatsu and Murata,^[17b]^ although the method we describe herein is more easily scalable for preparation of the endofullerene in larger (gram) quantities. The rarity and expense of ^3^He has previously been an obstacle to the preparation of ^3^He@C_60_—only 10 L at STP was available to us. So, a compressor was designed and built to minimise the dead volumes in our existing apparatus, and enabled ^3^He compression into a 2.4 mL capacity pressure reactor using the cryogenic charging method. This gave a pressure in excess of 2300 atm under an elevated temperature of >170 °C for the intramolecular Wittig reaction, and ^3^He@**5** was obtained with 52 % ^3^He filling (Table 2, entry 5). Importantly, we were able to recover >99.8 % of the ^3^He gas back into the apparatus and source cylinder after each experiment. Around 40 % of the unrecovered gas had been encapsulated by the fullerene. Once again, conversion of He@**5** to He@C_60_ was conducted as described in Scheme 2, and >1 g material with circa 50 % filling was prepared for each isotope.

### Ne@C_60_

The DFT calculations given in Table 1 suggested that incorporation of Ne into **2** or **4** should be possible. The synthesis of Ne@C_60_ was first achieved by filling solid fullerene **2** (under Ne gas at 380 atm, 150 °C, 17 h), and isolation of the product as its hydrate, Ne@**3**. The filling factor of Ne@**3** was estimated to be 15–20 % from the ^1^H NMR spectrum. Although an attempt to convert Ne@**3** to Ne@**5** was made using the established method of heating with Ph_3_P at 120 °C, complete loss of neon occurred. Changing the conditions to PhP(2‐furyl)_2_ at 60 °C led to isolation of Ne@**5** without loss of endohedral neon, and Ne@C_60_ was subsequently obtained with 16 % filling following the two‐step procedure of Scheme 2. Next, we examined the solid‐state filling and in situ ring contraction of ylid **4** for preparation of Ne@**5** with higher incorporation of neon. From exposure of **4** to 600 atm Ne at 160 °C for 2 h, Ne@**5** was recovered with approximately 40 % filling estimated from the high resolution ESI+ mass spectrum by comparison of peak intensities for the filled and empty species. Furthermore, using the cryogenic method described earlier a pressure of >1700 atm could be attained, and a filling of 63 % in Ne@**5** was achieved (Table 2, entry 6). As expected, Ne@C_60_ with the same filling factor of 63 % was ultimately obtained, in 43 % yield from Ne@**5**.

**Figure 3:**
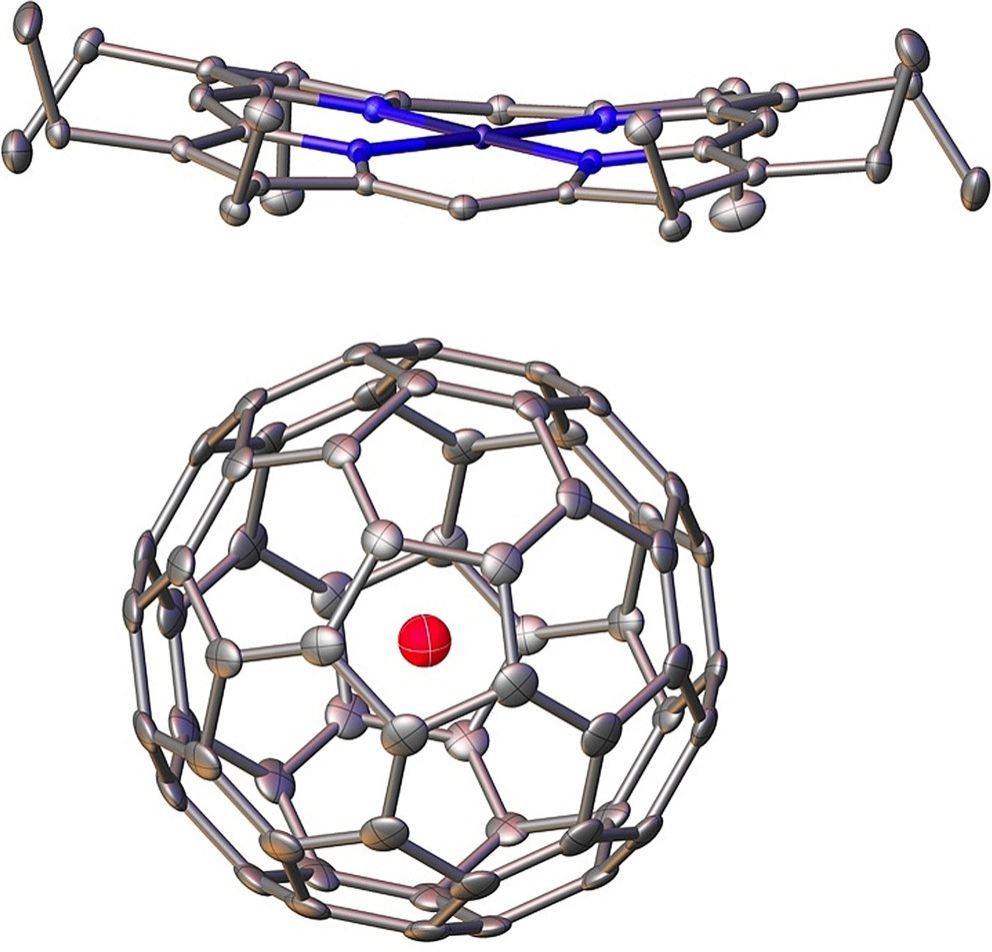
Crystal structure for the nickel(II) octaethylporphyrin/benzene solvate of Ne@C_60_. Thermal ellipsoids are shown at the 50 % probability level. Hydrogen atoms and benzene molecules are omitted for clarity. Deposition Number 1953465 contains the supplementary crystallographic data for this paper. These data are provided free of charge by the joint Cambridge Crystallographic Data Centre and Fachinformationszentrum Karlsruhe Access Structures service www.ccdc.cam.ac.uk/structures. Structure details are reported in Section S3.2 of the Supporting Information.

Ne@C_60_ has been previously prepared only using the direct insertion method, by heating C_60_ at high temperature and pressure with the gas, and resulting in unreported (small) quantities of material with 0.1–0.3 % Ne incorporation.[^6^, ^7a^] With >0.4 g in hand, we carried out enrichment of Ne@C_60_ (63 % filled) to a sample with >99.5 % incorporation of neon, by recycling preparative HPLC, and obtained a crystal structure of the nickel(II) octaethylporphyrin/benzene solvate^[26]^ in which the C_60_ cage is indistinguishable from that of empty C_60_ (Figure 3).^[27]^ The structure shows the neon atom at the centre of the cage.

**Figure 4:**
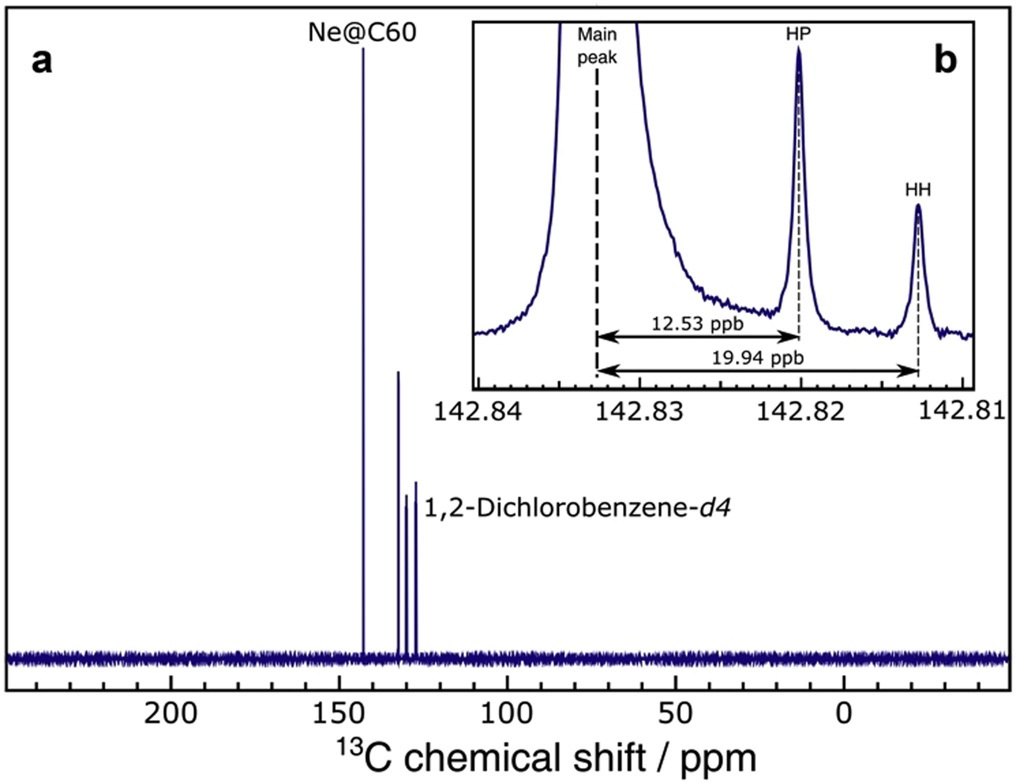
a) ^13^C NMR spectrum of Ne@C_60_ (>99.5 % neon filling), 31–32 mm solution in degassed 1,2‐dichlorobenzene‐*d*_4_ at a field of 176 MHz and 298 K, acquired with 152 transients. There is no visible peak from unfilled C_60_. b) Expanded view of the base of the Ne@C_60_ resonance, acquired at 298 K with 680 transients, to show side peaks arising from minor isotopomers with adjacent ^13^C nuclei that share either a hexagon–pentagon (HP) or hexagon–hexagon (HH) edge.

In the noble gas@C_60_ series studied by ^13^C NMR spectroscopy to date, deshielding of the cage resonance with respect to empty C_60_ increases with the van der Waals radius of the entrapped atom: He@C_60_, Δ*δ*=+0.02 ppm;^[17b]^ Ar@C_60_, Δ*δ*=+0.18 ppm;^[20a]^ Kr@C_60_, Δ*δ*=+0.39 ppm;^[10]^ and Xe@C_60_, Δ*δ*=+0.96 ppm.^[7b]^ Shown in Figure 4 a, the ^13^C NMR resonance of Ne@C_60_ was measured with a chemical shift of *δ*_C_=142.83 ppm in 1,2‐dichlorobenzene‐*d*_4_ at 298 K, deshielded by Δ*δ*=+0.024 ppm relative to empty C_60_. The absence of a visible peak for empty C_60_ attests to the high purity of the Ne@C_60_ sample, and displacement of the ^13^C peak of empty C_60_ by 23.6 ppb in the shielding (negative *δ*) direction, relative to the ^13^C peak of Ne@C_60_, was measured from a mixed sample of Ne@C_60_ and C_60_. The slight deshielding of the cage ^13^C resonance in Ne@C_60_ is within 1.2 ppb of that measured for He@C_60_ (Δ*δ*=+0.025 ppm relative to C_60_), and is much less pronounced than in Ar@C_60_ and the heavier noble gas endofullerenes.

Figure 4 b shows two side peaks with an intensity ratio of 2:1 that are assigned to minor isotopomers of Ne@C_60_ that each contain a pair of neighbouring ^13^C nuclei separated by one bond.^[28]^ Two peaks are observed since there are two types of carbon−carbon bond in C_60_, either a hexagon–pentagon (HP) or shorter hexagon–hexagon (HH) shared edge, present in a 2:1 ratio, respectively. The shifts of the side peaks relative to the main Ne@C_60_ resonance correspond to one‐bond isotope shifts of ^1^Δ_HP_=12.53±0.01 ppb for the inner peak, and ^1^Δ_HH_=19.94±0.03 ppb for the outer peak. Side peaks of this kind were also observed for the helium endofullerenes, ^3^He@C_60_ and ^4^He@C_60_.^[29]^

### Large‐scale synthesis of phosphorus ylid 4

**Scheme 3:**
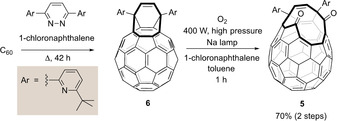
Optimised cage opening of C_60_.

The methods described above allow the filling of large amounts of open‐cage fullerene **4** with the various endohedral species described. Synthesis of bis(hemiketal) **3**, the precursor to **4**, is well described by Murata[^18^, ^30^] but optimisation of the early cage‐opening steps was necessary to more efficiently supply material for our scaled‐up filling procedure (Scheme 3). After opening the C_60_ cage by reaction with 3,6‐bis(6‐(*tert*‐butyl)pyridin‐2‐yl)pyridazine,^[18]^ the existing method for expanding the orifice by photo‐oxygenation with singlet oxygen required irradiation of **6** in a mixture of CS_2_ (flashpoint circa 30 °C, auto‐ignition temperature 100 °C) and 1‐chloronaphthalene, using a 500 W Xenon lamp as O_2_ is passed through the mixture over 23 h. Although recent improvements in both the reaction yield and safety have resulted from use of an LED light source and replacement of CS_2_ with CCl_4_, a reaction time of 48 h is required (from 3 g C_60_).^[30]^

We found that use of 2 molar equiv of C_60_ (relative to the pyridazine reagent) in the first (cycloaddition) step gave a cleaner reaction—presumably by suppressing the formation of multiple addition products. Simple dilution of the product reaction mixture with toluene and direct transfer to the photoreaction vessel enabled the excess remaining C_60_ to serve as an efficient photosensitizer for the formation of ^1^O_2_, and the reaction time of photo‐oxygenation was correspondingly reduced. Indeed, use of a 400 W high‐pressure sodium lamp in a water‐cooled immersion well led to completion of the photochemical reaction in just 1 h (on a scale using 10 g C_60_). Excess C_60_ was readily recovered during purification of **5** by column chromatography and the overall yield was 70 %, based on the portion of C_60_ that was consumed in the reaction (see Supporting Information). Conversion of **5** to bis(hemiketal) **3** is carried out by oxidative cleavage using 4‐methylmorpholine 4‐oxide following the published procedure,^[18]^ and **3** is converted to phosphorus ylid **4** upon treatment with 16 molar equiv Ph_3_P at 60 °C.^[19a]^ For this final step, an excellent and reproducible yield (85–90 %) of **4** is achieved at multi‐gram scale. Overall, the improved methods allow a 10 g batch of ylid **4** to be prepared in a few days.

## Conclusion

Optimised synthesis and solid‐state filling of a phosphorus ylid **4** with H_2_, He, or Ne, with in situ contraction of the cage opening via an intramolecular Wittig reaction, traps the gas inside. Large amounts of material can be processed in a small pressure reactor, allowing the use of high‐pressure conditions and expensive gases. Further transformations gave up to 1 g each of endofullerenes ^3^He@C_60_, ^4^He@C_60_, Ne@C_60_, H_2_@C_60_, D_2_@C_60_, and HD@C_60_, which will enable experimental studies of these interesting species, for example using NMR, IR, and inelastic neutron‐scattering spectroscopy. These studies are underway.

The new availability of a large quantity of ^3^He@C_60_ offers the potential to extend its use in magnetic resonance imaging, where ^3^He gas is already used to visualise air passages in the lung by attachment to other species—although the development of methods for hyperpolarisation of the ^3^He nucleus will be needed to achieve this practical application. The first synthesis of pure HD@C_60_ will permit the rotational and translational energy levels to be determined by INS, and compared with the established predictions as a test of these theoretical methods.

Synthesis of pure Ne@C_60_ completes the series of noble gas endofullerenes to be prepared in macroscopic quantities (up to Xe@C_60_). The absolute chemical shift, and relative endohedral shift, of the cage ^13^C NMR resonance of Ne@C_60_ indicates very similar interactions between the noble gas atom and the fullerene cage to that in He@C_60_.

## Conflict of interest

The authors declare no conflict of interest.

## Supporting information

As a service to our authors and readers, this journal provides supporting information supplied by the authors. Such materials are peer reviewed and may be re‐organized for online delivery, but are not copy‐edited or typeset. Technical support issues arising from supporting information (other than missing files) should be addressed to the authors.

Supplementary
