# Supplementary material for: A Solid‐State Intramolecular Wittig Reaction Enables Efficient Synthesis of Endofullerenes Including Ne@C60, 3He@C60, and HD@C60
Source: Angew Chem Int Ed Engl. 2021 Mar 4;60(16):8960–6. doi: 10.1002/anie.202100817 (PMC8048630; doi:10.1002/anie.202100817)
Supplement: Supplementary file 1 — Supplementary [file ANIE-60-8960-s001.pdf]

Angewandte Chemie
Eine Zeitschrift der Gesellschaft Deutscher Chemiker

Supporting Information

A Solid-State Intramolecular Wittig Reaction Enables Efficient Synthesis of Endofullerenes Including $Ne@C_{60}$, $^3He@C_{60}$, and $HD@C_{60}$

*Gabriela Hoffman, Mark C. Walkey, John Gräsvik, George R. Bacanu, Shamim Alom, Sally Bloodworth, Mark E. Light, Malcolm H. Levitt, and Richard J. Whitby**

anie_202100817_sm_miscellaneous_information.pdf

CONTENTS

			Page
S1.	Experimental procedures and characterisation data		S2
	S1.1	General methods	S2
	S1.2	High pressure apparatus	S2
	S1.3	Synthesis of phosphorus ylid **4** from C_{60}	S4
	S1.4	Solid-state filling of **4** with *in situ* trapping of the endohedral species	S5
		S1.4.1 Synthesis of HD@**5**	S5
		S1.4.2 Synthesis of D_2@**5**	S7
		S1.4.3 Synthesis of H_2@**5**	S9
		S1.4.4 Synthesis of ^{4}He@**5**	S10
		S1.4.5 Synthesis of ^{3}He@**5**	S12
		S1.4.6 Synthesis of Ne@**5**	S14
	S1.5	Cage closure of A@**5**: preparation of endohedral fullerenes A@C_{60}	S16
		S1.5.1 General procedure	S16
		S1.5.2 Synthesis of HD@C_{60}	S17
		S1.5.3 Synthesis of D_2@C_{60}	S18
		S1.5.4 Synthesis of H_2@C_{60}	S19
		S1.5.5 Synthesis of ^{4}He@C_{60}	S20
		S1.5.6 Synthesis of ^{3}He@C_{60}	S21
		S1.5.7 Synthesis of Ne@C_{60}	S22
		S1.5.8 UV-vis spectra of endohedral fullerenes A@C_{60}	S24
S2.	Density Functional Theory calculations		S24
S3.	Crystallography data		S25
	S3.1	X-Ray structure determination of phosphorus ylid **4**	S25
	S3.2	X-Ray structure determination of **Ne@C_{60}**	S44
S4.	References for the supporting information		S56
S5.	Author contributions		S57

S1. Experimental procedures and characterisation data

S1.1 General methods

Reactions were performed under nitrogen atmosphere using standard Schlenk and syringe techniques, except high-pressure filling reactions which were conducted under an atmosphere of Ne, ^{3}He, ^{4}He, H_2, D_2 or HD at the pressure stated in section S1.4, using the compression apparatus described in section S1.2. Glassware was dried overnight in a hot oven (160 °C) and cooled in a sealed desiccator over silica gel. Reactions were monitored by TLC using Merck silica gel 60 F254 plates, by HPLC performed on a Cosmosil™ Buckyprep analytical column with detection at 326 nm or by NMR spectroscopy. Flash column chromatography was conducted using Merck Geduran Si60 (40-63 μm) silica gel as the stationary phase. Toluene and THF were freshly distilled from sodium benzophenone ketal under nitrogen. Triisopropyl phosphite and technical grade 1-chloronaphthalene (≥ 85%) were distilled over activated molecular sieves under reduced pressure, and solutions in 1-chloronaphthalene were degassed under dynamic vacuum (0.4 mm Hg) until the evolution of gases stopped. All other solvents and reagents were used as received from the commercial supplier without further purification.

Argon, ^{4}He, Ne, H_2, and D_2 were CP quality gases from BOC. ^{3}He was >99.9 atom%, 99.999% pure gas from BOC. HD was >97% D from Cambridge Isotopes Ltd.

NMR spectra were recorded on a Bruker AVII400 FT-NMR or AVIIIHD500 FT-NMR spectrometer, or Bruker Ascend 700 NB magnet with Bruker AVANCE NEO console in the indicated solvent at 298 K. ^{1}H chemical shifts are reported as values in ppm referenced to residual solvent. ^{1}H NMR spectra collected in 1,2-dichlorobenzene-d_4 are referenced to residual solvent at δ_H = 6.93 ppm; and this solvent chemical shift is referenced to TMS (δ_H = 0.00 ppm). The following abbreviations are used to assign multiplicity and may be compounded: s = singlet, d = doublet, t = triplet, q = quartet and m = multiplet. Coupling constants, J, are measured in Hertz (Hz). ^{13}C NMR spectra were ^{1}H decoupled and are referenced to 1,2-dichlorobenzene-d_4 at δ_C = 127.19 ppm (centre of the 1:1:1 triplet) or $CDCl_3$ at δ_C = 77.160 ppm (centre of the 1:1:1 triplet); the solvent chemical shift is referenced to TMS (δ_C = 0.00 ppm). The confidence limits of the absolute chemical shift are dominated by the chemical shift of reference solvents, reported to 2 d.p. for ^{1}H NMR and 3 d.p. for ^{13}C NMR in order to distinguish near-overlapping peaks.

Atmospheric pressure photoionisation (APPI) mass spectra were recorded using the procedure we have recently reported.[1] Positive ion electrospray mass spectra were recorded using a MaXis time of flight (TOF) mass spectrometer (Bruker Daltonik GmbH, Bremen, Germany).

UV measurements were recorded on an Ocean Optics DH-2000-BAL spectrometer with a 1 cm path length cell.

3,6-Bis(6-(*tert*-butyl)pyridin-2-yl)pyridazine was prepared according to the published method.[2]

S1.2 High pressure apparatus

The filling procedures described in section S1.4 were carried out using a bespoke high pressure apparatus constructed from 1/4” o.d., 1/16” i.d. 316L steel tube using Sitec (www.sitec-hp.ch) high pressure cone and thread connections, with T’s (720.1633), crosses (720.1634) and high pressure valves (710.6310) also from Sitec. Valves from Autoclave Engineers and components from Swagelok were used in the low pressure parts of the apparatus. The dead volume was

reduced using a 1.5 mm stainless steel rod inserted into the 1/16” (1.59 mm) i.d. steel tube. A custom 30 mL manual pump from Sitec (model 759.1100 – as 750.1100 but with internal dead volume reduced to ~1.7 mL, 1000 atm rating) was used to increase the pressure of the source gas (figure S1.2(a)).

The high-pressure reactor was constructed from a 100 mm length of 316L steel coned and threaded tubing with 3/8” external diameter (9.52 mm) and 5.2 mm internal diameter (pressure rating 2400 atm) with a blank (720.2114) at one end and adapter (720.1620) to 1/4” steel tube at the other (figure S1.2(b)). A cut down 5 mm o.d. pyrex NMR tube was usually used to contain the sample of solid open-fullerene **4**, although direct packing of the reactor could be used to increase the mass of **4** which could be accommodated.

Pressure was constantly monitored using a Wika model HP-2 pressure sensor (hydrogen compatible, when appropriate) connected to a Hengstler 0735A20000 LED display. An oven modified from a Kugelrohr distillation apparatus and fitted with a Sestos model DIS temperature controller and PT100 thermocouple, was used to heat the reactor. Thermocouples were also attached to the reactor, at each end and in the centre, and the temperature at each position was constantly recorded using a Pico technology USB TC-08 data logger attached to a PC. The reaction temperature stated for each procedure in section S1.4 is from the sensor attached to the centre of the reactor tube.

To achieve higher final pressures the bomb was sometimes cooled in liquid nitrogen while filling (figure S1.2(c)).

The system allowed evacuation to <0.2 mbar before use. When using ^3He gas, following completion of the reaction, as much gas as possible was pumped from the reactor back into the source cylinders, and any residual gas recovered into previously evacuated 1L cylinders. Also with ^3He filling additional components allowed the loaded bomb to be pressure tested with ^4He before evacuation and charging with ^3He.

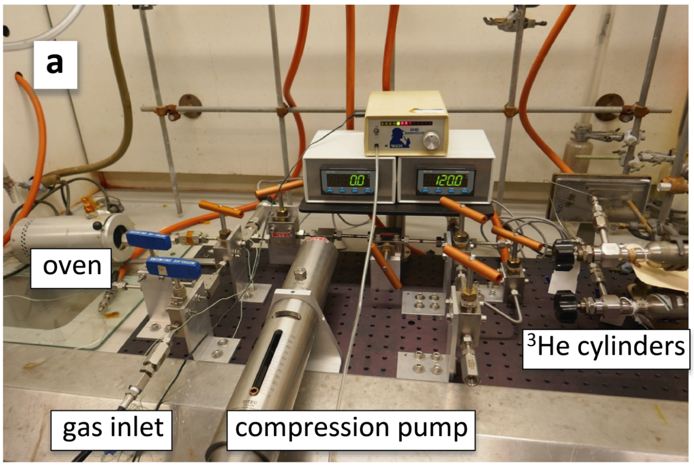

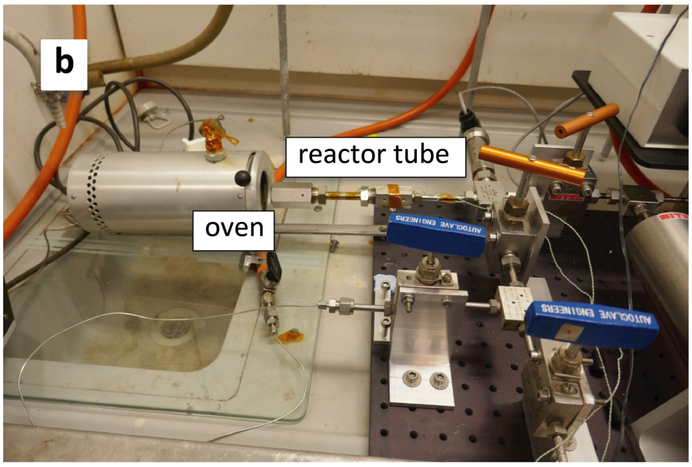

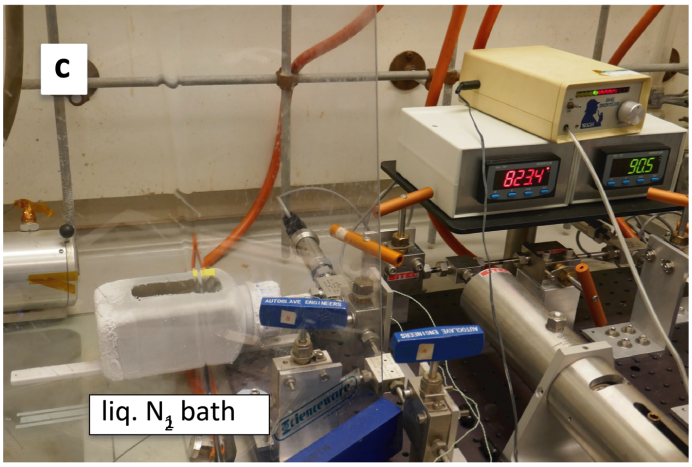

Figure S1.2 (a) High pressure apparatus for solid state filling described in section S1.4. The configuration shown is for ^3He filling. (b) Close-up of the reactor, oven and components for pressure testing of the reactor with ^4He, as well as recovery of ^3He. (c) The reactor being filled using the Sitec manual pump, while cooled with liquid nitrogen.

S1.3 Synthesis of phosphorus ylid 4 from C_{60}

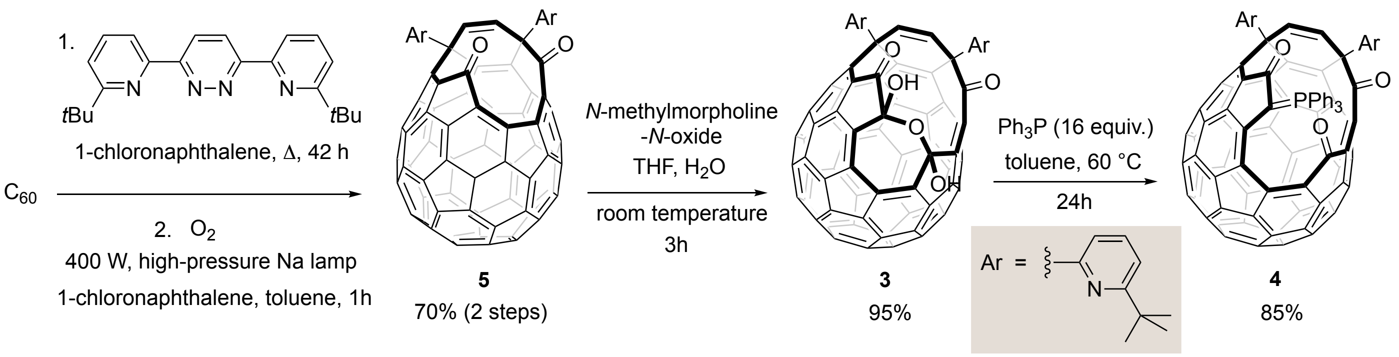

A solution of C_{60} (6.821 g, 9.473 mmol) and 3,6-bis(6-(*tert*-butyl)pyridin-2-yl)pyridazine (1.621 g, 4.678 mmol) in 1-chloronaphthalene (200 mL) was degassed under dynamic vacuum (approx. 0.2 mm Hg) and sonication. The stirred solution was then warmed to reflux for 42 h under N_2, before cooling to room temperature and dilution with toluene (150 mL). This mixture was transferred to a jacketed photo reactor and the water-cooled solution was irradiated with a high-pressure Na lamp (400 W), placed in the middle of the reactor at an approximate distance of 2 cm from the solution, bubbling oxygen at a continuous flow rate of approx. 20 mL min^{-1} for 1 h. The reaction mixture was then diluted with hexane (200 mL) and poured directly over a silica column (7 cm diameter column, 600 mL of SiO_2) packed with hexane:toluene (1:1).

Using an eluent gradient of hexane:toluene (1:1) → 100% toluene, a purple band containing unreacted C_{60} and 1-chloronaphthalene was first collected. From quantitative HPLC assay, the amount of recovered C_{60} was calculated to be 4.06 g (60%), and the quantity of C_{60} not recovered was 2.76 g (3.83 mmol). Solvents were removed by vacuum distillation and residual C_{60} was washed with Et_2O (3 × 30 mL) before drying *in vacuo*. Recovered C_{60} was of sufficient purity to be used in a repeat reaction.
A second band containing material with Rf = 0.5 in toluene was collected, and removal of solvents *in vacuo* gave open-fullerene **5** as a brown powder (2.848 g, 70% based on C_{60} consumption). Spectroscopic data were consistent with the published data.[2]

Conversion of **5** to bis(hemiketal) **3** was carried out according to the published method.[2]

Conversion of bis(hemiketal) **3** (5.300 g, 4.727 mmol) to phosphorus ylid **4** (5.403 g, 85%) was carried out by scale-up of our previously reported procedure.[3] The structure of **4** was confirmed by X-ray crystallography as described in section S3.1, and all spectroscopic data were consistent with those previously reported.

S1.4 Solid-state filling of 4 with *in situ* trapping of the endohedral species

S1.4.1 Synthesis of HD@5

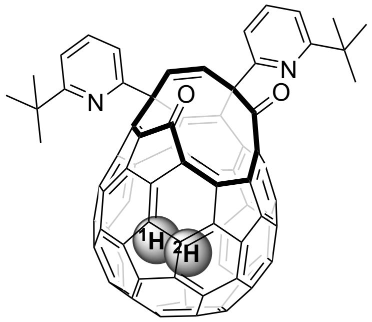

A thin-walled pyrex tube was charged with solid open-fullerene **4** (0.726 g, 0.538 mmol) and inserted into a steel high-pressure reactor equipped with pressure intensifier (section S1.2). The reactor was degassed under dynamic vacuum (approx. 0.2 mm Hg) before cooling to −196 °C using liquid nitrogen and charging with HD gas to 26 atm. The gas was then compressed in the reactor to 119 atm, before allowing to warm to room temperature at which point the pressure reached 420 atm. The reactor was then heated to 140 °C and maintained at this temperature for 14 h, with a stable internal pressure of 520 atm during this time. After cooling to room temperature and slow release of the pressure, the solid residue was purified by flash column chromatography (SiO_2 eluted with toluene). Material with Rf = 0.5 in toluene was collected, and removal of solvents *in vacuo* gave the title compound HD@**5** as a brown powder (0.432 g, 75 %) with 83% HD filling.

83% HD filling of HD@**5** is based on the same filling of the HD@C_{60} material obtained from ring-closure of this sample (section S1.5.2). No spectroscopic data were collected for 83% filled HD@**5**, as a sample of 44% filled HD@**5** had already been characterised. This data is reported below.

^{1}H NMR (500 MHz, $CDCl_3$) δ = 7.68 (t, J = 7.8 Hz, 1H), 7.64 (t, J = 7.8 Hz, 1H), 7.51 (dd, J = 7.8, 0.8 Hz, 1H), 7.38 (dd, J = 7.8, 0.8 Hz, 1H), 7.24 (dd, J = 7.9, 0.9 Hz, 2 × 1H separated by 0.002 ppm), 7.22 (d, J = 9.9 Hz, 0.44H HD filled), 7.22 (d, J = 9.9 Hz, 0.56H empty), 7.17 (d, J = 9.9 Hz, 0.44H HD filled), 7.17 (d, J = 9.9 Hz, 0.56H empty) 1.26 (s, 9H), 1.18 (s, 9H), −5.74 (t, J = 41.8 Hz, 0.44H endohedral HD) ppm.

^{13}C NMR (126 MHz, $CDCl_3$) δ = 200.427, 192.345, 169.201, 168.850, 165.408, 162.204, 154.906, 150.189, 148.916, 148.023, 148.019, 147.851, 147.510, 147.040, 146.733, 146.721, 146.621, 146.440, 146.240, 146.169, 146.141, 146.078, 145.992, 145.954, 145.868, 145.702, 145.531, 145.231, 145.165, 144.833, 144.822, 144.400, 144.387, 144.102, 143.071, 142.822, 142.652, 142.361, 142.178, 142.043, 141.822, 141.126, 140.812, 140.758, 140.624, 140.332, 140.205, 139.956, 139.744, 139.642, 139.551, 138.905, 138.579, 138.516, 137.523, 137.466, 137.208, 136.569, 136.468, 136.054, 134.992, 133.946, 133.039, 132.948, 132.670, 132.638, 131.680, 129.844, 120.107, 119.692, 117.699, 116.914, 61.104, 54.918, 37.930, 37.837, 30.086, 30.068 ppm. The peaks arising from empty open-fullerene **5** are present but not reported.

HRMS (ESI+) (*m/z*): Calcd. for $[^{12}C_{82}{}^{1}H_{28}{}^{2}HN_2O_2]^+$ (HD@**5**+H^+) 1074.2286; found 1074.2261.

IR: ν = 2950, 1740, 1567, 1442 cm^{-1}.

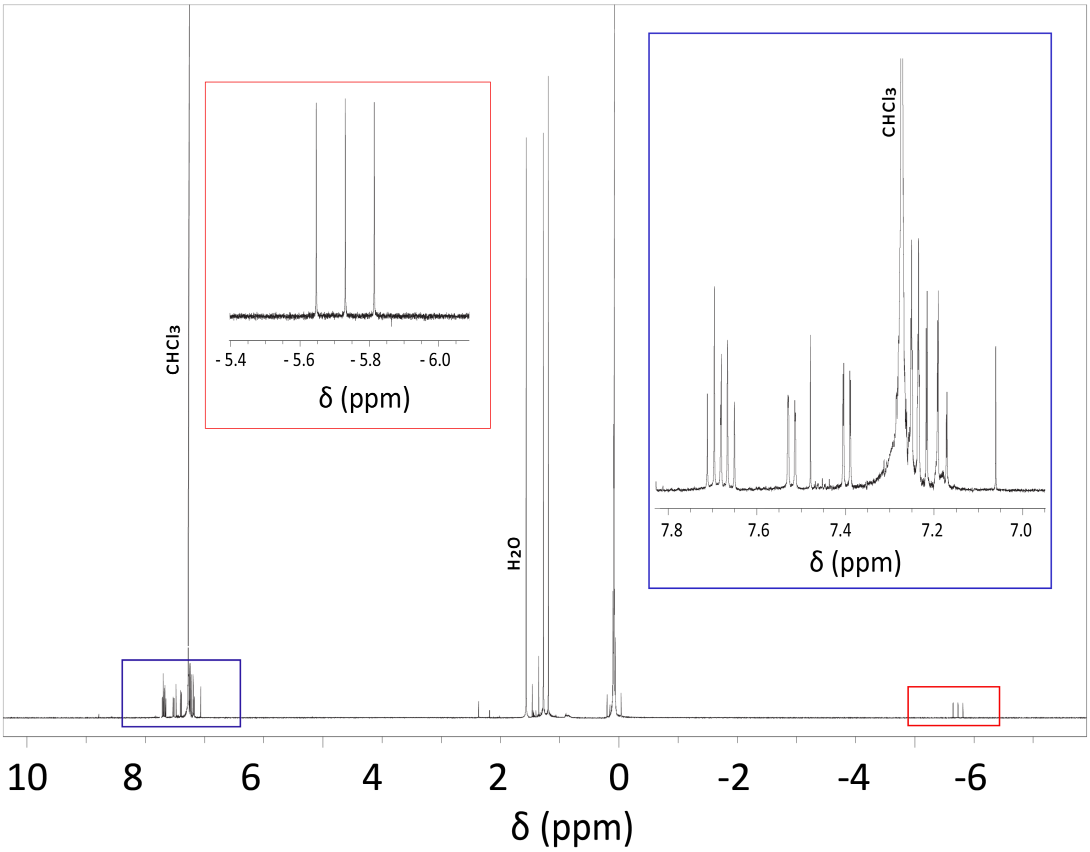

Figure S1.4.1.1 ^{1}H NMR (500 MHz, $CDCl_3$) of HD@**5** (44% filling).

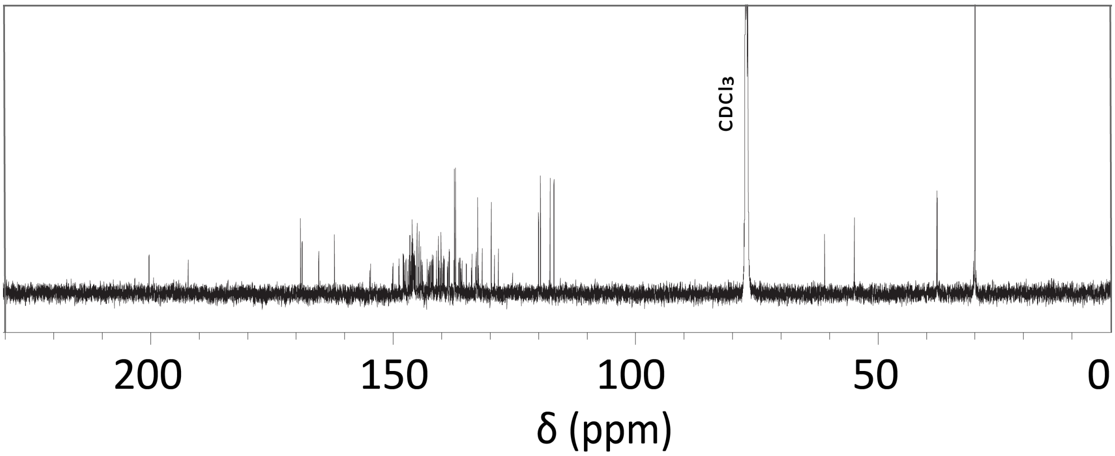

Figure S1.4.1.2 ^{13}C NMR (126 MHz, $CDCl_3$) of HD@**5** (44% filling).

S1.4.2 Synthesis of D_2@5

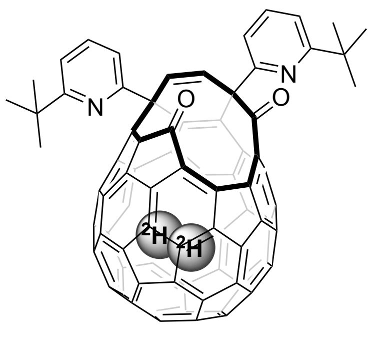

A thin-walled pyrex tube was charged with solid open-fullerene **4** (0.616 g, 0.456 mmol) and inserted into a steel high-pressure reactor equipped with pressure intensifier (section S1.2). The reactor was degassed under dynamic vacuum (approx. 0.2 mm Hg) before cooling to −196 °C using liquid nitrogen and charged with D_2 gas to 17 atm. The gas was then compressed to 83 atm, before allowing the reactor to warm to room temperature, at which point the pressure reached 314 atm. The reactor was then heated to 176 °C and maintained at this temperature for 1.5 hours, with a stable internal pressure of 423 atm during this time. After cooling to room temperature and slow release of the pressure, the solid residue was purified by flash column chromatography (SiO_2 eluted with toluene). Material with Rf = 0.5 in toluene was collected, and removal of solvents *in vacuo* gave D_2@**5** as a brown powder (0.352 g, 72%).
73% D_2 filling of D_2@**5** was calculated from the ^{13}C NMR spectrum, and the same filling of $D_2@C_{60}$ was obtained from ring-closure of this sample (section S1.5.3).

^1H NMR (500 MHz, $CDCl_3$) δ = 7.68 (t, J = 7.8 Hz, 1H), 7.65 (t, J = 7.8 Hz, 1H), 7.51 (dd, J = 7.8, 0.8 Hz, 1H), 7.39 (dd, J = 7.8, 0.8 Hz, 1H), 7.24 (dd, J = 7.9, 0.9 Hz, 2 × 1H separated by 0.002 ppm), 7.22 (d, J = 10.0 Hz, 0.73H D_2 filled), 7.22 (d, J = 10.0 Hz, 0.27H empty), 7.17 (d, J = 10.0 Hz, 0.73H D_2 filled), 7.17 (d, J = 10.0 Hz, 0.27H empty), 1.26 (s, 9H), 1.18 (s, 9H) ppm.
^{13}C NMR (126 MHz, $CDCl_3$) δ = 200.426, 192.344, 169.202, 168.850, 165.422, 162.207, 154.904, 150.189, 148.918, 148.023, 148.018, 147.850, 147.509, 147.039, 146.734, 146.720, 146.624, 146.438, 146.242, 146.168, 146.142, 146.080, 145.991, 145.956, 145.866, 145.703, 145.531, 145.232, 145.166, 144.832, 144.822, 144.400, 144.387, 144.103, 143.071, 142.822, 142.651, 142.361, 142.176, 142.042, 141.822, 141.126, 140.811, 140.756, 140.625, 140.331, 140.204, 139.957, 139.746, 139.642, 139.551, 138.904, 138.580, 138.517, 137.524, 137.446, 137.208, 136.568, 136.466, 136.053, 134.992, 133.946, 133.038, 132.948, 132.669, 132.637, 131.682, 129.845, 120.107, 119.693, 117.700, 116.915, 61.105, 54.919, 37.932, 37.838, 30.087, 30.070 ppm. The peaks arising from empty open-fullerene **5** are present but not reported.
HRMS (ESI+) (*m/z*): Calcd. for $[^{12}C_{82}{}^{1}H_{27}{}^{2}H_2N_2O_2]^+$ (D_2@**5**+H^+) 1075.2349; found 1075.2336..
IR: ν = 2957, 1738, 1567, 1441 cm^{-1}.

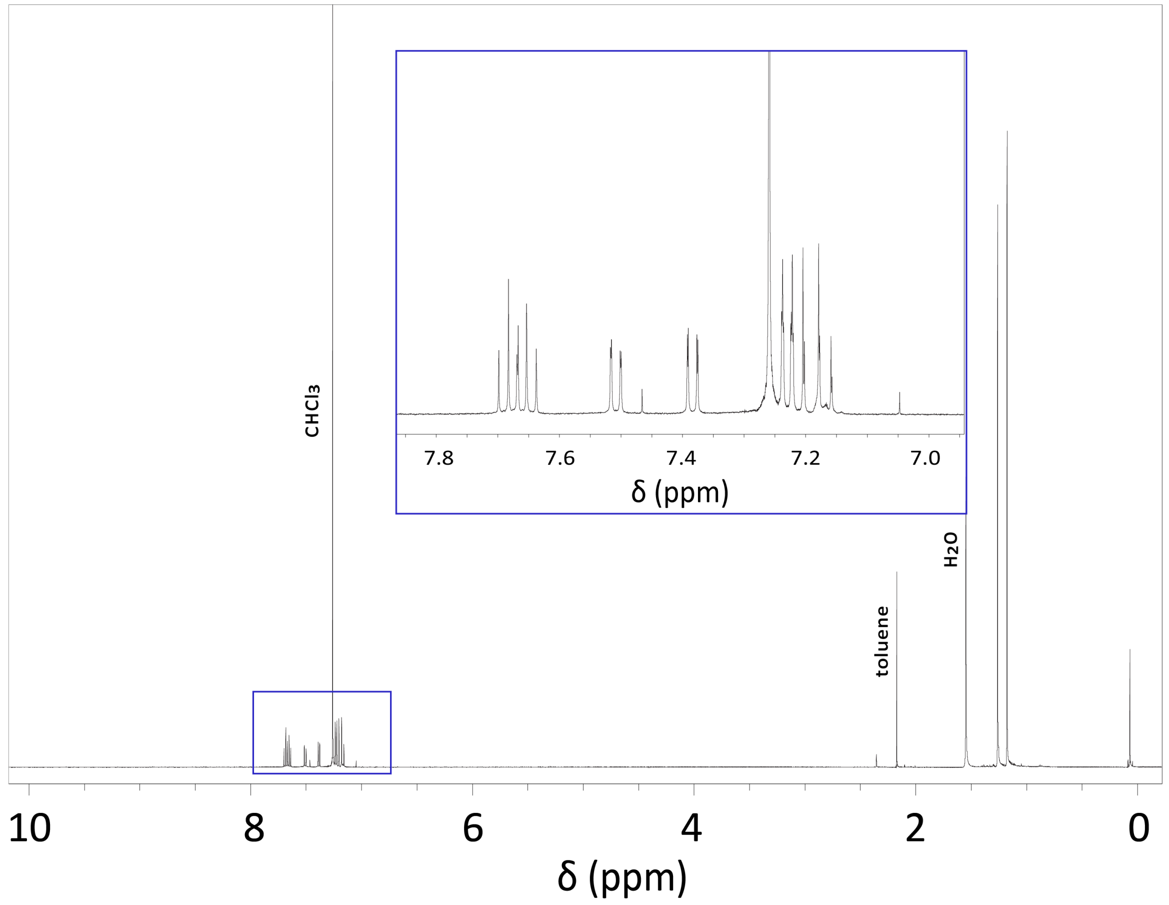

Figure S1.4.2.1 ^{1}H NMR (500 MHz, $CDCl_3$) of D_2@**5** (73% filling).

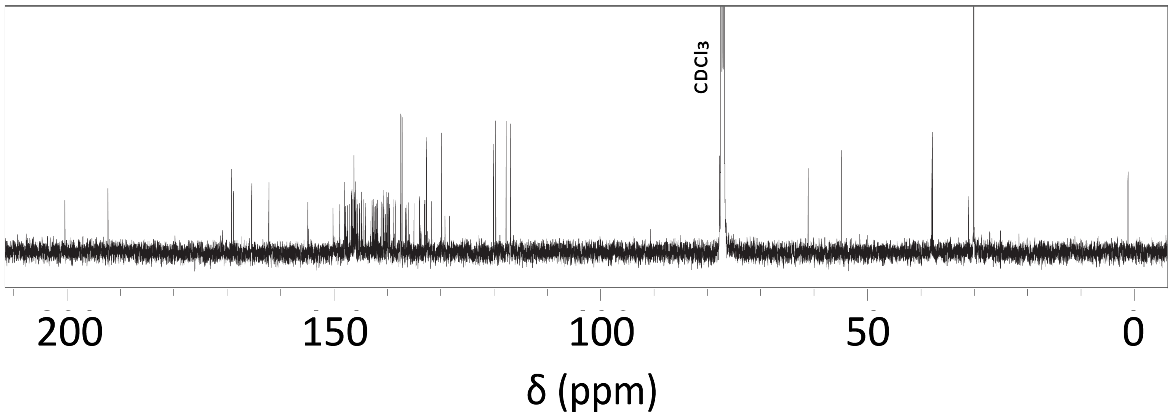

Figure S1.4.2.2 ^{13}C NMR (126 MHz, $CDCl_3$) of D_2@**5** (73% filling).

S1.4.3 Synthesis of $H_2@$5

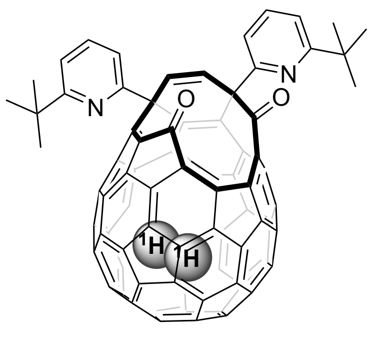

A thin-walled pyrex tube was charged with solid open-fullerene **4** (0.556 g, 0.412 mmol) and inserted into a steel high-pressure reactor equipped with pressure intensifier (section S1.2). The reactor was degassed under dynamic vacuum (approx. 0.2 mm Hg) and then charged with H_2 gas to 121 atm. The reactor was then cooled to −196 °C using liquid nitrogen and the gas compressed to 700 atm. The reactor was warmed to room temperature, at which point the pressure reached 1384 atm, then heated to 186 °C and maintained at this temperature for 2 hours, with a stable internal pressure of 1806 atm during this time. After cooling to room temperature and slow release of the pressure, the solid residue was purified by flash column chromatography (SiO_2 eluted with toluene). Material with Rf = 0.5 in toluene was collected, and removal of solvents *in vacuo* gave $H_2@$**5** as a brown powder (0.351 g, 79%).

>95% H_2 filling was calculated from the ^{1}H NMR spectrum.

^{1}H NMR (400 MHz, $CDCl_3$) δ = 7.68 (t, J = 7.8 Hz, 1H), 7.65 (t, J = 7.8 Hz, 1H), 7.51 (dd, J = 7.8, 0.8 Hz, 1H), 7.39 (dd, J = 7.8, 0.8 Hz, 1H), 7.24 (dd, J = 7.9, 0.9 Hz, 2 × 1H separated by 0.002 ppm), 7.22 (d, J = 10.0 Hz, 0.95H H_2 filled), 7.22 (d, J = 10.0 Hz, 0.05H empty), 7.17 (d, J = 10.0 Hz, 0.95H H_2 filled), 7.17 (d, J = 10.0 Hz, 0.05H empty), 1.26 (s, 9H), 1.18 (s, 9H), 1.18 (s, 9H), −5.71 (s, 1.9H endohedral H_2 >95% filled) ppm.

All spectroscopic data were consistent with those we have previously published for a 60% filled sample of $H_2@$**5**.[4]

S1.4.4 Synthesis of ^{4}He@5

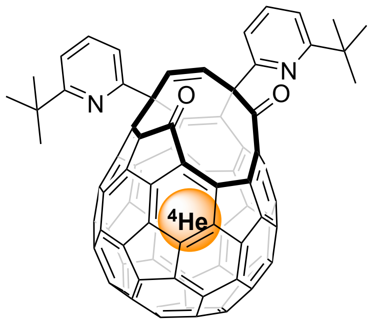

A thin-walled pyrex tube was charged with solid open-fullerene **4** (0.494 g, 0.366 mmol) and inserted into a steel high-pressure reactor equipped with pressure intensifier (section S1.2). The reactor was degassed under dynamic vacuum (approx. 0.2 mm Hg) and then charged with He gas to 72 atm. The gas was compressed to 439 atm, and the reactor was then cooled to −196 °C using liquid nitrogen. Following further compression to 762 atm, the reactor was warmed to room temperature, at which point the pressure reached 1828 atm, and then heated to 180 °C and maintained at this temperature for 2 h, with a stable internal pressure of 2374 atm during this time. After cooling to room temperature and slow release of the pressure, the solid residue was purified by flash column chromatography (SiO_2 eluted with toluene). Material with Rf = 0.5 in toluene was collected, and removal of solvents *in vacuo* gave ^{4}He@**5** as a brown powder (0.331 g, 84%.).

50% ^{4}He filling of ^{4}He@**5** was calculated from the ^{13}C NMR spectrum, and the same filling of ^{4}He@C_{60} was obtained from ring-closure of this sample (section S1.5.5).

^{1}H NMR (500 MHz, $CDCl_3$) δ = 7.68 (t, *J* = 7.8 Hz, 1H), 7.65 (t, *J* = 7.8 Hz, 1H), 7.51 (dd, *J* = 7.8, 0.8 Hz, 1H), 7.39 (dd, *J* = 7.8, 0.8 Hz, 1H), 7.24 (dd, *J* = 7.9, 0.9 Hz, 2 × 1H separated by 0.002 ppm), 7.22 (d, *J* = 10.0 Hz, 0.5H ^{4}He filled), 7.22 (d, *J* = 10.0 Hz, 0.5H empty), 7.17 (d, *J* = 10.0 Hz, 1H), 1.26 (s, 9H), 1.18 (s, 9H) ppm.

^{13}C NMR (126 MHz, $CDCl_3$) δ = 200.408, 192.324, 169.201, 168.842, 165.416, 162.204, 154.819, 150.122, 148.860, 147.961, 147.941, 147.790, 147.461, 146.992, 146.670, 146.634, 146.572, 146.375, 146.177, 146.165, 146.142, 146.018, 145.946, 145.910, 145.798, 145.656, 145.469, 145.177, 145.147, 144.776, 144.744, 144.377, 144.337, 144.061, 143.054, 142.747, 142.578, 142.294, 142.082, 141.977, 141.782, 141.103, 140.744, 140.746, 140.591, 140.265, 140.176, 139.961, 139.682, 139.597, 139.500, 138.792, 138.538, 138.491, 137.493, 137.445, 137.206, 136.498, 136.314, 135.933, 134.965, 133.889, 132.979, 132.913, 132.659, 132.513, 131.677, 129.836, 120.093, 119.689, 117.698, 116.914, 61.097, 54.916, 37.930, 37.836, 30.085, 30.067 ppm. The peaks arising from empty open-fullerene **5** are present but not reported.

HRMS (ESI+) (*m*/*z*): Calcd. for $[^{12}C_{82}H_{27}{}^4HeN_2O_2]^+$ (^{4}He@**5**+H^+) 1075.2093; found 1075.2089.

IR: ν = 2956, 1740, 1567, 1442 cm^{-1}.

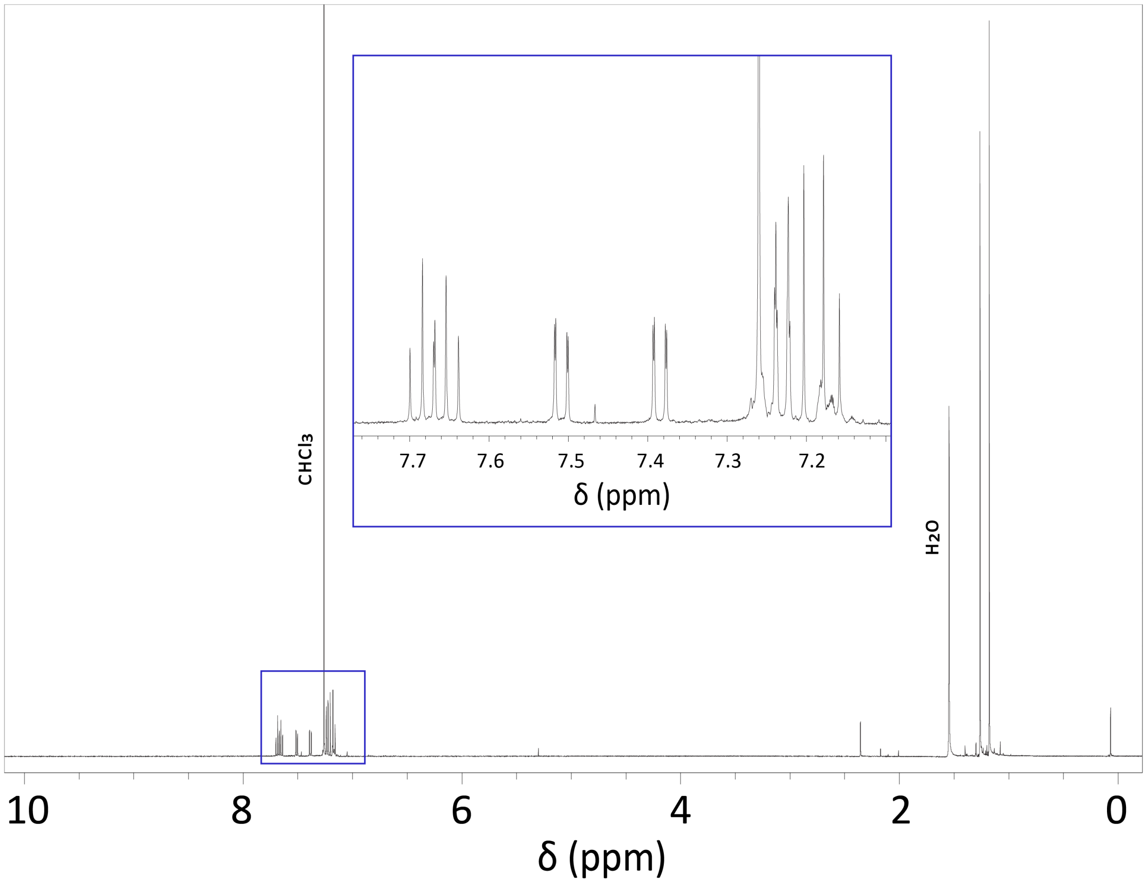

Figure S1.4.4.1 ^{1}H NMR (500 MHz, $CDCl_3$) of ^{4}He@**5** (50% filling).

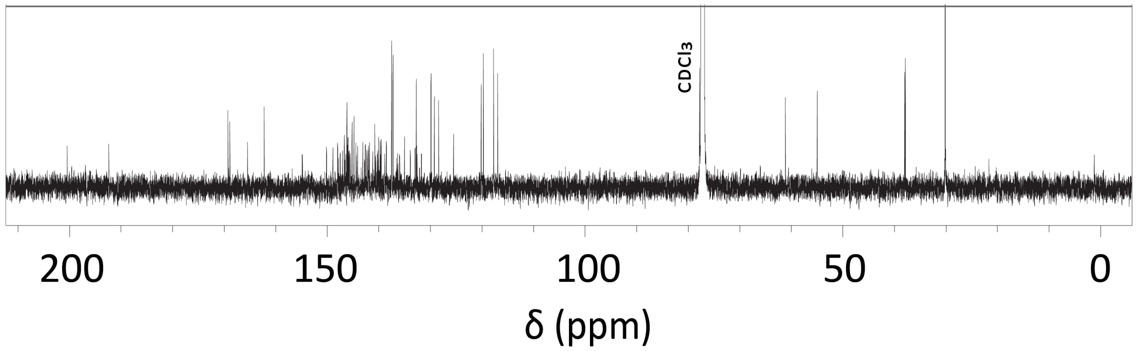

Figure S1.4.4.2 ^{13}C NMR (126 MHz, $CDCl_3$) of ^{4}He@**5** (50% filling).

S1.4.5 Synthesis of ^{3}He@5

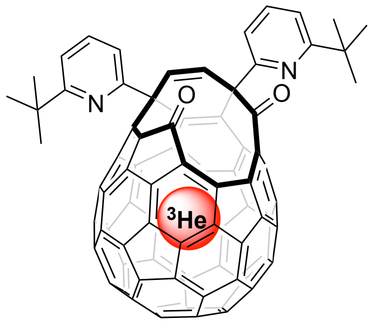

Solid open-fullerene **4** (1.685 g, 1.248 mmol) was packed directly into a steel high-pressure reactor equipped with pressure intensifier (section S1.2). The reactor was degassed under dynamic vacuum (approx. 0.2 mm Hg) before cooling to −196 °C using liquid nitrogen and charging with ^{3}He gas to 35 atm. The gas was then compressed in the reactor to 822 atm, before allowing the reactor to warm to room temperature, at which point the pressure reached 1835 atm. The reactor was then heated to 174°C and maintained at this temperature for 45 min, with a stable internal pressure of 2315 atm during this time. After cooling to room temperature the pressure was slowly released back into the source cylinders. The Sitec syringe was used to transfer as much ^{3}He as possible from the reactor back into the source cylinders and the main part of the pressure system. The residual ^{3}He in the reactor was then recovered sequentially into two 1L evacuated cylinders. The solid residue from the reactor was purified by flash column chromatography (SiO_2 eluted with toluene). Material with Rf = 0.5 in toluene was collected, and removal of solvents *in vacuo* gave ^{3}He@**5** as a brown powder (1.065 g, 79%).

52% ^{3}He filling of ^{3}He@**5** is based on the same filling of the ^{3}He@C_{60} material obtained from ring-closure of this sample (section S1.5.6). No spectroscopic data were collected for 52% filled ^{3}He@**5**, as a sample of 44% filled ^{3}He@**5** had already been characterised. This data is reported below.

^{1}H NMR (500 MHz, $CDCl_3$) δ = 7.68 (t, J = 7.8 Hz, 1H), 7.65 (t, J = 7.8 Hz, 1H), 7.51 (dd, J = 7.8, 0.8 Hz, 1H), 7.39 (dd, J = 7.8, 0.8 Hz, 1H), 7.24 (dd, J = 7.9, 0.9 Hz, 2 × 1H separated by 0.002 ppm), 7.21 (d, J = 10.0 Hz, 1H), 7.17 (d, J = 10.0 Hz, 1H) 1.26 (s, 9H), 1.18 (s, 9H) ppm.

^{13}C NMR (126 MHz, $CDCl_3$) δ = 200.411, 192.322, 169.199, 168.850, 165.420, 162.206, 154.822, 150.118, 148.857, 147.963, 147.937, 147.782, 147.458, 146.988, 146.662, 146.636, 146.568, 146.375, 146.178, 146.165, 146.139, 146.014, 145.944, 145.908, 145.796, 145.652, 145.471, 145.170, 145.146, 144.768, 144.745, 144.375, 144.334, 144.062, 143.051, 142.744, 142.580, 142.291, 142.082, 141.973, 141.782, 141.102, 140.740, 140.741, 140.589, 140.263, 140.172, 139.960, 139.702, 139.597, 139.501, 138.822, 138.541, 138.480, 137.492, 137.446, 137.208, 136.492, 136.312, 135.931, 134.971, 133.889, 132.977, 132.912, 132.662, 132.575, 131.674, 129.836, 120.094, 119.690, 117.698, 116.913, 61.096, 54.915, 37.929, 37.835, 30.085, 30.068 ppm.

The peaks arising from empty open-fullerene **5** are present but not reported.

HRMS (ESI+) (m/z): Calcd. for $[^{12}C_{82}H_{27}{}^{3}HeN_2O_2]^+$ (^{3}He@**5**+H^+) 1074.2227; found 1074.2214.

IR: ν = 2955, 1740, 1696, 1567, 1442 cm^{-1}.

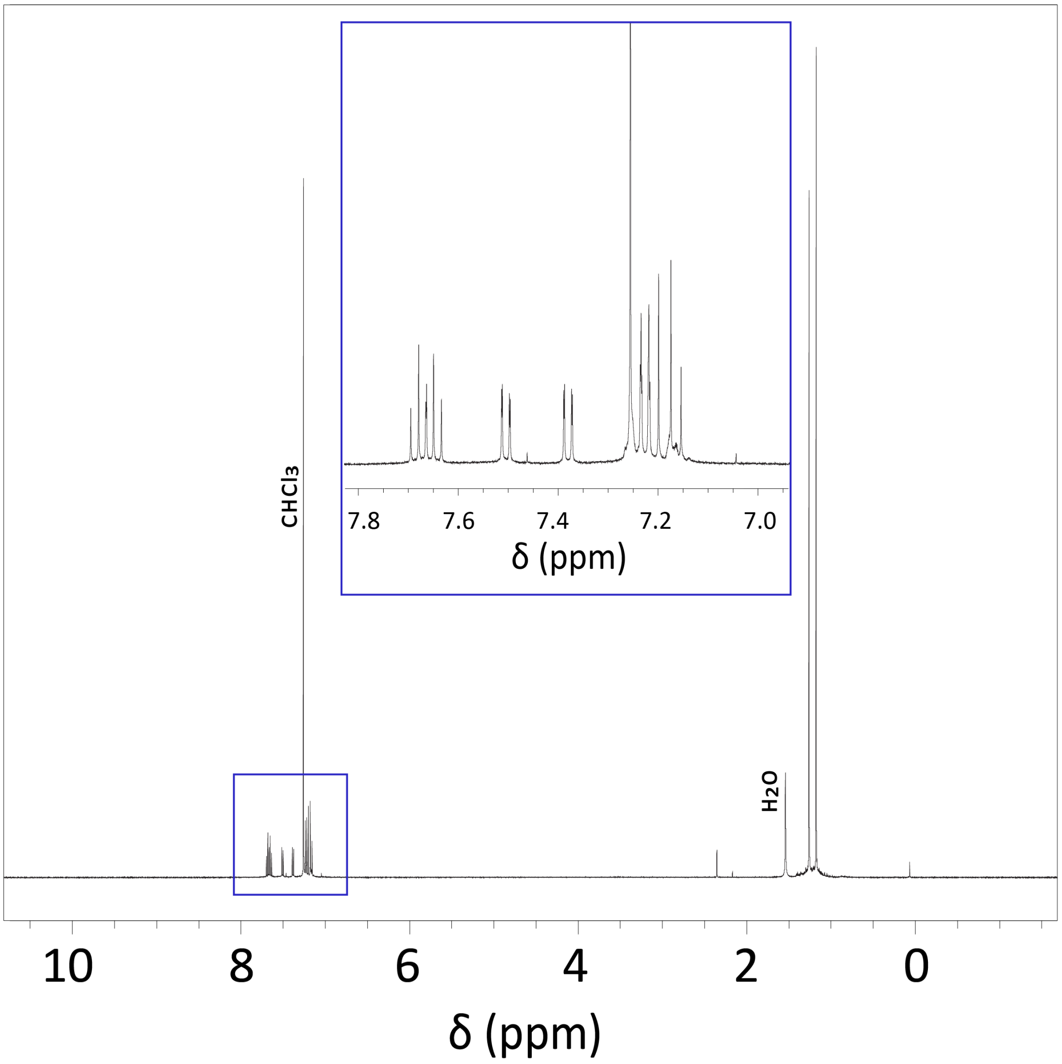

Figure S1.4.5.1 ^{1}H NMR (500 MHz, $CDCl_3$) of ^{3}He@**5** (44% filling).

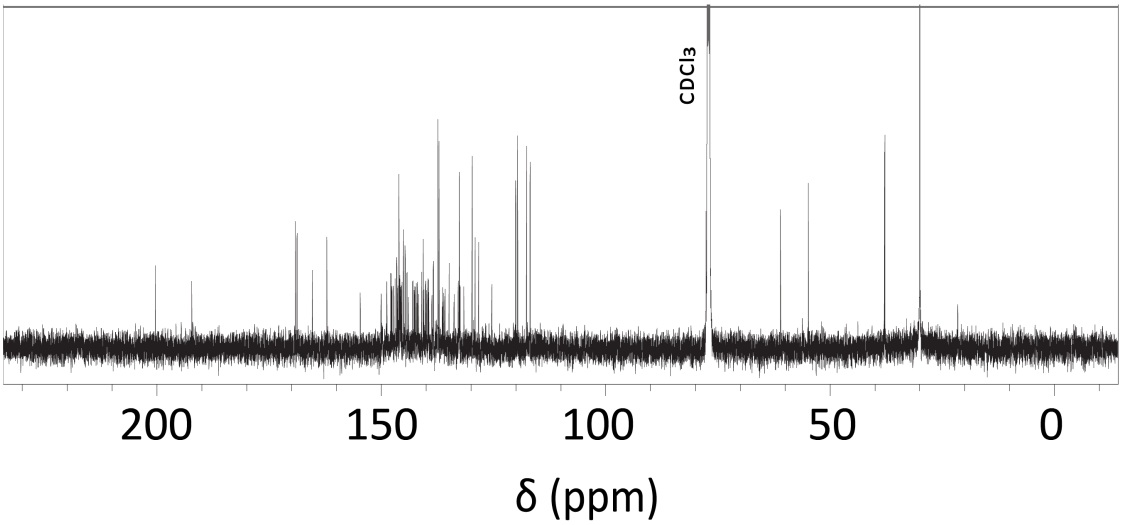

Figure S1.4.5.2 ^{13}C NMR (126 MHz, $CDCl_3$) of ^{3}He@**5** (44% filling).

S1.4.6 Synthesis of Ne@5

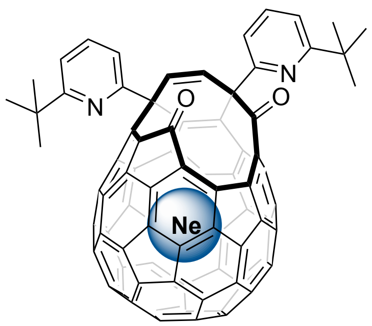

A thin-walled pyrex tube was charged with solid open-fullerene **4** (0.588 g, 0.436 mmol) and inserted into a steel high-pressure reactor equipped with pressure intensifier (section S1.2). The reactor was degassed under dynamic vacuum (approx. 0.2 mm Hg) and then charged with Ne gas to 37 atm. The gas was compressed in the reactor to 346 atm, and the reactor was then cooled to −196 °C using liquid nitrogen. Following further compression to 338 atm, the reactor was warmed to room temperature, at which point the pressure reached 1301 atm. The reactor was then heated to 180 °C and maintained at this temperature for 1.5 h, with a stable internal pressure of 1742 atm during this time. After cooling to room temperature and slow release of the pressure, the solid residue was purified by flash column chromatography (SiO_2 eluted with toluene). Material with Rf = 0.5 in toluene was collected and removal of solvents *in vacuo* gave Ne@**5** as a brown powder (0.391 g, 82%).

63% Ne filling of Ne@**5** was calculated from the ^{13}C NMR spectrum, and the same filling of Ne@C_{60} was obtained from ring-closure of this sample (section S1.5.7).

^{1}H NMR (400 MHz, $CDCl_3$) δ = 7.68 (t, J = 7.8 Hz, 1H), 7.65 (t, J = 7.8 Hz, 1H), 7.51 (dd, J = 7.8, 0.8 Hz, 1H), 7.39 (dd, J = 7.8, 0.8 Hz, 1H), 7.24 (dd, J = 7.9, 0.9 Hz, 2 × 1H separated by 0.002 ppm), 7.21 (d, J = 10.0 Hz, 1H), 7.17 (d, J = 10.0 Hz, 1H) 1.26 (s, 9H), 1.18 (s, 9H) ppm.

^{13}C NMR (126 MHz, $CDCl_3$) δ = 200.408, 192.324, 169.200, 168.847, 165.418, 162.206, 154.847, 150.135, 148.882, 147.961, 147.949, 147.779, 147.474, 146.974, 146.694, 146.650, 146.588, 146.360, 146.178, 146.160, 146.125, 145.996, 145.925, 145.898, 145.776, 145.640, 145.485, 145.149, 145.111, 144.760, 144.750, 144.345, 144.338, 144.076, 142.995, 142.762, 142.602, 142.330, 142.082, 142.009, 141.825, 141.069, 140.735, 140.709, 140.625, 140.332, 140.238, 139.932, 139.673, 139.615, 139.508, 138.839, 138.510, 138.447, 137.495, 137.446, 137.208, 136.563, 136.359, 135.940, 134.950, 133.895, 133.025, 132.922, 132.665, 132.620, 131.636, 129.843, 120.100, 119.691, 117.698, 116.913, 61.100, 54.913, 37.929, 37.836, 30.085, 30.069 ppm. The peaks arising from empty open-fullerene **5** are present but not reported.

HRMS (ESI+) (m/z): Calcd. for $[^{12}C_{82}H_{27}N_2{}^{20}NeO_2]^+$ (Ne@**5**+H^+) 1091.1991; found 1091.1985.

IR: ν = 2955, 1737, 1696, 1566, 1442 cm^{-1}.

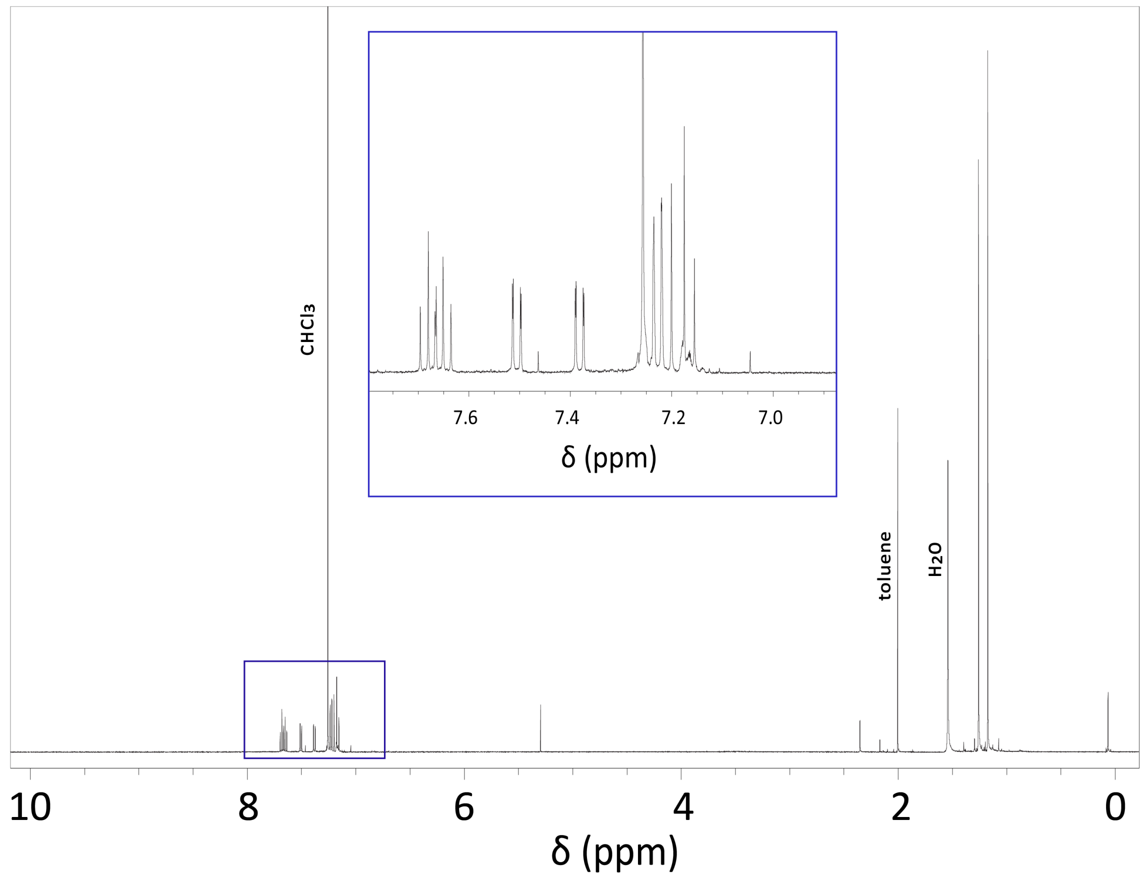

Figure S1.4.6.1 ^{1}H NMR (400 MHz, $CDCl_3$) of Ne@**5** (63% filling).

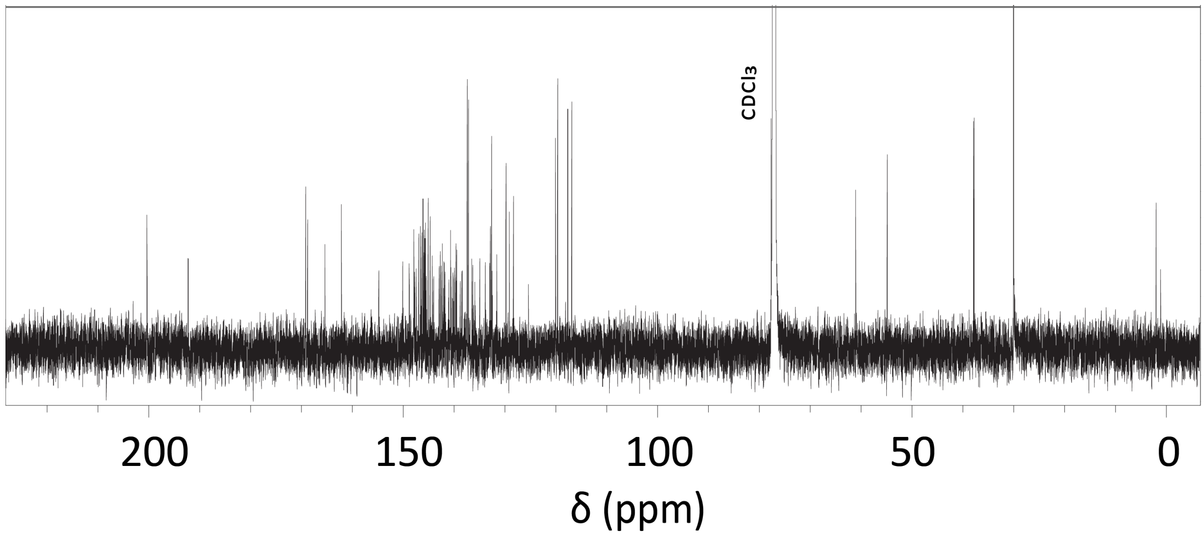

Figure S1.4.6.2 ^{13}C NMR (126 MHz, $CDCl_3$) of Ne@**5** (63% filling).

S1.5 Cage closure of A@5: preparation of endohedral fullerenes A@C_{60}

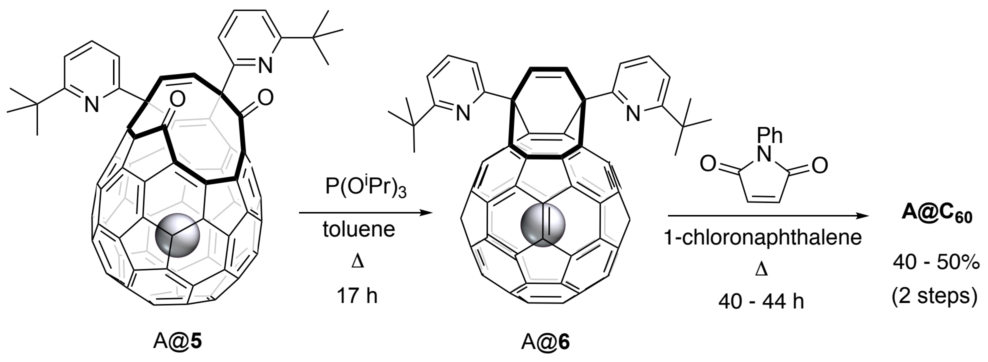

S1.5.1 General procedure

Two-step cage closure of open fullerenes A@**5** was carried out by modification of our previously reported procedure for the synthesis of H_2O@C_{60} and H_2@C_{60}.[4]

To a stirred solution of A@**5** (9 mM solution in toluene) was added triisopropyl phosphite (16 molar equiv.) under N_2 atmosphere. The resulting mixture was stirred at reflux for 17 h, before cooling to room temperature. The mixture was concentrated *in vacuo* and purified by column chromatography (SiO_2 eluted with toluene). The fractions containing material with Rf = 0.95 were collected and evaporated to near dryness to afford A@**6** (toluene-damp) which was taken into 1-chloronaphthalene (60 mL) and transferred directly to a Schlenk tube fitted with high-vacuum valve, containing N-phenylmaleimide (2 molar equiv. based on an expected yield of 90% for the first step). The solution was degassed under dynamic vacuum (<0.2 mm Hg) and placed under N_2 atmosphere. The tube was sealed, and the solution was stirred at 260 °C (using an aluminium heating block) for 40 – 44 h, then cooled to room temperature and poured directly over a silica column packed with toluene. The column was eluted with toluene to collect a purple band, which was concentrated *in vacuo* to remove toluene, before evaporation to dryness by vacuum distillation. The residual solid was washed with Et_2O and dried under a stream of N_2, to obtain crude A@C_{60} as a black solid.

Purification by preparative HPLC (Cosmosil™ Buckyprep 20 mm i.d. × 250 mm column, eluted with toluene at 10 mL min^{-1}) gave the title compound A@C_{60}.

S1.5.2 Synthesis of HD@C_{60}

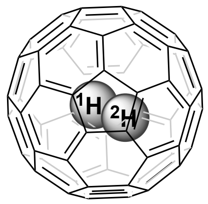

Using the general procedure described in section S1.5.1, cage closure of HD@**5** (0.313 g, 0.291 mmol, 83% filled) was carried out to obtain HD@C_{60} with 83% filling as a black solid (108 mg, 51%).

^{1}H NMR (500 MHz, 1,2-dichlorobenzene-*d*$_4$) δ = −1.49 (1:1:1 t, J = 41.6 Hz, 1H, endohedral HD) ppm. The ^{1}H NMR spectrum also showed 4% endohedral H_2 (δ = −1.46 ppm, s, 0.08H) that results from disproportionation of HD in contact with the steel pressure reactor during the filling procedure to prepare HD@**5**. See figure S1.5.2.1 below.

^{13}C NMR (126 MHz, 1,2-dichlorobenzene-*d*$_4$) δ = 142.879 (HD@C_{60}) 142.803 (C_{60}) ppm.

HRMS-APPI (*m*/*z*): Calcd. for $[^{12}C_{60}{}^{1}H^{2}H]^{+\bullet}$ 723.0214; found 723.0218. Calcd. for $[^{12}C_{59}{}^{13}C_{1}{}^{1}H^{2}H]^{+\bullet}$ 724.0247; found 724.0250. Calcd. for $[^{12}C_{60}]^{+\bullet}$ 719.9995; found 719.9996. Calcd. for $[^{12}C_{59}{}^{13}C_{1}]^{+\bullet}$ 721.0028; found 721.0031.

IR: ν = 1427, 1180, 574 cm^{-1}.

UV (toluene): λ_{max} 335.7 nm, ε 35000 M^{-1}cm^{-1}.

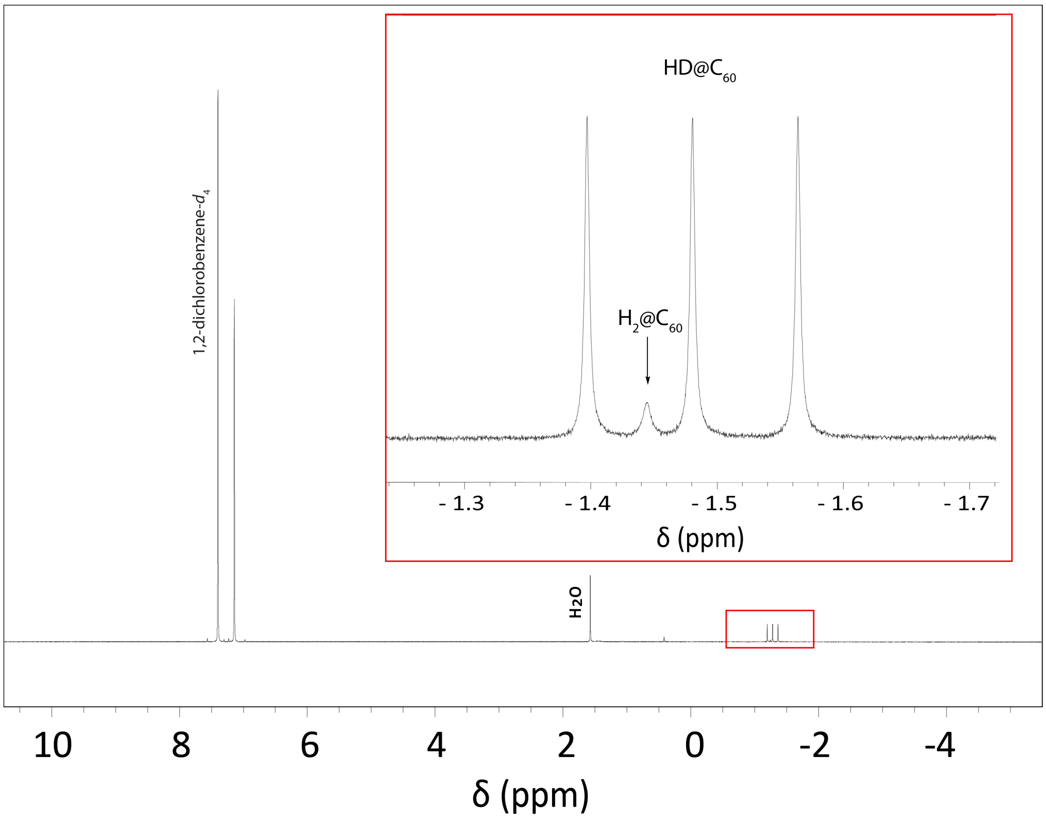

Figure S1.5.2.1 ^{1}H NMR (500 MHz, 1,2-dichlorobenzene-*d*$_4$) of HD@C_{60} (83% filling).

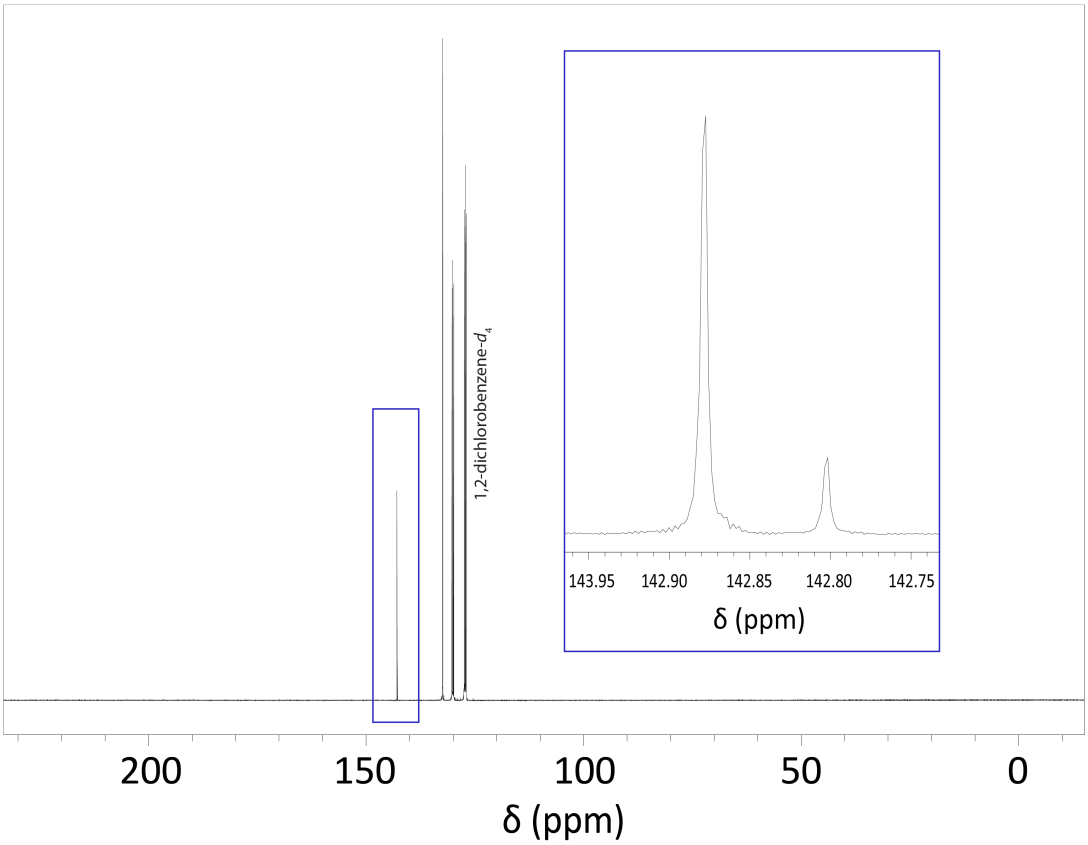

Figure S1.5.2.2 ^{13}C NMR (126 MHz, 1,2-dichlorobenzene-*d*$_4$) of HD@C_{60} (83% filling).

S1.5.3 Synthesis of D_2@C_{60}

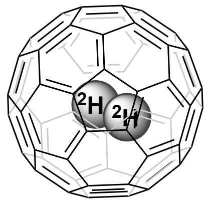

Using the general procedure described in section S1.5.1, cage closure of D_2@**5** (0.352 g, 0.327 mmol, 73% filled) was carried out to obtain D_2@C_{60} with 73% filling as a black solid (116 mg, 49%).

^{13}C NMR (126 MHz, 1,2-dichlorobenzene-*d*$_4$) δ =142.877 (D_2@C_{60}) 142.802 (C_{60}) ppm.

^{2}H NMR (77 MHz, 1,2-dichlorobenzene-*d*$_4$) $\delta = -1.54$ ppm. The ^{2}H peak is referenced to 1,2-dichlorobenzene-*d*$_4$.[5]

HRMS-APPI (*m*/*z*): Calcd. for $[^{12}C_{60}{}^{2}H_2]^{+\bullet}$ 724.0277; found 724.0276. Calcd. for $[^{12}C_{59}{}^{13}C_1{}^{2}H_2]^{+\bullet}$ 725.0310; found 724.0312. Calcd. for $[^{12}C_{60}]^{+\bullet}$ 719.9995; found 719.9994. Calcd. for $[^{12}C_{59}{}^{13}C_1]^{+\bullet}$ 721.0028; found 721.0027.

IR: v = 1426, 1180, 573 cm^{-1}.

UV (toluene): λ_{max} 335.3 nm, ε 42000 $M^{-1}cm^{-1}$.

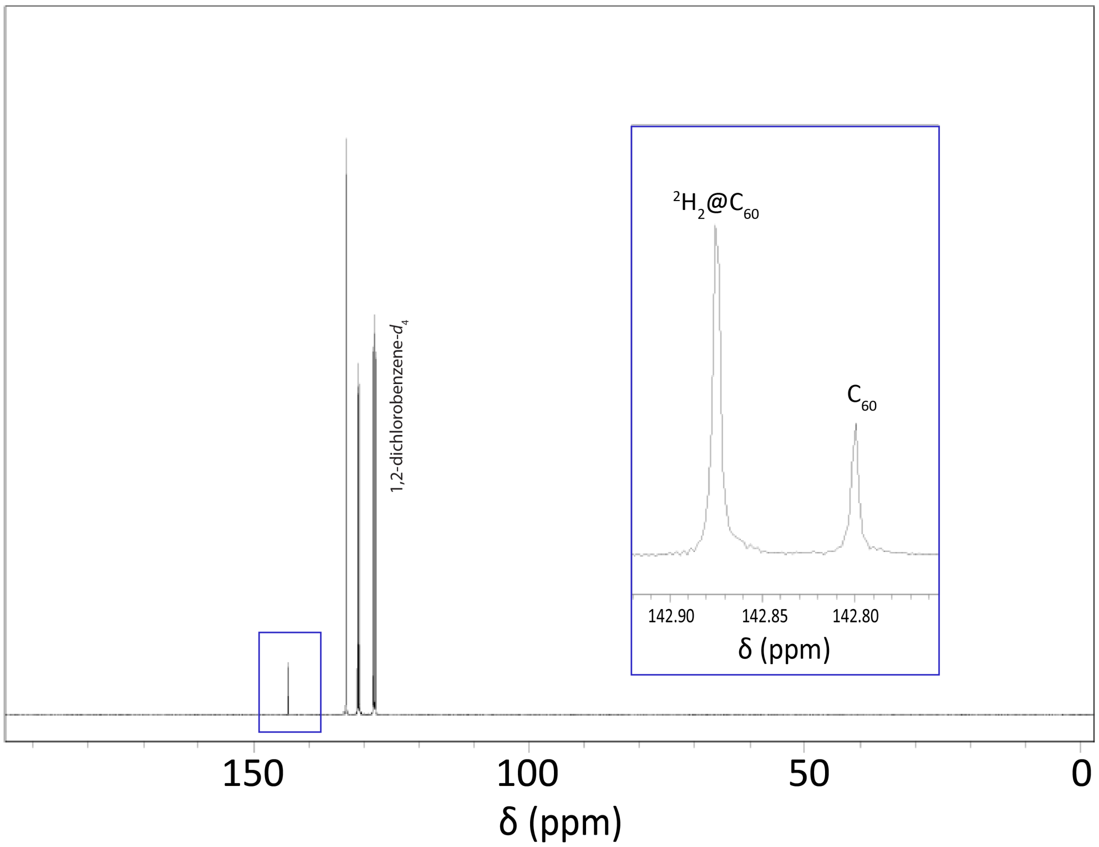

Figure S1.5.3.1 ^{13}C NMR (126 MHz, 1,2-dichlorobenzene-*d*4) of D_2@C_{60} (73% filling).

S1.5.4 Synthesis of H_2@C_{60}

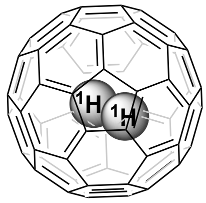

Using the general procedure described in section S1.5.1, cage closure of H_2@**5** (0.351 g, 0.327 mmol, >95% filled) was carried out to obtain H_2@C_{60} with approx. 95% filling as a black solid (122 mg, 52%).

^1H NMR (500 MHz, 1,2-dichlorobenzene-*d*4) $\delta = -1.46$ ppm

^{13}C NMR (126 MHz, 1,2-dichlorobenzene-*d*4) δ = 142.880 (H_2@C_{60}) 142.803 (C_{60}) ppm.

All spectroscopic data were consistent with those previously reported for H_2@C_{60}.[6]

S1.5.5 Synthesis of $^4He@C_{60}$

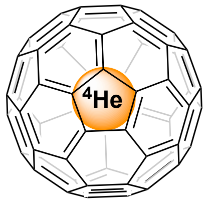

Using the general procedure described in section S1.5.1, cage closure of $^4He@$**5** (0.419 g, 0.390 mmol, 50% filled) was carried out to obtain $^4He@C_{60}$ with 50% filling as a black solid (142 mg, 50%).

^{13}C NMR (176 MHz, 1,2-dichlorobenzene-*d*4) δ = 142.827 ($^4He@C_{60}$) 142.803 (C_{60}) ppm.

HRMS-APPI (*m*/*z*): Calcd. for $[^{12}C_{60}{}^4He]^{+\bullet}$ 724.0021; found 724.0027. Calcd. for $[^{12}C_{59}{}^{13}C_1{}^4He]^{+\bullet}$ 725.0054; found 725.0059. Calcd. for $[^{12}C_{58}{}^{13}C_2{}^4He]^{+\bullet}$ 726.0088; found 726.0091. Calcd. for $[^{12}C_{60}]^{+\bullet}$ 719.9995; found 719.9989. Calcd. for $[^{12}C_{59}{}^{13}C_1]^{+\bullet}$ 721.0028; found 721.0026. Calcd. for $[^{12}C_{58}{}^{13}C_2]^{+\bullet}$ 722.0062; found 722.0065.

IR: ν = 1427, 1180, 574 cm^{-1}.

UV (toluene): λ_{max} 335.7 nm, ε 37000 $M^{-1}cm^{-1}$.

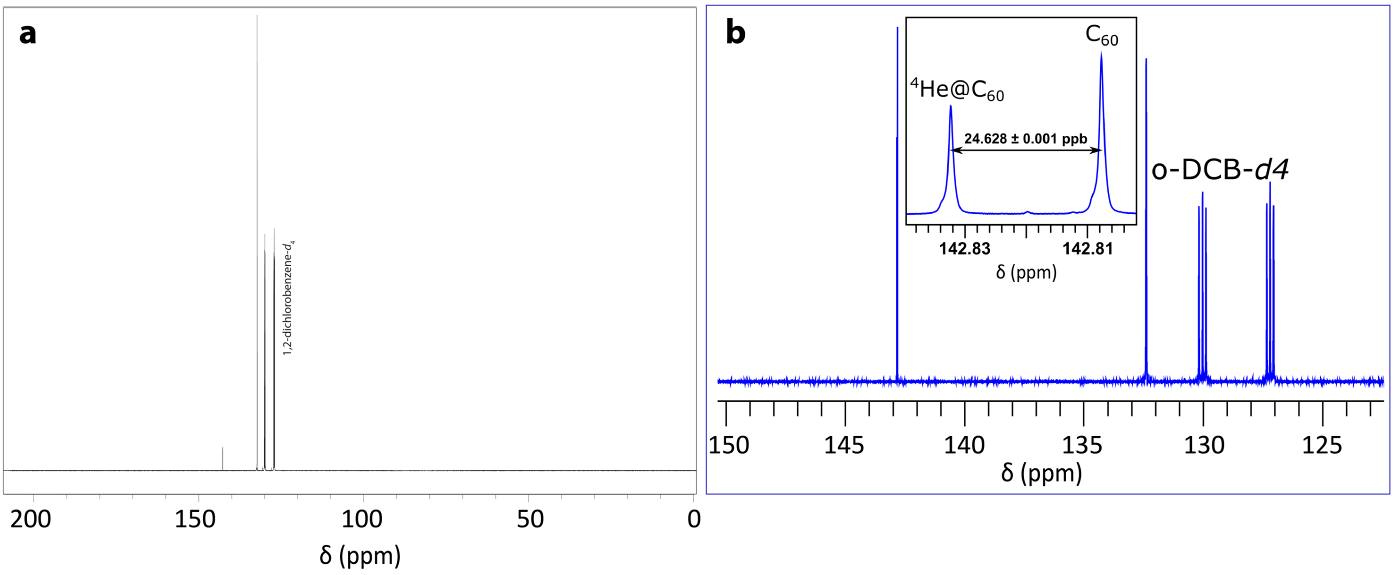

Figure S1.5.5.1 (a) ^{13}C NMR (126 MHz, 1,2-dichlorobenzene-*d*4) of $^4He@C_{60}$ (50% filling). (b) ^{13}C NMR spectrum of a mixed sample of $^4He@C_{60}$ and C_{60} corresponding to 41% ^4He filling, approx. 30 mM solution in degassed 1,2-dichlorobenzene-*d*4 at a field of 176 MHz and 298 K, acquired with 128 transients.

S1.5.6 Synthesis of $^{3}He@C_{60}$

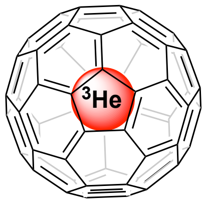

Two-step cage closure of open fullerene ^{3}He@**5** was carried out according to our previously reported procedure.[4] The optimized general procedure given in section S1.5.1 was developed later, but we did not apply this method to a repeated synthesis of $^{3}He@C_{60}$ due to the expense and limited availability of ^{3}He gas.

To a stirred solution of 52% filled ^{3}He@**5** (2.027g, 1.887 mmol) was added triisopropyl phosphite (7.50 mL, 30.4 mmol) under N_2 atmosphere. The resulting mixture was stirred at reflux for 16 h, before cooling to room temperature. The mixture was concentrated *in vacuo* and purified by column chromatography (SiO_2 eluted with toluene). The fractions containing material with Rf = 0.95 were collected and evaporated to near dryness to afford ^{3}He@**6** (toluene-damp) which was taken into 1-chloronaphthalene (190 mL) and transferred in equal proportions to four reaction flasks containing *N*-phenylmaleimide (4 × 290 mg, 6.70 mmol). The solution was degassed under dynamic vacuum (<0.2 mm Hg), placed under N_2 atmosphere, and stirred at 245 °C for 40 h before cooling to room temperature and poured directly over a silica column packed with toluene. The column was eluted with toluene to collect a purple band, which was concentrated *in vacuo* to remove toluene, before evaporation to dryness by vacuum distillation to obtain crude $^{3}He@C_{60}$ as a black solid.

Purification was carried out by preparative HPLC (2 × Cosmosil™ Buckyprep 20 mm i.d. × 250 mm columns in series, eluted with toluene at 10 mL min^{-1}). After 3 cycles and a retention time of 92-93 min, $^{3}He@C_{60}$ was obtained with 52% filling as a black solid (573 mg, 42%).

^{13}C NMR (176 MHz, 1,2-dichlorobenzene-d_4) δ = 142.828 ($^{3}He@C_{60}$) 142.803 (C_{60}) ppm.

HRMS-APPI (*m*/*z*): Calcd. for $[^{12}C_{60}{}^{3}He]^{+\bullet}$ 723.0155; found 723.0151. Calcd. for $[^{12}C_{59}{}^{13}C_1{}^{3}He]^{+\bullet}$ 724.0188; found 724.0193. Calcd. for $[^{12}C_{58}{}^{13}C_2{}^{3}He]^{+\bullet}$ 725.0222; found 725.0231. Calcd. for $[^{12}C_{60}]^{+\bullet}$ 719.9995; found 719.9979. Calcd. for $[^{12}C_{59}{}^{13}C_1]^{+\bullet}$ 721.0028; found 721.0016. Calcd. for $[^{12}C_{58}{}^{13}C_2]^{+\bullet}$ 722.0062; found 722.0069.

IR: ν = 1427, 1180, 574 cm^{-1}.

UV (toluene): λ_{max} 335.7 nm, ε 39000 M^{-1}cm^{-1}.

The ^{3}He NMR spectrum of ~0.1% filled $^{3}He@C_{60}$ has been reported by Saunders *et al.*[7]

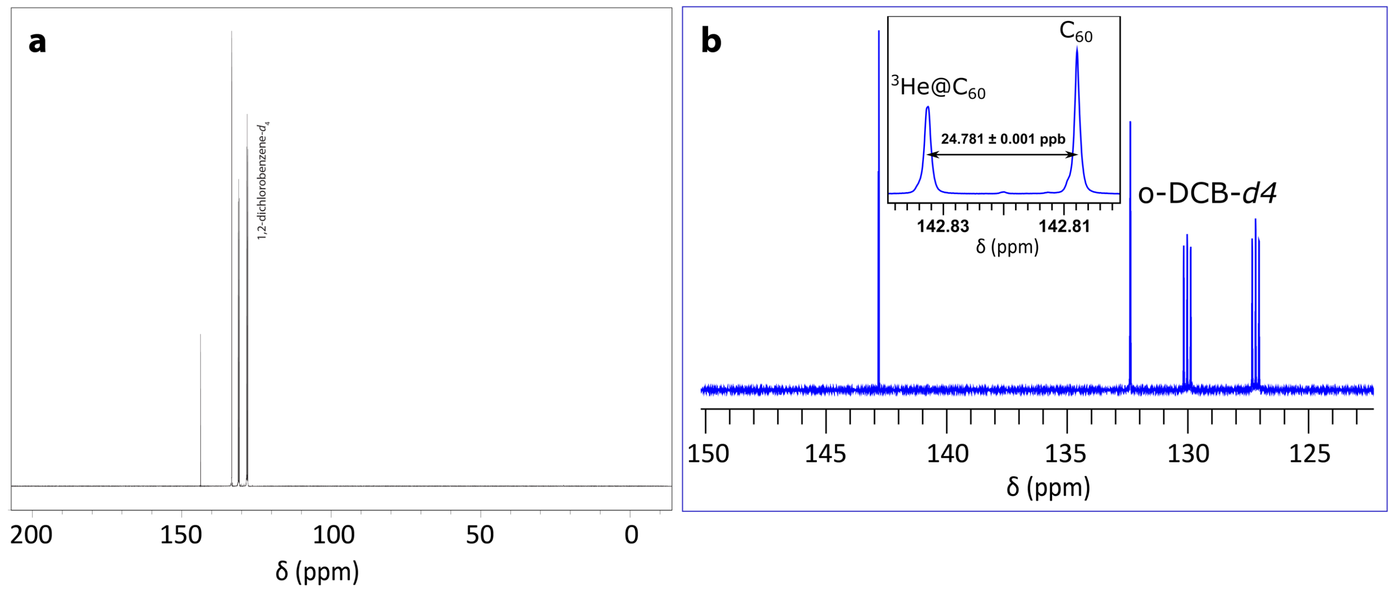

Figure S1.5.6.1 (a) ^{13}C NMR (126 MHz, 1,2-dichlorobenzene-*d*$_4$) of $^3He@C_{60}$ (52% filling). (b) ^{13}C NMR spectrum of a mixed sample of $^3He@C_{60}$ and C_{60} corresponding to 44% ^3He filling, approx. 30 mM solution in degassed 1,2-dichlorobenzene-*d*$_4$ at a field of 176 MHz and 298 K, acquired with 424 transients.

S1.5.7 Synthesis of Ne@C_{60}

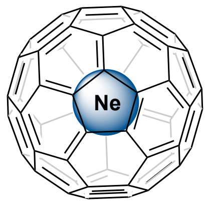

Using the general procedure described in section S1.5.1, cage closure of Ne@**5** (0.391 g, 0.358 mmol, 63% filled) was carried out to obtain Ne@C_{60} with 63% filling as a black solid (115 mg, 43%).

Enrichment of 63% filled Ne@C_{60}, to a sample with >99.5% neon encapsulation, was carried out by recycling preparative HPLC (2 × Cosmosil™ Buckyprep 20 mm i.d. × 250 mm columns in series, eluted with toluene at 10 mL min^{-1}). After 20 cycles, empty C_{60} was collected with an overall retention time of 626 min, and Ne@C_{60} with a retention time of 631 min (figure S1.5.7.1).

^{13}C NMR (176 MHz, 1,2-dichlorobenzene-*d*$_4$) δ =142.827 (Ne@C_{60}) ppm.

HRMS-APPI (*m*/*z*): Calcd. for $[^{12}C_{60}Ne]^{+\bullet}$ 739.9919; found 739.9927. Calcd. for $[^{12}C_{59}{}^{13}C_1Ne]^{+\bullet}$ 740.9952; found 740.9969.

IR: ν = 1427, 1180, 574 cm^{-1}.

UV (toluene): λ_{max} 335.3 nm, ε 40000 M^{-1}cm^{-1}.

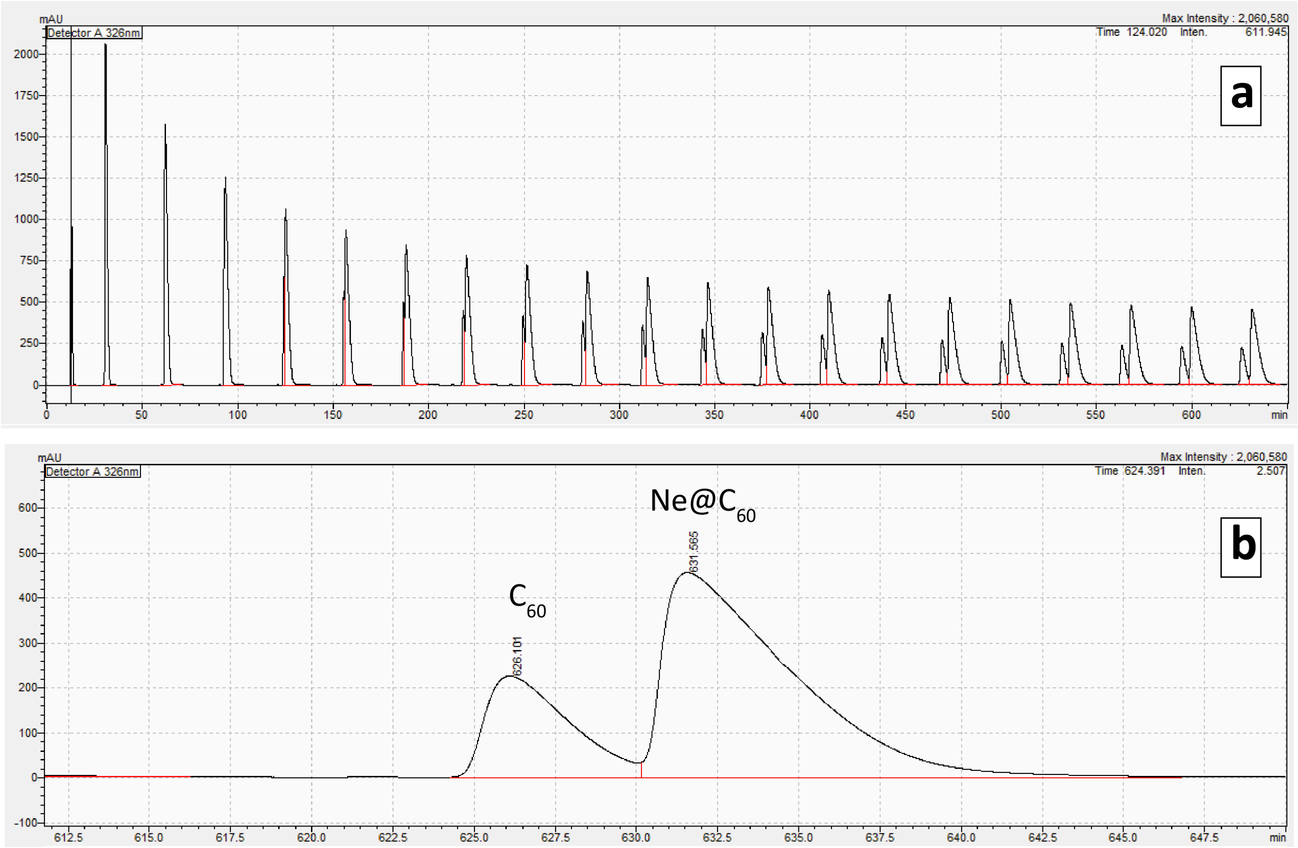

Figure S1.5.7.1 (a) Recycling HPLC chromatogram for the isolation of Ne@C_{60}. Conditions: 2 × Cosmosil™ Buckyprep 20 mm i.d. × 250 mm columns in series, eluted with toluene at 10 mL min^{-1}. (b) Expansion of the 20th cycle.

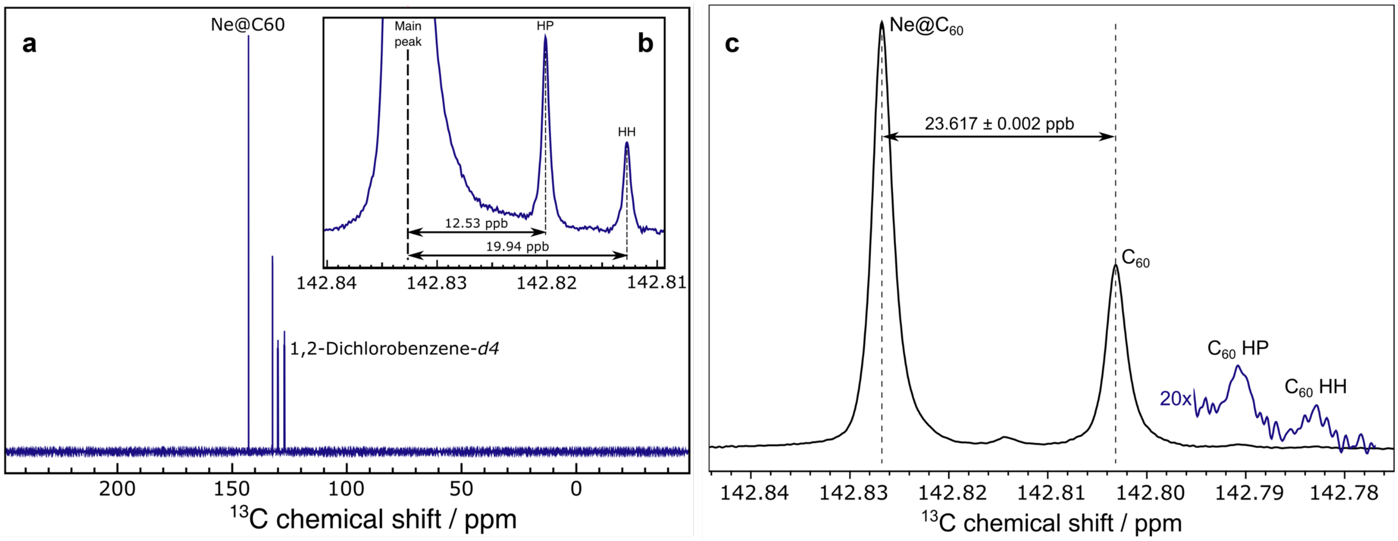

Figure S1.5.7.2 Panels (a) and (b) are reproduced from Figure 4 of the main paper. (a) ^{13}C NMR spectrum of Ne@C_{60} (>99.5% neon filling), 31 – 32 mM solution in degassed 1,2-dichlorobenzene-d_4 at a field of 176 MHz and 298 K, acquired with 152 transients. There is no visible peak from unfilled C_{60}. (b) Expanded view of the base of the Ne@C_{60} resonance, acquired at 298 K with 680 transients, to show side peaks arising from minor isotopomers with adjacent ^{13}C nuclei that share either a hexagon-pentagon (HP) or hexagon-hexagon (HH) edge. (c) ^{13}C NMR spectrum of a mixed sample of Ne@C_{60} and C_{60} (corresponding to 70% neon filling), 29 mM solution in degassed 1,2-dichlorobenzene-d_4 at a field of 176 MHz and 298 K, acquired with 320 transients. The HP and HH side peaks of C_{60} are indicated in the 20× expansion.

S1.5.8 UV-vis spectra of endohedral fullerenes A@C_{60}

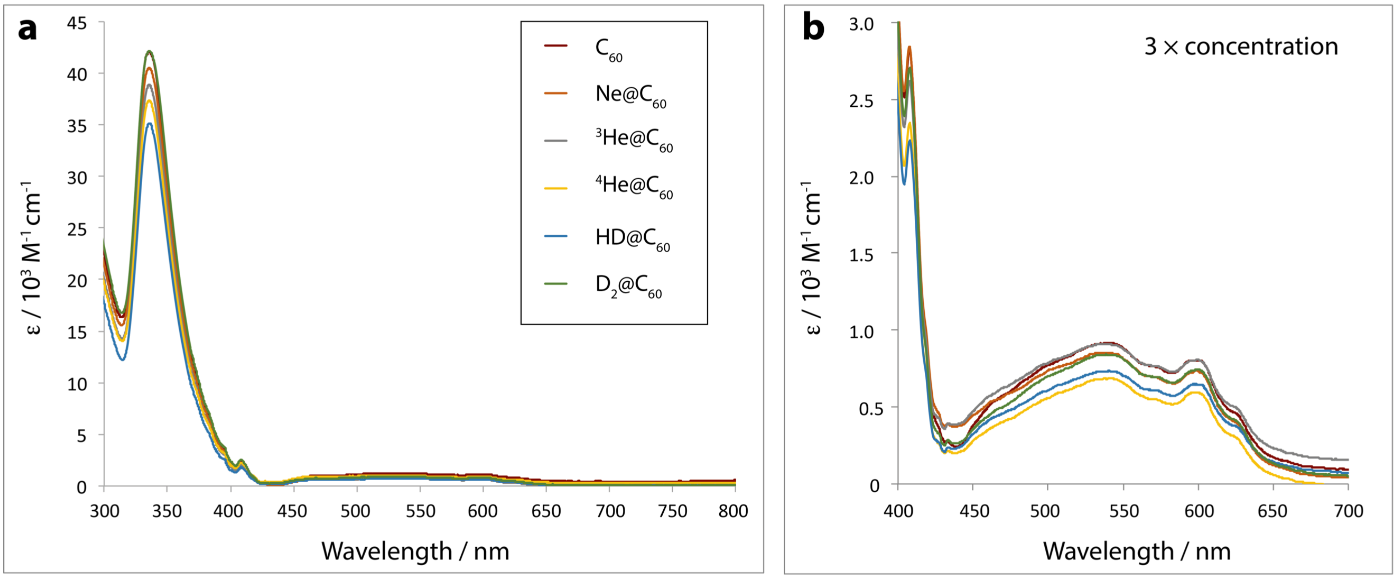

Figure S1.5.8.1 (a) UV-vis spectra of endohedral fullerenes, from serial dilution of a 0.2 mg mL^{-1} solution in toluene (b) UV-vis spectra of endohedral fullerenes in toluene solution (0.2 mg mL^{-1}).

S2. Density Functional Theory Calculations

Model structures **2a** and **4a** were used to represent compounds **2** and **4**, in which the 6-tert-butylpyridyl groups were replaced by methyl substituents. Calculations were carried out using Gaussian 09,[8] using the M06-2X functional[9] with cc-pVDZ[10] basis set to locate minimum energy and transition state structures and to characterise them through frequency calculations. The cc-pVTZ[10] basis set with an ultrafine integration grid was used to calculate electronic energies and to correct for Basis Set Superposition Error using the counterpoise method.[11] Thermal corrections to the electronic energy to give the enthalpy at 298 K and 1 atm were derived from frequency calculations at the M06-2X/cc-pVDZ level using the Gaussian freqchk utility.[12] The frequencies were not scaled and low frequency modes were not removed.

S3. Crystallography data

S3.1 X-ray structure determination of phosphorus ylid 4

S3.1.2 Experimental method

Preparation of a single crystal was made by slow evaporation, after layering a few drops of hexane over a solution of open-fullerene **4** (1 mg) in $CHCl_3$ (1 mL), in a 1.5 mL glass vial. The crystal 0.43×0.19×0.06 mm^3 was mounted on a MITIGEN holder with silicon oil on a Rigaku AFC12 FRE-VHF diffractometer. The crystal was kept at a steady T = 100(2) K during data collection. The structure was solved with the ShelXT[13a] structure solution program using the Intrinsic Phasing solution method and by using Olex2[14] as the graphical interface. The model was refined with version 2016/6 of ShelXL[13b] using Least Squares minimisation.

S3.1.3 Crystal data

$C_{101}Cl_3H_{42}N_2O_3P$, M_r = 1468.68, monoclinic, $P2_1/c$ (No. 14), a = 23.5096(5) Å, b = 13.6771(2) Å, c = 19.8458(4) Å, □ = 95.8152(18)°, $\alpha = \gamma = 90°$, V = 6348.4(2) Å^3, T = 100(2) K, Z = 4, Z' = 1, μ(MoKα) = 0.237, 95342 reflections measured, 16105 unique (R_{int} = 0.0573) which were used in all calculations. The final wR_2 was 0.1490 (all data) and R_1 was 0.0593 (I > 2(I)).

CCDC	1953259	*Z*	4
Formula	$C_{101}Cl_3H_{42}N_2O_3P$	*Z'*	1
	$C_{100}H_{41}N_2O_3P$, $CHCl_3$	Wavelength/Å	0.71073
$D_{calc.}$/ g cm^{-3}	1.537	Radiation type	MoK_α
μ/mm^{-1}	0.237	Θ_{min}/°	1.811
Formula Weight	1468.68	Θ_{max}/°	28.500
Colour	orange	Measured Refl.	95342
Shape	plate	Independent Refl.	16105
Size/mm^3	0.43×0.19×0.06	Reflections with I > 2(I)	12136
T/K	100(2)	R_{int}	0.0573
Crystal System	monoclinic	Parameters	961
Space Group	$P2_1/c$	Restraints	0
a/Å	23.5096(5)	Largest Peak	1.157
b/Å	13.6771(2)	Deepest Hole	-0.668
c/Å	19.8458(4)	GooF	1.029
α/°	90	wR_2 (all data)	0.1490
β/°	95.8152(18)	wR_2	0.1391
γ/°	90	R_1 (all data)	0.0814
V/Å^3	6348.4(2)	R_1	0.0593

S3.1.4 Structure Quality Indicators

**Reflections:**	d min (Mo) 0.74	I/σ 23.2	Rint 5.73%	complete 100% (IUCr) 100%
**Refinement:**	Shift -0.001	Max Peak 1.2	Min Peak -0.7	GooF 1.029

An orange plate-shaped crystal with dimensions 0.43×0.19×0.06 mm^3 was mounted on a MITIGEN holder with silicon oil. X-ray diffraction data were collected using a Rigaku AFC12 FRE-VHF diffractometer equipped with an Oxford Cryosystems low-temperature device, operating at T = 100(2) K.

Data were measured using profile data from □-scans of 0.5 ° per frame for 20.0 s using MoK_α radiation (Rotating-anode X-ray tube, 45.0 kV, 55.0 mA). The total number of runs and images was based on the strategy calculation from the program CrysAlisPro (Rigaku, V1.171.40.37a, 2019). The maximum resolution achieved was Θ = 28.500°.

Cell parameters were retrieved using the CrysAlisPro (Rigaku, V1.171.40.37a, 2019) software and refined using CrysAlisPro (Rigaku, V1.171.40.37a, 2019) on 31805 reflections, 33 % of the observed reflections. Data reduction was performed using the CrysAlisPro (Rigaku, V1.171.40.37a, 2019) software that corrects for Lorentz polarisation. The final completeness is 100.00 % out to 28.500° in Θ.

A multi-scan absorption correction was performed using CrysAlisPro 1.171.40.37a (Rigaku Oxford Diffraction, 2019) using spherical harmonics as implemented in SCALE3 ABSPACK. The absorption coefficient □ of this material is 0.237 mm^{-1} at this wavelength (λ = 0.711Å) and the minimum and maximum transmissions are 0.856 and 1.000

The structure was solved in the space group $P2_1/c$ (# 14) by Intrinsic Phasing using the ShelXT[13a] structure solution

program and refined by Least Squares using version 2016/6 of ShelXL.[13b] All non-hydrogen atoms were refined anisotropically. Hydrogen atom positions were calculated geometrically and refined using the riding model.

_exptl_absorpt_process_details: CrysAlisPro 1.171.40.37a (Rigaku Oxford Diffraction, 2019) using spherical harmonics as implemented in SCALE3 ABSPACK.

There is a single molecule in the asymmetric unit, which is represented by the reported sum formula. In other words: Z is 4 and Z' is 1.

A solvent mask was calculated, and 276.0 electrons were found in a volume of 676.0 Å^3 in 3 voids. This is consistent with the presence of 1[$CHCl_3$] per formula unit that account for 232.0 electrons.

Generated precession images

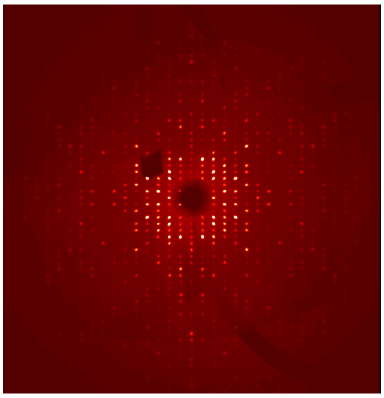

0kl

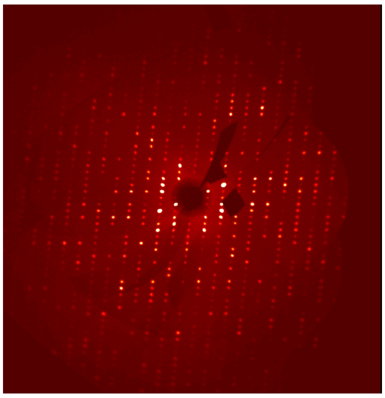

h0l

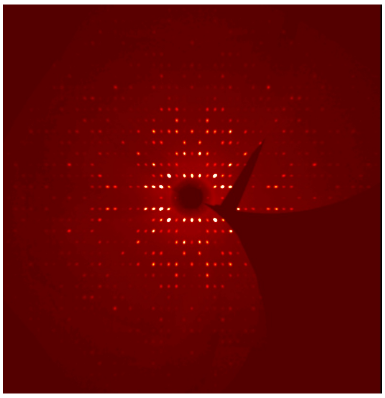

hk0

S3.1.5 Data Plots: Diffraction Data

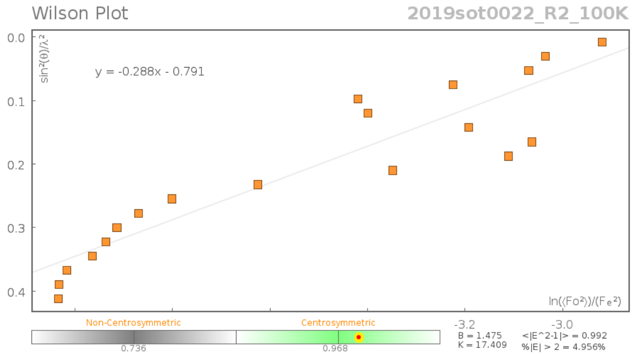

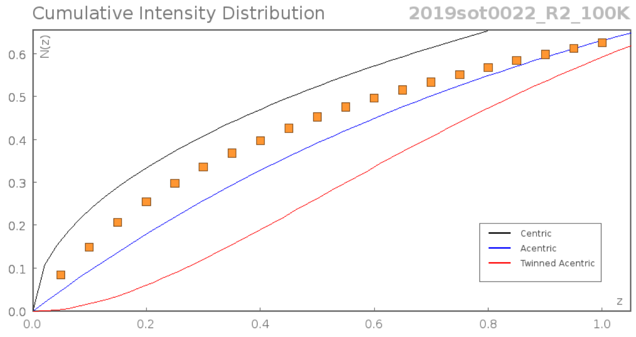

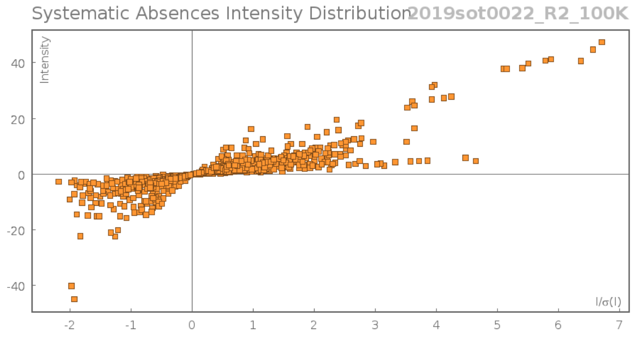

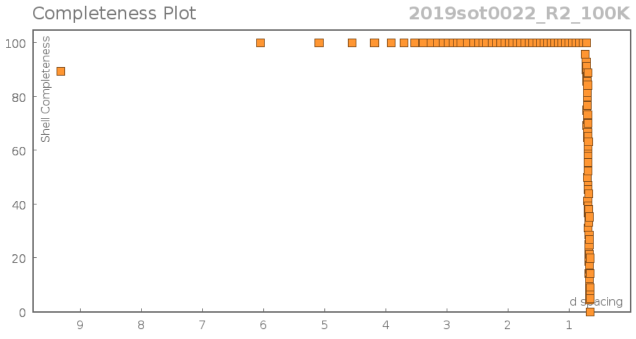

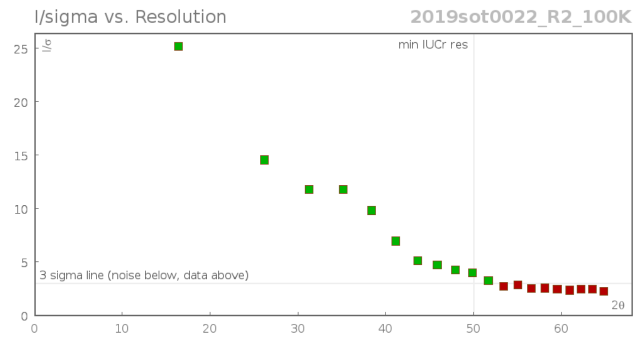

S3.1.6 Data Plots: Refinement and Data

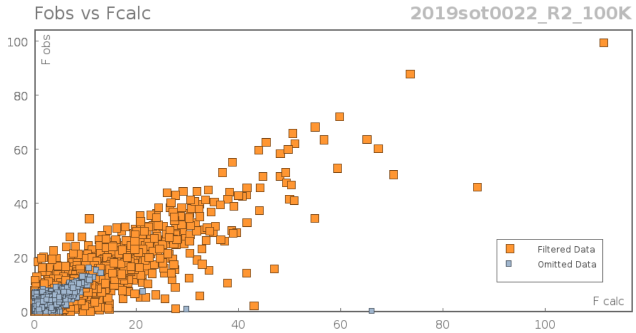

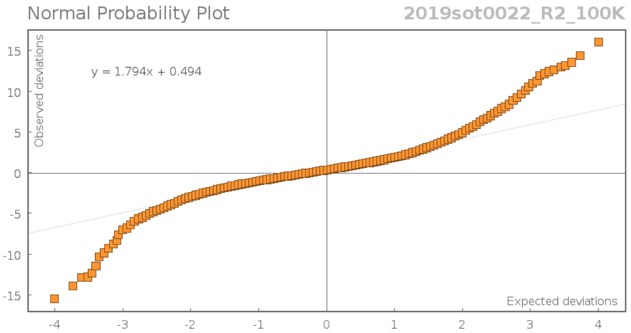

S3.1.7 Reflection Statistics

Total reflections (after filtering)	97287	Unique reflections	16105
Completeness	1.0	Mean I/□	19.47
hkl_{max} collected	(35, 19, 29)	hkl_{min} collected	(-35, -19, -29)
hkl_{max} used	(31, 18, 26)	hkl_{min} used	(-31, 0, 0)
Lim d_{max} collected	100.0	Lim d_{min} collected	0.74
d_{max} used	11.81	d_{min} used	0.74
Friedel pairs	23393	Friedel pairs merged	1
Inconsistent equivalents	32	R_{int}	0.0573
R_{sigma}	0.0432	Intensity transformed	0
Omitted reflections	0	Omitted by user (OMIT hkl)	8
Multiplicity	(30149, 18471, 8649, 2468, 384, 31)	Maximum multiplicity	16
Removed systematic absences	1937	Filtered off (Shel/OMIT)	7729

S3.1.8 Images of the Crystal on the Diffractometer

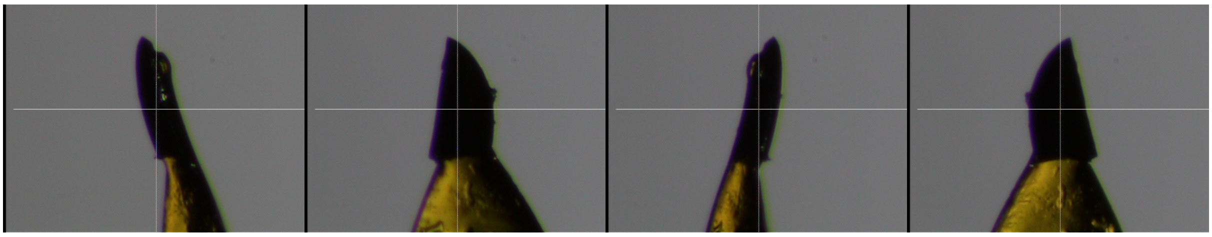

Table S3.1.1 Fractional Atomic Coordinates ($\times10^4$) and Equivalent Isotropic Displacement Parameters (Å$^2\times10^3$) for open fullerene **4**. U_{eq} is defined as 1/3 of the trace of the orthogonalised U_{ij}.

**Atom**	**x**	**y**	**z**	U_{eq}
P1	2355.6(2)	-528.1(4)	5896.5(3)	15.52(11)
O1	2128.0(7)	1404.3(12)	5143.2(8)	23.4(3)
O2	3367.1(6)	550.7(11)	6736.7(8)	20.3(3)
O3	3170.0(7)	2620.3(12)	5180.4(8)	24.2(3)
N1	4181.0(8)	3745.5(14)	8160.5(9)	19.3(4)
N2	3771.1(9)	4750.0(14)	5346.0(10)	23.9(4)
C1	254.6(9)	4989.5(18)	7340.4(13)	26.0(5)
C2	739.8(10)	5509.5(17)	7680.5(12)	24.2(5)
C3	1067.4(10)	5066.3(17)	8218.7(11)	23.0(5)
C4	914.5(10)	4117.5(17)	8459.0(11)	22.9(5)
C5	443.5(10)	3624.6(18)	8137.8(12)	24.6(5)
C6	117.0(9)	4067.1(18)	7561.2(12)	25.6(5)
C7	-33.1(9)	3298.4(18)	7071.8(12)	24.9(5)
C8	-40.4(9)	3490.3(18)	6384.6(12)	25.3(5)
C9	86.9(9)	4460.2(18)	6149.8(12)	26.3(5)
C10	234.3(10)	5197.7(18)	6619.4(13)	26.5(5)
C11	699.6(10)	5854.2(17)	6516.0(13)	25.4(5)
C12	1013.7(10)	6040.0(16)	7171.2(12)	23.6(5)
C13	1604.7(10)	6103.6(16)	7225.5(12)	22.5(5)
C14	1944.4(9)	5615.5(16)	7774.2(11)	21.1(4)
C15	1685.9(9)	5107.4(16)	8257.9(11)	20.6(4)
C16	1920.6(9)	4178.6(16)	8500.3(10)	19.3(4)
C17	1441.3(9)	3588.9(17)	8652.6(10)	20.3(4)
C18	1461.3(10)	2595.5(17)	8565.5(10)	20.4(4)
C19	967.5(10)	2072.7(17)	8236.5(11)	21.6(4)
C20	475.9(9)	2581.5(17)	8019.3(12)	23.0(5)
C21	199.6(9)	2393.4(17)	7345.8(12)	23.2(5)
C22	444.3(9)	1734.4(17)	6920.3(12)	22.0(4)
C23	466.2(9)	1940.6(16)	6213.7(11)	21.5(4)
C24	205.5(9)	2789.9(18)	5945.8(12)	23.9(5)
C25	460.8(10)	3318.4(17)	5439.8(11)	23.6(5)
C26	396.5(10)	4356.9(18)	5561.4(12)	24.6(5)
C27	833.2(10)	4995.9(17)	5455.1(11)	24.8(5)
C28	993.3(10)	5757.1(17)	5945.6(12)	24.8(5)
C29	1610.8(10)	5834.2(16)	6005.4(12)	23.3(5)
C30	1905.7(10)	5999.5(15)	6631.7(12)	21.5(4)
C31	2426.7(9)	5465.1(15)	6816.7(11)	19.2(4)
C32	2445.4(9)	5214.3(15)	7522.5(11)	18.2(4)
C33	2687.1(9)	4331.8(15)	7756.4(10)	17.2(4)
C34	2405.2(9)	3769.8(15)	8251.6(10)	17.1(4)
C35	2449.4(9)	2708.3(15)	8197.2(10)	16.3(4)
C36	1974.3(9)	2167.5(16)	8351.5(10)	17.8(4)
C37	1791.5(9)	1358.8(15)	7929.7(10)	17.8(4)
C38	1182.1(9)	1327.5(16)	7820.3(11)	19.3(4)
C39	950.5(9)	1197.8(15)	7150.7(11)	19.3(4)
C40	1290.2(9)	1003.6(15)	6591.8(11)	18.1(4)
C41	996.4(9)	1545.8(16)	5992.9(11)	18.9(4)
C42	1223.1(9)	2054.3(16)	5469.0(11)	19.2(4)
C43	963.8(10)	2961.1(16)	5231.4(11)	20.9(4)
C44	1411.0(9)	3637.3(17)	5093.7(10)	20.5(4)
C45	1352.6(10)	4626.3(17)	5216.2(11)	22.3(5)
C46	1835.0(10)	5136.0(16)	5557.0(11)	21.6(4)
C47	2353.8(9)	4649.6(16)	5716.3(11)	18.9(4)
C48	2668.0(9)	4843.0(15)	6376.2(11)	18.3(4)
C49	3059.4(9)	4109.6(15)	6667.6(11)	16.7(4)
C50	3064.9(9)	3848.0(15)	7334.0(10)	16.1(4)
C51	3350.9(8)	2908.5(15)	7611.7(10)	15.3(4)
C52	2846.0(9)	2263.8(15)	7761.5(10)	15.8(4)
C53	2680.9(9)	1418.6(15)	7405.0(10)	15.8(4)
C54	2107.7(9)	1091.7(15)	7429.1(10)	16.1(4)

Atom	x	y	z	U_{eq}
C55	1870.7(9)	776.6(14)	6757.6(10)	16.0(4)
C56	1827.2(9)	2087.7(16)	5247.0(10)	18.0(4)
C57	1953.7(10)	3175.6(16)	5172.3(10)	19.1(4)
C58	2434.1(9)	3692.8(16)	5428.5(10)	18.6(4)
C59	3014.9(9)	3247.0(16)	5550.2(10)	17.7(4)
C60	3408.8(9)	3612.3(16)	6172.7(10)	17.6(4)
C61	3734.4(9)	2748.7(15)	6480.0(11)	17.9(4)
C62	3706.0(9)	2443.8(15)	7107.2(10)	16.2(4)
C63	2880.6(9)	805.0(15)	6820.8(10)	16.4(4)
C64	2355.8(9)	497.9(15)	6416.2(10)	16.6(4)
C65	2700.3(9)	-471.8(16)	5132.0(10)	17.4(4)
C66	3144.6(9)	177.8(16)	5073.3(11)	20.3(4)
C67	3448.2(10)	121.7(18)	4512.8(12)	24.7(5)
C68	3309.1(10)	-573.3(19)	4014.8(11)	26.7(5)
C69	2859.2(11)	-1207.0(18)	4067.6(11)	25.1(5)
C70	2554.1(10)	-1161.6(16)	4627.6(11)	21.9(4)
C71	2734.6(9)	-1476.7(16)	6398.6(11)	20.0(4)
C72	3054.5(10)	-2180.2(17)	6096.3(12)	24.6(5)
C73	3369.7(11)	-2861.5(18)	6489.7(14)	30.7(5)
C74	3372.3(11)	-2848.3(18)	7187.6(13)	30.7(5)
C75	3052.4(12)	-2158.9(19)	7487.3(12)	30.9(5)
C76	2733.9(11)	-1474.7(17)	7099.0(11)	24.9(5)
C77	1624.9(9)	-890.9(15)	5681.6(10)	16.7(4)
C78	1357.1(10)	-1491.1(16)	6122.1(11)	21.5(4)
C79	777.9(10)	-1684.2(17)	5997.2(12)	25.2(5)
C80	465.2(10)	-1303.7(17)	5430.4(12)	25.3(5)
C81	730.4(10)	-720.4(18)	4985.0(12)	25.1(5)
C82	1309.1(10)	-508.2(17)	5110.7(11)	21.7(4)
C83	3773.9(9)	3086.3(15)	8246.9(10)	16.8(4)
C84	3772.8(10)	2519.5(16)	8824.3(11)	20.5(4)
C85	4219.6(10)	2629.9(18)	9329.5(12)	24.3(5)
C86	4647.5(10)	3293.8(18)	9240.2(11)	24.5(5)
C87	4616.2(9)	3854.1(17)	8653.6(11)	21.6(4)
C88	5078.1(10)	4584(2)	8503.4(12)	29.2(5)
C89	5513.1(12)	4755(3)	9115.7(14)	46.6(8)
C90	5392.0(10)	4157(2)	7924.8(12)	31.6(6)
C91	4802.9(12)	5561(2)	8292.8(15)	38.3(6)
C92	3834.3(9)	4391.4(16)	5970.7(11)	21.2(4)
C93	4243.8(10)	4727.2(18)	6479.0(13)	28.3(5)
C94	4611.0(11)	5457(2)	6320.4(14)	32.7(6)
C95	4557.7(11)	5825.0(19)	5671.4(14)	32.8(6)
C96	4138.5(11)	5471.9(18)	5194.5(12)	27.0(5)
C97	4016.6(12)	5924.6(18)	4493.8(13)	30.6(5)
C98	4537.9(15)	6467(2)	4271.7(15)	42.7(7)
C99	3542.7(14)	6676(2)	4552.1(14)	40.9(7)
C100	3812.8(13)	5152.6(19)	3965.4(13)	34.8(6)

Table S3.1.2 Anisotropic Displacement Parameters ($\times 10^4$) open fullerene **4**. The anisotropic displacement factor exponent takes the form: $-2\square^2[h^2a^{*2} \times U_{11} + ... +2hka^* \times b^* \times U_{12}]$

Atom	U_{11}	U_{22}	U_{33}	U_{23}	U_{13}	U_{12}
P1	16.7(2)	14.3(2)	15.6(2)	-1.38(19)	2.10(19)	-0.3(2)
O1	24.4(8)	21.8(8)	23.9(8)	-1.3(6)	2.7(6)	3.7(7)
O2	17.1(7)	19.5(7)	24.4(8)	-3.2(6)	2.3(6)	1.4(6)
O3	25.2(8)	28.1(9)	20.1(8)	-1.8(7)	5.6(6)	3.0(7)
N1	15.0(8)	22.8(9)	19.9(9)	-2.9(7)	0.5(7)	-1.8(7)
N2	27.2(10)	20.6(9)	25.3(10)	2.9(8)	8.7(8)	3.0(8)
C1	15.7(10)	27.6(12)	34.9(12)	-12.1(10)	3.9(9)	6.5(9)
C2	19.4(10)	20.9(11)	32.7(12)	-11.2(9)	4.5(9)	4.6(9)
C3	20.6(11)	24.4(11)	24.9(11)	-12.1(9)	6.0(9)	1.6(9)
C4	21.7(11)	27.0(12)	21.5(11)	-8.9(9)	9.1(9)	0.1(9)
C5	17.0(10)	30.4(12)	28.0(11)	-9.0(10)	10.1(9)	0.1(9)
C6	13.4(10)	30.4(12)	33.8(12)	-11.9(10)	6.3(9)	2.6(9)

Atom	U_{11}	U_{22}	U_{33}	U_{23}	U_{13}	U_{12}
C7	10.0(9)	28.8(12)	36.2(13)	-10.1(10)	3.4(9)	0.2(8)
C8	9.3(9)	28.9(12)	36.1(13)	-10.4(10)	-5.1(9)	3.2(8)
C9	14.2(10)	28.9(12)	34.0(12)	-5.1(10)	-5.3(9)	9.2(9)
C10	17.3(10)	24.5(12)	36.4(13)	-7.9(10)	-3.1(9)	10.3(9)
C11	20.0(11)	20.1(11)	34.6(13)	-4.8(9)	-4.7(9)	9.0(9)
C12	22.4(11)	15.6(10)	32.2(12)	-6.4(9)	-0.6(9)	6.1(8)
C13	24.4(11)	13.6(10)	29.1(11)	-5.8(9)	-0.1(9)	2.0(8)
C14	20.8(10)	15.0(10)	26.7(11)	-7.7(8)	-1.8(8)	-1.3(8)
C15	20.1(10)	20.7(11)	20.9(10)	-10.0(8)	2.2(8)	0.1(8)
C16	21.1(10)	21.6(11)	15.1(9)	-7.3(8)	1.5(8)	-2.2(8)
C17	20.0(10)	26.6(11)	15.1(9)	-4.7(8)	5.7(8)	-2.5(9)
C18	22.0(11)	24.9(11)	15.1(9)	-1.2(8)	6.2(8)	-3.6(9)
C19	20.0(10)	25.7(11)	20.5(10)	-2.5(9)	8.7(8)	-7.2(9)
C20	16.9(10)	27.3(12)	26.5(11)	-7.5(9)	10.8(9)	-7.0(9)
C21	13.5(10)	26.0(11)	31.0(12)	-5.6(9)	7.0(9)	-6.8(8)
C22	14.6(10)	22.2(11)	29.4(11)	-6.4(9)	2.8(8)	-7.6(8)
C23	14.5(10)	22.3(11)	27.1(11)	-8.4(9)	-1.7(8)	-3.5(8)
C24	12.9(10)	27.2(12)	30.2(12)	-9.3(10)	-4.3(8)	-0.4(9)
C25	18.7(10)	26.4(12)	23.7(11)	-5.1(9)	-8.6(8)	3.3(9)
C26	17.7(10)	26.6(12)	27.4(11)	-4.5(9)	-8.3(9)	8.8(9)
C27	25.3(11)	24.2(11)	22.9(11)	0.6(9)	-6.5(9)	8.9(9)
C28	25.2(11)	18.2(11)	29.4(12)	1.3(9)	-4.7(9)	7.1(9)
C29	25.8(11)	15.1(10)	27.8(11)	3.2(9)	-2.8(9)	4.5(9)
C30	24.3(11)	11.0(9)	28.6(11)	0.3(8)	0.2(9)	1.0(8)
C31	18.9(10)	14.0(10)	24.3(10)	0.0(8)	-0.1(8)	-3.9(8)
C32	16.8(10)	14.8(10)	22.4(10)	-5.2(8)	-0.7(8)	-3.9(8)
C33	14.9(9)	17.8(10)	18.4(10)	-4.4(8)	-1.5(8)	-3.0(8)
C34	17.9(10)	18.8(10)	14.2(9)	-3.2(8)	-0.5(8)	-2.7(8)
C35	17.7(10)	19.0(10)	11.8(9)	-0.2(8)	-0.3(7)	-1.3(8)
C36	20.6(10)	19.1(10)	13.8(9)	-0.6(8)	2.4(8)	-1.3(8)
C37	20.0(10)	16.5(10)	17.5(10)	1.8(8)	4.2(8)	-3.3(8)
C38	19.6(10)	18.3(10)	21.0(10)	-1.1(8)	7.5(8)	-4.4(8)
C39	15.3(10)	17.1(10)	26.0(11)	-3.2(8)	4.3(8)	-6.1(8)
C40	17.2(10)	15.2(10)	21.9(10)	-4.3(8)	1.6(8)	-3.2(8)
C41	14.9(9)	17.9(10)	22.9(10)	-7.8(8)	-2.5(8)	-2.8(8)
C42	17.5(10)	20.2(10)	19.0(10)	-6.6(8)	-3.0(8)	0.6(8)
C43	21.7(11)	22.4(11)	17.2(10)	-4.8(8)	-5.4(8)	1.8(9)
C44	20.9(10)	24.1(11)	15.5(10)	0.2(8)	-3.4(8)	3.3(9)
C45	25.8(11)	22.9(11)	16.9(10)	3.5(8)	-4.8(8)	4.7(9)
C46	25.7(11)	17.9(10)	20.4(10)	2.4(8)	-0.9(9)	2.7(9)
C47	21.4(10)	17.3(10)	18.0(10)	4.3(8)	2.7(8)	0.4(8)
C48	18.2(10)	15.9(10)	20.5(10)	1.5(8)	1.5(8)	-2.4(8)
C49	14.0(9)	13.9(9)	22.3(10)	-1.5(8)	2.4(8)	-3.6(7)
C50	13.4(9)	15.1(9)	19.3(10)	-2.1(8)	-1.0(8)	-2.8(7)
C51	13.1(9)	16.3(10)	16.0(9)	-0.4(8)	-0.6(7)	-1.3(7)
C52	15.0(9)	19.0(10)	13.2(9)	1.5(8)	0.4(7)	0.5(8)
C53	16.0(9)	17.2(10)	13.9(9)	1.6(8)	0.2(7)	0.0(8)
C54	16.8(9)	13.4(9)	17.8(9)	-0.3(8)	0.3(8)	-0.2(8)
C55	18.5(10)	12.2(9)	17.2(9)	-1.2(7)	1.4(8)	-3.9(7)
C56	19.4(10)	20.1(10)	13.8(9)	-2.2(8)	-2.2(8)	1.1(8)
C57	23.5(11)	20.4(10)	13.1(9)	-0.9(8)	0.0(8)	2.6(8)
C58	23.3(11)	19.9(10)	12.7(9)	3.0(8)	2.0(8)	1.9(8)
C59	18.8(10)	18.5(10)	16.5(9)	3.1(8)	4.2(8)	-0.7(8)
C60	15.9(9)	18.5(10)	18.7(10)	2.3(8)	3.7(8)	-0.8(8)
C61	14.5(9)	17.2(10)	22.3(10)	-0.4(8)	3.7(8)	-0.2(8)
C62	13.0(9)	15.3(10)	20.3(10)	-2.0(8)	1.2(8)	-1.0(7)
C63	19.1(10)	12.8(9)	17.4(9)	1.0(7)	1.6(8)	0.2(8)
C64	18.0(10)	14.8(9)	16.8(9)	-3.7(8)	0.5(8)	-0.6(8)
C65	19.2(10)	18.8(10)	14.7(9)	0.7(8)	4.1(8)	3.9(8)
C66	18.9(10)	20.4(10)	21.1(10)	2.2(8)	0.4(8)	2.5(8)
C67	18.9(11)	29.1(12)	26.7(11)	8.7(10)	5.0(9)	1.6(9)
C68	27.0(12)	36.2(13)	17.6(10)	6.2(9)	5.2(9)	12.9(10)
C69	31.4(12)	25.0(12)	18.4(10)	-0.4(9)	1.0(9)	10.1(10)

Atom	U_{11}	U_{22}	U_{33}	U_{23}	U_{13}	U_{12}
C70	24.6(11)	19.7(11)	21.3(10)	-0.1(8)	2.3(9)	2.3(9)
C71	20.8(10)	16.4(10)	22.6(10)	1.6(8)	1.0(8)	-0.9(8)
C72	28.4(12)	22.0(11)	24.3(11)	1.8(9)	6.7(9)	2.8(9)
C73	29.5(13)	21.9(12)	41.7(14)	5.1(10)	9.3(11)	5.3(10)
C74	29.6(13)	22.4(12)	38.4(14)	10.8(10)	-5.3(10)	-3.5(10)
C75	42.8(15)	26.7(12)	21.9(11)	4.1(10)	-2.9(10)	-6.0(11)
C76	32.5(13)	21.2(11)	21.2(11)	-0.2(9)	2.8(9)	-1.9(10)
C77	16.8(10)	14.4(9)	19.3(10)	-4.1(8)	3.9(8)	-1.9(8)
C78	24.3(11)	16.7(10)	23.8(11)	0.7(8)	3.6(9)	0.2(9)
C79	25.6(12)	18.6(11)	32.6(12)	-1.0(9)	9.8(10)	-2.5(9)
C80	17.4(10)	25.4(12)	33.1(12)	-7.6(10)	2.6(9)	-2.1(9)
C81	21.7(11)	29.2(12)	23.2(11)	-3.0(9)	-3.1(9)	-1.7(9)
C82	23.7(11)	21.8(11)	19.4(10)	-0.6(9)	1.2(8)	-3.2(9)
C83	12.9(9)	18.6(10)	18.4(10)	-4.1(8)	-0.3(7)	1.6(8)
C84	20.6(10)	18.7(10)	21.8(10)	0.4(8)	0.6(8)	-0.1(8)
C85	22.4(11)	27.6(12)	22.2(11)	1.7(9)	-0.8(9)	4.3(9)
C86	18.3(10)	31.8(12)	22.2(11)	-3.7(9)	-4.2(8)	3.4(9)
C87	14.6(10)	27.1(11)	23.2(11)	-7.4(9)	1.8(8)	-1.1(8)
C88	18.9(11)	41.5(14)	26.9(12)	-6.1(11)	0.2(9)	-10.2(10)
C89	30.7(14)	80(2)	29.1(13)	-10.2(14)	2.1(11)	-30.5(15)
C90	17.6(11)	50.7(16)	26.7(12)	-4.0(11)	3.8(9)	-3.3(11)
C91	34.1(14)	33.1(14)	49.5(16)	-6.9(12)	13.0(12)	-16.6(12)
C92	18.6(10)	19.0(10)	26.7(11)	4.4(9)	6.2(8)	2.5(8)
C93	21.8(11)	28.2(12)	34.7(13)	5.6(10)	1.6(10)	-2.8(9)
C94	24.7(12)	31.9(13)	40.9(14)	3.1(11)	0.3(10)	-6.7(10)
C95	25.4(12)	28.9(13)	45.0(15)	7.1(11)	8.2(11)	-4.0(10)
C96	32.2(13)	22.8(11)	27.9(12)	5.9(9)	13.2(10)	6.2(10)
C97	44.1(15)	22.0(12)	27.6(12)	5.3(10)	12.3(11)	4.4(11)
C98	61(2)	31.9(15)	38.1(15)	2.1(12)	21.4(14)	-7.5(14)
C99	56.1(19)	35.6(15)	30.0(13)	1.7(11)	-0.5(12)	16.6(13)
C100	49.1(17)	28.1(13)	29.1(13)	5.3(10)	12.7(12)	4.8(12)

Table S3.1.3 Bond Lengths in Å for open fullerene **4**.

Atom	Atom	Length/Å
P1	C64	1.742(2)
P1	C65	1.793(2)
P1	C71	1.814(2)
P1	C77	1.797(2)
O1	C56	1.202(3)
O2	C63	1.223(3)
O3	C59	1.208(3)
N1	C83	1.338(3)
N1	C87	1.350(3)
N2	C92	1.328(3)
N2	C96	1.365(3)
C1	C2	1.451(3)
C1	C6	1.385(4)
C1	C10	1.455(4)
C2	C3	1.391(3)
C2	C12	1.447(3)
C3	C4	1.441(3)
C3	C15	1.449(3)
C4	C5	1.394(3)
C4	C17	1.452(3)
C5	C6	1.445(4)
C5	C20	1.449(3)
C6	C7	1.450(3)
C7	C8	1.387(4)
C7	C21	1.438(3)
C8	C9	1.447(4)
C8	C24	1.453(3)
C9	C10	1.393(3)

Atom	Atom	Length/Å
C9	C26	1.444(3)
C10	C11	1.446(4)
C11	C12	1.451(3)
C11	C28	1.391(4)
C12	C13	1.385(3)
C13	C14	1.447(3)
C13	C30	1.442(3)
C14	C15	1.375(3)
C14	C32	1.434(3)
C15	C16	1.448(3)
C16	C17	1.442(3)
C16	C34	1.403(3)
C17	C18	1.371(3)
C18	C19	1.460(3)
C18	C36	1.443(3)
C19	C20	1.380(3)
C19	C38	1.435(3)
C20	C21	1.449(3)
C21	C22	1.398(3)
C22	C23	1.436(3)
C22	C39	1.433(3)
C23	C24	1.393(3)
C23	C41	1.466(3)
C24	C25	1.419(3)
C25	C26	1.451(3)
C25	C43	1.381(3)
C26	C27	1.381(4)
C27	C28	1.449(3)

Atom	Atom	Length/Å
C27	C45	1.445(3)
C28	C29	1.448(3)
C29	C30	1.379(3)
C29	C46	1.441(3)
C30	C31	1.441(3)
C31	C32	1.438(3)
C31	C48	1.382(3)
C32	C33	1.393(3)
C33	C34	1.459(3)
C33	C50	1.443(3)
C34	C35	1.460(3)
C35	C36	1.399(3)
C35	C52	1.466(3)
C36	C37	1.427(3)
C37	C38	1.428(3)
C37	C54	1.350(3)
C38	C39	1.395(3)
C39	C40	1.455(3)
C40	C41	1.508(3)
C40	C55	1.406(3)
C41	C42	1.400(3)
C42	C43	1.440(3)
C42	C56	1.530(3)
C43	C44	1.447(3)
C44	C45	1.384(3)
C44	C57	1.418(3)
C45	C46	1.440(3)
C46	C47	1.397(3)
C47	C48	1.461(3)
C47	C58	1.448(3)
C48	C49	1.442(3)
C49	C50	1.369(3)
C49	C60	1.505(3)
C50	C51	1.527(3)
C51	C52	1.532(3)
C51	C62	1.508(3)
C51	C83	1.544(3)
C52	C53	1.390(3)
C53	C54	1.425(3)
C53	C63	1.542(3)
C54	C55	1.457(3)
C55	C64	1.436(3)

Atom	Atom	Length/Å
C56	C57	1.527(3)
C57	C58	1.385(3)
C58	C59	1.493(3)
C59	C60	1.550(3)
C60	C61	1.503(3)
C60	C92	1.542(3)
C61	C62	1.321(3)
C63	C64	1.464(3)
C65	C66	1.385(3)
C65	C70	1.393(3)
C66	C67	1.383(3)
C67	C68	1.386(4)
C68	C69	1.380(4)
C69	C70	1.384(3)
C71	C72	1.394(3)
C71	C76	1.390(3)
C72	C73	1.382(3)
C73	C74	1.385(4)
C74	C75	1.378(4)
C75	C76	1.383(3)
C77	C78	1.395(3)
C77	C82	1.392(3)
C78	C79	1.384(3)
C79	C80	1.382(3)
C80	C81	1.385(3)
C81	C82	1.389(3)
C83	C84	1.384(3)
C84	C85	1.385(3)
C85	C86	1.380(3)
C86	C87	1.390(3)
C87	C88	1.526(3)
C88	C89	1.525(3)
C88	C90	1.541(3)
C88	C91	1.525(4)
C92	C93	1.400(3)
C93	C94	1.377(3)
C94	C95	1.377(4)
C95	C96	1.382(4)
C96	C97	1.523(3)
C97	C98	1.535(4)
C97	C99	1.528(4)
C97	C100	1.530(4)

Table S3.1.4 Bond Angles (°) for open fullerene **4**..

Atom	Atom	Atom	Angle/°
C64	P1	C65	119.58(10)
C64	P1	C71	106.28(10)
C64	P1	C77	107.81(10)
C65	P1	C71	104.87(10)
C65	P1	C77	108.31(10)
C77	P1	C71	109.72(10)
C83	N1	C87	118.72(19)
C92	N2	C96	117.3(2)
C2	C1	C10	107.9(2)
C6	C1	C2	119.8(2)
C6	C1	C10	120.3(2)
C3	C2	C1	119.4(2)
C3	C2	C12	120.3(2)
C12	C2	C1	107.8(2)
C2	C3	C4	120.6(2)
C2	C3	C15	119.9(2)

Atom	Atom	Atom	Angle/°
C4	C3	C15	107.4(2)
C3	C4	C17	107.53(19)
C5	C4	C3	119.8(2)
C5	C4	C17	119.8(2)
C4	C5	C6	119.6(2)
C4	C5	C20	119.9(2)
C6	C5	C20	108.4(2)
C1	C6	C5	120.7(2)
C1	C6	C7	119.8(2)
C5	C6	C7	107.5(2)
C8	C7	C6	119.9(2)
C8	C7	C21	120.1(2)
C21	C7	C6	108.3(2)
C7	C8	C9	120.5(2)
C7	C8	C24	119.9(2)
C9	C8	C24	107.7(2)

Atom	Atom	Atom	Angle/°
C10	C9	C8	119.6(2)
C10	C9	C26	120.0(2)
C26	C9	C8	108.0(2)
C9	C10	C1	119.8(2)
C9	C10	C11	119.7(2)
C11	C10	C1	108.1(2)
C10	C11	C12	107.8(2)
C28	C11	C10	120.1(2)
C28	C11	C12	119.8(2)
C2	C12	C11	108.4(2)
C13	C12	C2	119.4(2)
C13	C12	C11	119.7(2)
C12	C13	C14	120.2(2)
C12	C13	C30	120.1(2)
C30	C13	C14	106.89(19)
C15	C14	C13	120.6(2)
C15	C14	C32	119.1(2)
C32	C14	C13	108.9(2)
C14	C15	C3	119.6(2)
C14	C15	C16	119.7(2)
C16	C15	C3	109.3(2)
C17	C16	C15	106.31(19)
C34	C16	C15	121.9(2)
C34	C16	C17	122.0(2)
C16	C17	C4	109.3(2)
C18	C17	C4	120.0(2)
C18	C17	C16	119.4(2)
C17	C18	C19	120.4(2)
C17	C18	C36	118.7(2)
C36	C18	C19	108.67(19)
C20	C19	C18	119.7(2)
C20	C19	C38	120.7(2)
C38	C19	C18	107.17(19)
C19	C20	C5	120.1(2)
C19	C20	C21	118.8(2)
C21	C20	C5	107.4(2)
C7	C21	C20	108.3(2)
C22	C21	C7	119.4(2)
C22	C21	C20	119.9(2)
C21	C22	C23	121.4(2)
C21	C22	C39	121.1(2)
C39	C22	C23	107.54(19)
C22	C23	C41	109.59(19)
C24	C23	C22	118.6(2)
C24	C23	C41	123.2(2)
C23	C24	C8	120.5(2)
C23	C24	C25	119.6(2)
C25	C24	C8	108.1(2)
C24	C25	C26	108.8(2)
C43	C25	C24	117.9(2)
C43	C25	C26	120.2(2)
C9	C26	C25	107.5(2)
C27	C26	C9	120.3(2)
C27	C26	C25	120.2(2)
C26	C27	C28	119.9(2)
C26	C27	C45	119.7(2)
C45	C27	C28	107.5(2)
C11	C28	C27	119.9(2)
C11	C28	C29	120.2(2)
C29	C28	C27	107.3(2)
C30	C29	C28	119.6(2)
C30	C29	C46	118.9(2)
C46	C29	C28	109.1(2)
C29	C30	C13	120.6(2)
C29	C30	C31	118.9(2)
C31	C30	C13	108.45(19)
C32	C31	C30	108.08(19)
C48	C31	C30	122.9(2)
C48	C31	C32	119.67(19)
C14	C32	C31	107.63(19)
C33	C32	C14	122.5(2)
C33	C32	C31	120.15(19)
C32	C33	C34	118.90(19)
C32	C33	C50	117.26(19)
C50	C33	C34	119.89(19)
C16	C34	C33	117.88(19)
C16	C34	C35	119.25(19)
C33	C34	C35	115.70(18)
C34	C35	C52	120.66(18)
C36	C35	C34	116.43(19)
C36	C35	C52	118.63(19)
C35	C36	C18	124.0(2)
C35	C36	C37	118.76(18)
C37	C36	C18	106.03(18)
C36	C37	C38	110.51(19)
C54	C37	C36	119.07(19)
C54	C37	C38	119.93(19)
C37	C38	C19	107.28(19)
C39	C38	C19	120.8(2)
C39	C38	C37	115.93(19)
C22	C39	C40	110.63(19)
C38	C39	C22	118.0(2)
C38	C39	C40	123.91(19)
C39	C40	C41	105.56(18)
C55	C40	C39	117.16(19)
C55	C40	C41	130.50(19)
C23	C41	C40	106.32(18)
C42	C41	C23	116.0(2)
C42	C41	C40	130.63(19)
C41	C42	C43	119.4(2)
C41	C42	C56	131.53(19)
C43	C42	C56	104.73(19)
C25	C43	C42	123.6(2)
C25	C43	C44	119.4(2)
C42	C43	C44	108.71(19)
C45	C44	C43	120.3(2)
C45	C44	C57	121.3(2)
C57	C44	C43	110.9(2)
C44	C45	C27	120.1(2)
C44	C45	C46	117.9(2)
C46	C45	C27	109.1(2)
C45	C46	C29	106.9(2)
C47	C46	C29	122.8(2)
C47	C46	C45	120.2(2)
C46	C47	C48	117.85(19)
C46	C47	C58	119.1(2)
C58	C47	C48	116.30(18)
C31	C48	C47	118.40(19)
C31	C48	C49	117.43(19)
C49	C48	C47	118.53(19)
C48	C49	C60	114.89(18)
C50	C49	C48	120.83(19)
C50	C49	C60	124.06(19)
C33	C50	C51	116.80(18)
C49	C50	C33	119.70(19)
C49	C50	C51	121.97(18)

Atom	Atom	Atom	Angle/°
C50	C51	C52	103.47(16)
C50	C51	C83	112.70(17)
C52	C51	C83	112.34(16)
C62	C51	C50	111.61(17)
C62	C51	C52	112.23(17)
C62	C51	C83	104.74(16)
C35	C52	C51	115.30(18)
C53	C52	C35	118.92(18)
C53	C52	C51	124.19(18)
C52	C53	C54	117.39(18)
C52	C53	C63	138.12(19)
C54	C53	C63	102.44(17)
C37	C54	C53	122.20(19)
C37	C54	C55	124.4(2)
C53	C54	C55	109.73(18)
C40	C55	C54	114.91(18)
C40	C55	C64	138.32(19)
C64	C55	C54	105.16(17)
O1	C56	C42	127.3(2)
O1	C56	C57	128.1(2)
C57	C56	C42	104.64(17)
C44	C57	C56	105.23(19)
C58	C57	C44	120.3(2)
C58	C57	C56	128.42(19)
C47	C58	C59	117.32(19)
C57	C58	C47	118.2(2)
C57	C58	C59	123.3(2)
O3	C59	C58	121.25(19)
O3	C59	C60	121.34(19)
C58	C59	C60	117.40(18)
C49	C60	C59	110.14(17)
C49	C60	C92	105.59(17)
C61	C60	C49	112.30(17)
C61	C60	C59	107.99(17)
C61	C60	C92	109.35(17)
C92	C60	C59	111.50(17)
C62	C61	C60	123.94(19)
C61	C62	C51	125.24(19)
O2	C63	C53	128.13(19)
O2	C63	C64	126.26(19)
C64	C63	C53	105.34(17)
C55	C64	P1	122.73(15)
C55	C64	C63	109.31(17)
C63	C64	P1	120.38(15)
C66	C65	P1	120.53(16)
C66	C65	C70	120.49(19)
C70	C65	P1	118.64(17)
C67	C66	C65	119.1(2)
C66	C67	C68	120.5(2)
C69	C68	C67	120.3(2)

Atom	Atom	Atom	Angle/°
C68	C69	C70	119.8(2)
C69	C70	C65	119.8(2)
C72	C71	P1	120.91(17)
C76	C71	P1	119.84(17)
C76	C71	C72	119.2(2)
C73	C72	C71	120.3(2)
C72	C73	C74	120.3(2)
C75	C74	C73	119.6(2)
C74	C75	C76	120.7(2)
C75	C76	C71	120.0(2)
C78	C77	P1	119.67(17)
C82	C77	P1	120.49(16)
C82	C77	C78	119.6(2)
C79	C78	C77	119.9(2)
C80	C79	C78	120.4(2)
C79	C80	C81	120.0(2)
C80	C81	C82	120.1(2)
C81	C82	C77	120.0(2)
N1	C83	C51	114.30(18)
N1	C83	C84	123.00(19)
C84	C83	C51	122.20(19)
C83	C84	C85	118.3(2)
C86	C85	C84	119.2(2)
C85	C86	C87	119.6(2)
N1	C87	C86	121.2(2)
N1	C87	C88	115.6(2)
C86	C87	C88	123.1(2)
C87	C88	C90	107.6(2)
C89	C88	C87	112.2(2)
C89	C88	C90	108.6(2)
C91	C88	C87	109.6(2)
C91	C88	C89	108.4(2)
C91	C88	C90	110.5(2)
N2	C92	C60	118.9(2)
N2	C92	C93	123.7(2)
C93	C92	C60	117.15(19)
C94	C93	C92	118.3(2)
C95	C94	C93	118.6(2)
C94	C95	C96	120.2(2)
N2	C96	C95	121.7(2)
N2	C96	C97	115.5(2)
C95	C96	C97	122.6(2)
C96	C97	C98	112.1(2)
C96	C97	C99	106.0(2)
C96	C97	C100	111.2(2)
C99	C97	C98	107.9(2)
C99	C97	C100	109.6(2)
C100	C97	C98	109.9(2)

Table S3.1.5 Torsion Angles (°) for open fullerene **4**.

Atom	Atom	Atom	Atom	Angle/°
P1	C65	C66	C67	172.06(17)
P1	C65	C70	C69	-172.52(17)
P1	C71	C72	C73	175.95(19)
P1	C71	C76	C75	-175.82(19)
P1	C77	C78	C79	172.84(17)
P1	C77	C82	C81	-173.85(17)
O1	C56	C57	C44	157.9(2)
O1	C56	C57	C58	-50.1(3)

Atom	Atom	Atom	Atom	Angle/°
O2	C63	C64	P1	17.1(3)
O2	C63	C64	C55	167.5(2)
O3	C59	C60	C49	-160.50(19)
O3	C59	C60	C61	-37.5(3)
O3	C59	C60	C92	82.6(2)
N1	C83	C84	C85	-1.2(3)
N1	C87	C88	C89	173.0(2)
N1	C87	C88	C90	-67.6(3)
N1	C87	C88	C91	52.5(3)
N2	C92	C93	C94	-1.4(4)
N2	C96	C97	C98	-161.3(2)
N2	C96	C97	C99	81.3(3)
N2	C96	C97	C100	-37.8(3)
C1	C2	C3	C4	-2.7(3)
C1	C2	C3	C15	135.2(2)
C1	C2	C12	C11	0.2(2)
C1	C2	C12	C13	-141.8(2)
C1	C6	C7	C8	-0.2(3)
C1	C6	C7	C21	142.8(2)
C1	C10	C11	C12	0.9(2)
C1	C10	C11	C28	143.0(2)
C2	C1	C6	C5	1.5(3)
C2	C1	C6	C7	-136.5(2)
C2	C1	C10	C9	141.2(2)
C2	C1	C10	C11	-0.8(2)
C2	C3	C4	C5	1.9(3)
C2	C3	C4	C17	143.3(2)
C2	C3	C15	C14	2.3(3)
C2	C3	C15	C16	-141.0(2)
C2	C12	C13	C14	2.1(3)
C2	C12	C13	C30	138.6(2)
C3	C2	C12	C11	141.8(2)
C3	C2	C12	C13	-0.1(3)
C3	C4	C5	C6	0.7(3)
C3	C4	C5	C20	138.8(2)
C3	C4	C17	C16	-3.1(2)
C3	C4	C17	C18	-146.3(2)
C3	C15	C16	C17	-3.7(2)
C3	C15	C16	C34	142.7(2)
C4	C3	C15	C14	145.2(2)
C4	C3	C15	C16	1.9(2)
C4	C5	C6	C1	-2.4(3)
C4	C5	C6	C7	140.1(2)
C4	C5	C20	C19	1.4(3)
C4	C5	C20	C21	-138.5(2)
C4	C17	C18	C19	4.2(3)
C4	C17	C18	C36	142.2(2)
C5	C4	C17	C16	138.3(2)
C5	C4	C17	C18	-4.9(3)
C5	C6	C7	C8	-143.1(2)
C5	C6	C7	C21	0.0(2)
C5	C20	C21	C7	-3.8(2)
C5	C20	C21	C22	138.1(2)
C6	C1	C2	C3	1.1(3)
C6	C1	C2	C12	143.1(2)
C6	C1	C10	C9	-1.3(3)
C6	C1	C10	C11	-143.3(2)
C6	C5	C20	C19	143.6(2)
C6	C5	C20	C21	3.7(2)
C6	C7	C8	C9	-1.6(3)
C6	C7	C8	C24	136.6(2)
C6	C7	C21	C20	2.4(2)
C6	C7	C21	C22	-139.7(2)

Atom	Atom	Atom	Atom	Angle/°
C7	C8	C9	C10	1.9(3)
C7	C8	C9	C26	144.0(2)
C7	C8	C24	C23	-2.2(3)
C7	C8	C24	C25	-144.8(2)
C7	C21	C22	C23	-0.2(3)
C7	C21	C22	C39	141.0(2)
C8	C7	C21	C20	145.3(2)
C8	C7	C21	C22	3.3(3)
C8	C9	C10	C1	-0.5(3)
C8	C9	C10	C11	137.2(2)
C8	C9	C26	C25	-0.3(2)
C8	C9	C26	C27	-142.9(2)
C8	C24	C25	C26	1.7(2)
C8	C24	C25	C43	143.1(2)
C9	C8	C24	C23	140.7(2)
C9	C8	C24	C25	-1.9(2)
C9	C10	C11	C12	-141.2(2)
C9	C10	C11	C28	0.9(3)
C9	C26	C27	C28	1.8(3)
C9	C26	C27	C45	138.5(2)
C10	C1	C2	C3	-141.6(2)
C10	C1	C2	C12	0.4(2)
C10	C1	C6	C5	139.6(2)
C10	C1	C6	C7	1.7(3)
C10	C9	C26	C25	141.6(2)
C10	C9	C26	C27	-1.0(3)
C10	C11	C12	C2	-0.7(2)
C10	C11	C12	C13	141.1(2)
C10	C11	C28	C27	-0.1(3)
C10	C11	C28	C29	-137.1(2)
C11	C12	C13	C14	-135.6(2)
C11	C12	C13	C30	0.9(3)
C11	C28	C29	C30	0.3(3)
C11	C28	C29	C46	142.0(2)
C12	C2	C3	C4	-140.0(2)
C12	C2	C3	C15	-2.1(3)
C12	C11	C28	C27	137.4(2)
C12	C11	C28	C29	0.4(3)
C12	C13	C14	C15	-1.9(3)
C12	C13	C14	C32	141.3(2)
C12	C13	C30	C29	-0.1(3)
C12	C13	C30	C31	-142.2(2)
C13	C14	C15	C3	-0.4(3)
C13	C14	C15	C16	139.2(2)
C13	C14	C32	C31	1.0(2)
C13	C14	C32	C33	-144.7(2)
C13	C30	C31	C32	1.3(2)
C13	C30	C31	C48	147.6(2)
C14	C13	C30	C29	141.4(2)
C14	C13	C30	C31	-0.7(2)
C14	C15	C16	C17	-147.0(2)
C14	C15	C16	C34	-0.6(3)
C14	C32	C33	C34	3.1(3)
C14	C32	C33	C50	160.72(19)
C15	C3	C4	C5	-140.7(2)
C15	C3	C4	C17	0.8(2)
C15	C14	C32	C31	144.8(2)
C15	C14	C32	C33	-0.9(3)
C15	C16	C17	C4	4.2(2)
C15	C16	C17	C18	147.7(2)
C15	C16	C34	C33	2.7(3)
C15	C16	C34	C35	-146.4(2)
C16	C17	C18	C19	-135.3(2)

Atom	Atom	Atom	Atom	Angle/°
C16	C17	C18	C36	2.7(3)
C16	C34	C35	C36	4.9(3)
C16	C34	C35	C52	161.35(19)
C17	C4	C5	C6	-136.1(2)
C17	C4	C5	C20	2.1(3)
C17	C16	C34	C33	143.9(2)
C17	C16	C34	C35	-5.2(3)
C17	C18	C19	C20	-0.8(3)
C17	C18	C19	C38	141.5(2)
C17	C18	C36	C35	-2.9(3)
C17	C18	C36	C37	-145.8(2)
C18	C19	C20	C5	-2.1(3)
C18	C19	C20	C21	133.4(2)
C18	C19	C38	C37	3.6(2)
C18	C19	C38	C39	-132.3(2)
C18	C36	C37	C38	5.7(2)
C18	C36	C37	C54	150.5(2)
C19	C18	C36	C35	139.6(2)
C19	C18	C36	C37	-3.3(2)
C19	C20	C21	C7	-144.3(2)
C19	C20	C21	C22	-2.5(3)
C19	C38	C39	C22	-8.4(3)
C19	C38	C39	C40	138.6(2)
C20	C5	C6	C1	-144.8(2)
C20	C5	C6	C7	-2.3(2)
C20	C19	C38	C37	145.5(2)
C20	C19	C38	C39	9.5(3)
C20	C21	C22	C23	-137.9(2)
C20	C21	C22	C39	3.4(3)
C21	C7	C8	C9	-140.3(2)
C21	C7	C8	C24	-2.1(3)
C21	C22	C23	C24	-3.9(3)
C21	C22	C23	C41	144.9(2)
C21	C22	C39	C38	2.1(3)
C21	C22	C39	C40	-149.0(2)
C22	C23	C24	C8	5.1(3)
C22	C23	C24	C25	143.6(2)
C22	C23	C41	C40	4.4(2)
C22	C23	C41	C42	-149.46(19)
C22	C39	C40	C41	5.7(2)
C22	C39	C40	C55	159.93(19)
C23	C22	C39	C38	148.0(2)
C23	C22	C39	C40	-3.1(2)
C23	C24	C25	C26	-141.3(2)
C23	C24	C25	C43	0.1(3)
C23	C41	C42	C43	6.0(3)
C23	C41	C42	C56	158.7(2)
C24	C8	C9	C10	-140.7(2)
C24	C8	C9	C26	1.4(2)
C24	C23	C41	C40	151.5(2)
C24	C23	C41	C42	-2.4(3)
C24	C25	C26	C9	-0.9(2)
C24	C25	C26	C27	141.8(2)
C24	C25	C43	C42	3.9(3)
C24	C25	C43	C44	-140.9(2)
C25	C26	C27	C28	-136.1(2)
C25	C26	C27	C45	0.6(3)
C25	C43	C44	C45	4.6(3)
C25	C43	C44	C57	155.0(2)
C26	C9	C10	C1	-138.0(2)
C26	C9	C10	C11	-0.3(3)
C26	C25	C43	C42	140.8(2)
C26	C25	C43	C44	-4.0(3)

Atom	Atom	Atom	Atom	Angle/°
C26	C27	C28	C11	-1.2(3)
C26	C27	C28	C29	140.6(2)
C26	C27	C45	C44	0.1(3)
C26	C27	C45	C46	-140.6(2)
C27	C28	C29	C30	-141.4(2)
C27	C28	C29	C46	0.3(3)
C27	C45	C46	C29	-0.6(2)
C27	C45	C46	C47	145.7(2)
C28	C11	C12	C2	-142.8(2)
C28	C11	C12	C13	-1.0(3)
C28	C27	C45	C44	141.5(2)
C28	C27	C45	C46	0.8(3)
C28	C29	C30	C13	-0.5(3)
C28	C29	C30	C31	137.8(2)
C28	C29	C46	C45	0.2(2)
C28	C29	C46	C47	-145.1(2)
C29	C30	C31	C32	-141.5(2)
C29	C30	C31	C48	4.8(3)
C29	C46	C47	C48	1.9(3)
C29	C46	C47	C58	152.2(2)
C30	C13	C14	C15	-143.4(2)
C30	C13	C14	C32	-0.2(2)
C30	C29	C46	C45	142.2(2)
C30	C29	C46	C47	-3.0(3)
C30	C31	C32	C14	-1.5(2)
C30	C31	C32	C33	145.3(2)
C30	C31	C48	C47	-5.8(3)
C30	C31	C48	C49	-159.0(2)
C31	C32	C33	C34	-138.6(2)
C31	C32	C33	C50	19.0(3)
C31	C48	C49	C50	18.7(3)
C31	C48	C49	C60	-166.57(19)
C32	C14	C15	C3	-139.9(2)
C32	C14	C15	C16	-0.4(3)
C32	C31	C48	C47	136.8(2)
C32	C31	C48	C49	-16.4(3)
C32	C33	C34	C16	-3.9(3)
C32	C33	C34	C35	146.34(19)
C32	C33	C50	C49	-16.9(3)
C32	C33	C50	C51	176.99(17)
C33	C34	C35	C36	-144.91(19)
C33	C34	C35	C52	11.6(3)
C33	C50	C51	C52	55.7(2)
C33	C50	C51	C62	176.63(17)
C33	C50	C51	C83	-65.8(2)
C34	C16	C17	C4	-142.1(2)
C34	C16	C17	C18	1.3(3)
C34	C33	C50	C49	140.5(2)
C34	C33	C50	C51	-25.6(3)
C34	C35	C36	C18	-1.0(3)
C34	C35	C36	C37	137.7(2)
C34	C35	C52	C51	23.4(3)
C34	C35	C52	C53	-142.8(2)
C35	C36	C37	C38	-139.6(2)
C35	C36	C37	C54	5.3(3)
C35	C52	C53	C54	6.6(3)
C35	C52	C53	C63	166.8(2)
C36	C18	C19	C20	-142.5(2)
C36	C18	C19	C38	-0.2(2)
C36	C35	C52	C51	179.40(18)
C36	C35	C52	C53	13.2(3)
C36	C37	C38	C19	-5.9(2)
C36	C37	C38	C39	132.5(2)

Atom	Atom	Atom	Atom	Angle/°
C36	C37	C54	C53	15.7(3)
C36	C37	C54	C55	-140.5(2)
C37	C38	C39	C22	-140.9(2)
C37	C38	C39	C40	6.1(3)
C37	C54	C55	C40	16.0(3)
C37	C54	C55	C64	-175.8(2)
C38	C19	C20	C5	-139.3(2)
C38	C19	C20	C21	-3.8(3)
C38	C37	C54	C53	157.2(2)
C38	C37	C54	C55	1.0(3)
C38	C39	C40	C41	-143.4(2)
C38	C39	C40	C55	10.8(3)
C39	C22	C23	C24	-149.8(2)
C39	C22	C23	C41	-0.9(2)
C39	C40	C41	C23	-6.0(2)
C39	C40	C41	C42	142.5(2)
C39	C40	C55	C54	-20.7(3)
C39	C40	C55	C64	176.5(2)
C40	C41	C42	C43	-140.1(2)
C40	C41	C42	C56	12.6(4)
C40	C55	C64	P1	-56.8(3)
C40	C55	C64	C63	153.7(2)
C41	C23	C24	C8	-139.2(2)
C41	C23	C24	C25	-0.8(3)
C41	C40	C55	C54	125.9(2)
C41	C40	C55	C64	-36.9(4)
C41	C42	C43	C25	-7.2(3)
C41	C42	C43	C44	140.7(2)
C41	C42	C56	O1	49.7(3)
C41	C42	C56	C57	-131.6(2)
C42	C43	C44	C45	-144.9(2)
C42	C43	C44	C57	5.5(2)
C42	C56	C57	C44	-20.8(2)
C42	C56	C57	C58	131.2(2)
C43	C25	C26	C9	-141.2(2)
C43	C25	C26	C27	1.4(3)
C43	C42	C56	O1	-154.7(2)
C43	C42	C56	C57	24.0(2)
C43	C44	C45	C27	-2.7(3)
C43	C44	C45	C46	134.7(2)
C43	C44	C57	C56	10.1(2)
C43	C44	C57	C58	-144.7(2)
C44	C45	C46	C29	-142.3(2)
C44	C45	C46	C47	4.0(3)
C44	C57	C58	C47	10.2(3)
C44	C57	C58	C59	177.59(19)
C45	C27	C28	C11	-142.5(2)
C45	C27	C28	C29	-0.7(3)
C45	C44	C57	C56	160.1(2)
C45	C44	C57	C58	5.3(3)
C45	C46	C47	C48	-138.9(2)
C45	C46	C47	C58	11.3(3)
C46	C29	C30	C13	-138.5(2)
C46	C29	C30	C31	-0.3(3)
C46	C47	C48	C31	2.4(3)
C46	C47	C48	C49	155.3(2)
C46	C47	C58	C57	-18.4(3)
C46	C47	C58	C59	173.49(19)
C47	C48	C49	C50	-134.5(2)
C47	C48	C49	C60	40.3(3)
C47	C58	C59	O3	-157.5(2)
C47	C58	C59	C60	23.0(3)
C48	C31	C32	C14	-149.0(2)

Atom	Atom	Atom	Atom	Angle/°
C48	C31	C32	C33	-2.3(3)
C48	C47	C58	C57	132.3(2)
C48	C47	C58	C59	-35.8(3)
C48	C49	C50	C33	-1.9(3)
C48	C49	C50	C51	163.50(19)
C48	C49	C60	C59	-50.1(2)
C48	C49	C60	C61	-170.54(18)
C48	C49	C60	C92	70.4(2)
C49	C50	C51	C52	-110.1(2)
C49	C50	C51	C62	10.8(3)
C49	C50	C51	C83	128.4(2)
C49	C60	C61	C62	1.7(3)
C49	C60	C92	N2	-110.5(2)
C49	C60	C92	C93	65.1(2)
C50	C33	C34	C16	-160.90(19)
C50	C33	C34	C35	-10.7(3)
C50	C49	C60	C59	124.4(2)
C50	C49	C60	C61	4.0(3)
C50	C49	C60	C92	-115.1(2)
C50	C51	C52	C35	-53.9(2)
C50	C51	C52	C53	111.5(2)
C50	C51	C62	C61	-5.5(3)
C50	C51	C83	N1	-55.4(2)
C50	C51	C83	C84	132.5(2)
C51	C52	C53	C54	-158.33(19)
C51	C52	C53	C63	1.9(4)
C51	C83	C84	C85	170.3(2)
C52	C35	C36	C18	-157.94(19)
C52	C35	C36	C37	-19.3(3)
C52	C51	C62	C61	110.1(2)
C52	C51	C83	N1	-171.82(17)
C52	C51	C83	C84	16.1(3)
C52	C53	C54	C37	-21.6(3)
C52	C53	C54	C55	137.65(19)
C52	C53	C63	O2	45.1(4)
C52	C53	C63	C64	-140.6(2)
C53	C54	C55	C40	-142.74(19)
C53	C54	C55	C64	25.5(2)
C53	C63	C64	P1	-157.37(15)
C53	C63	C64	C55	-7.0(2)
C54	C37	C38	C19	-150.4(2)
C54	C37	C38	C39	-12.0(3)
C54	C53	C63	O2	-152.7(2)
C54	C53	C63	C64	21.6(2)
C54	C55	C64	P1	139.34(16)
C54	C55	C64	C63	-10.2(2)
C55	C40	C41	C23	-155.4(2)
C55	C40	C41	C42	-6.9(4)
C56	C42	C43	C25	-166.4(2)
C56	C42	C43	C44	-18.5(2)
C56	C57	C58	C47	-138.1(2)
C56	C57	C58	C59	29.3(3)
C57	C44	C45	C27	-149.9(2)
C57	C44	C45	C46	-12.5(3)
C57	C58	C59	O3	35.0(3)
C57	C58	C59	C60	-144.5(2)
C58	C47	C48	C31	-148.6(2)
C58	C47	C48	C49	4.3(3)
C58	C59	C60	C49	19.0(2)
C58	C59	C60	C61	141.93(18)
C58	C59	C60	C92	-97.9(2)
C59	C60	C61	C62	-119.9(2)
C59	C60	C92	N2	9.1(3)

Atom	Atom	Atom	Atom	Angle/°
C59	C60	C92	C93	-175.2(2)
C60	C49	C50	C33	-176.13(19)
C60	C49	C50	C51	-10.7(3)
C60	C61	C62	C51	-0.4(3)
C60	C92	C93	C94	-176.9(2)
C61	C60	C92	N2	128.4(2)
C61	C60	C92	C93	-55.9(3)
C62	C51	C52	C35	-174.32(17)
C62	C51	C52	C53	-8.9(3)
C62	C51	C83	N1	66.1(2)
C62	C51	C83	C84	-106.0(2)
C63	C53	C54	C37	171.73(19)
C63	C53	C54	C55	-29.0(2)
C64	P1	C65	C66	26.1(2)
C64	P1	C65	C70	-160.60(17)
C64	P1	C71	C72	-148.13(19)
C64	P1	C71	C76	28.3(2)
C64	P1	C77	C78	-84.78(19)
C64	P1	C77	C82	89.44(19)
C65	P1	C64	C55	141.16(17)
C65	P1	C64	C63	-72.5(2)
C65	P1	C71	C72	-20.6(2)
C65	P1	C71	C76	155.83(18)
C65	P1	C77	C78	144.50(17)
C65	P1	C77	C82	-41.3(2)
C65	C66	C67	C68	0.2(3)
C66	C65	C70	C69	0.8(3)
C66	C67	C68	C69	1.1(3)
C67	C68	C69	C70	-1.4(3)
C68	C69	C70	C65	0.5(3)
C70	C65	C66	C67	-1.1(3)
C71	P1	C64	C55	-100.60(19)
C71	P1	C64	C63	45.76(19)
C71	P1	C65	C66	-92.89(19)
C71	P1	C65	C70	80.43(19)
C71	P1	C77	C78	30.6(2)
C71	P1	C77	C82	-155.22(17)
C71	C72	C73	C74	-0.3(4)
C72	C71	C76	C75	0.6(3)
C72	C73	C74	C75	0.8(4)
C73	C74	C75	C76	-0.7(4)
C74	C75	C76	C71	-0.1(4)
C76	C71	C72	C73	-0.5(4)
C77	P1	C64	C55	17.0(2)
C77	P1	C64	C63	163.34(16)
C77	P1	C65	C66	150.01(17)
C77	P1	C65	C70	-36.7(2)
C77	P1	C71	C72	95.6(2)
C77	P1	C71	C76	-88.0(2)
C77	C78	C79	C80	1.4(3)
C78	C77	C82	C81	0.4(3)
C78	C79	C80	C81	-0.4(4)
C79	C80	C81	C82	-0.7(4)
C80	C81	C82	C77	0.7(3)
C82	C77	C78	C79	-1.4(3)
C83	N1	C87	C86	0.3(3)
C83	N1	C87	C88	177.2(2)
C83	C51	C52	C35	68.0(2)
C83	C51	C52	C53	-126.6(2)
C83	C51	C62	C61	-127.7(2)
C83	C84	C85	C86	-0.1(3)
C84	C85	C86	C87	1.4(3)
C85	C86	C87	N1	-1.6(3)

Atom	Atom	Atom	Atom	Angle/°
C85	C86	C87	C88	-178.2(2)
C86	C87	C88	C89	-10.2(4)
C86	C87	C88	C90	109.1(3)
C86	C87	C88	C91	-130.7(2)
C87	N1	C83	C51	-170.99(18)
C87	N1	C83	C84	1.1(3)
C92	N2	C96	C95	-0.5(3)
C92	N2	C96	C97	-174.7(2)
C92	C60	C61	C62	118.6(2)
C92	C93	C94	C95	0.2(4)
C93	C94	C95	C96	0.7(4)
C94	C95	C96	N2	-0.6(4)
C94	C95	C96	C97	173.1(2)
C95	C96	C97	C98	24.6(3)
C95	C96	C97	C99	-92.8(3)
C95	C96	C97	C100	148.1(3)
C96	N2	C92	C60	176.92(19)
C96	N2	C92	C93	1.6(3)

Table S3.1.6 Hydrogen Fractional Atomic Coordinates (×10[4]) and Equivalent Isotropic Displacement Parameters (Å^2×10^3) for open fullerene **4**. U_{eq} is defined as 1/3 of the trace of the orthogonalised U_{ij}.

Atom	x	y	z	U_{eq}
H61	3975.17	2401.27	6206.76	21
H62	3927.16	1887.03	7252.03	19
H66	3239.57	655.66	5413.63	24
H67	3754.42	562.83	4468.79	30
H68	3524.41	-613.38	3635.63	32
H69	2759.14	-1672.92	3720.66	30
H70	2245.84	-1599.74	4667.96	26
H72	3055.79	-2191.43	5617.81	30
H73	3585.61	-3340.8	6280.42	37
H74	3593.07	-3311.53	7458.13	37
H75	3050.68	-2153.4	7965.73	37
H76	2514.56	-1003.37	7311.05	30
H78	1571.69	-1766.81	6507.14	26
H79	594.29	-2080.94	6303.19	30
H80	68.34	-1442.06	5346.19	30
H81	516	-465	4592.71	30
H82	1489.42	-102.15	4807.29	26
H84	3473.42	2066.51	8872.92	25
H85	4231.41	2252.97	9732.8	29
H86	4961.33	3367.34	9577.5	29
H89A	5320.63	5040.03	9484.69	70
H89B	5810.24	5203.58	8991.55	70
H89C	5688.07	4130.56	9264.43	70
H90A	5566.6	3529.87	8068	47
H90B	5690.36	4612.59	7814.14	47
H90C	5118.58	4055.83	7524.25	47
H91A	4514.46	5461.05	7906.64	57
H91B	5096.78	6012.23	8163.59	57
H91C	4620.61	5836.76	8672.75	57
H93	4267.79	4458.43	6922.37	34
H94	4895.3	5701.58	6652.04	39
H95	4809.97	6323.43	5550.73	39
H98A	4631.87	7020.66	4575.98	64
H98B	4450.01	6706.58	3807.51	64
H98C	4864.55	6019.12	4289.84	64
H99A	3201.73	6345.28	4686.01	61
H99B	3449.89	6997.72	4113.68	61
H99C	3672.6	7165.25	4894.09	61
H10A	4111.79	4658.16	3941.33	52

Atom	x	y	z	U_{eq}
H10B	3731.34	5465.42	3521.9	52
H10C	3464.91	4839.85	4094.74	52

Table S3.1.7 Solvent masking (Olex2) information for open fullerene **4**.

No	x	y	z	V	e	Content
1	0.170	0.643	0.375	130.8	68.9	chloroform
2	0.170	0.857	0.875	130.8	68.9	chloroform
3	0.154	0.155	0.183	29.8	0.0	?
4	0.154	0.345	0.683	29.8	0.0	?
5	0.490	0.211	0.773	8.0	0.0	?
6	0.490	0.289	0.273	8.0	0.0	?
7	0.510	0.711	0.727	8.0	0.0	?
8	0.510	0.789	0.227	8.0	0.0	?
9	0.830	0.143	0.125	130.8	67.8	chloroform
10	0.830	0.357	0.625	130.8	67.8	chloroform
11	0.846	0.655	0.317	29.8	0.0	?
12	0.846	0.845	0.817	29.8	0.0	?

S3.2 X-ray structure determination of Ne@C_{60}

S3.2.1 Experimental method

Preparation of single dark orange crystals was made by slow evaporation of the nickel(II) octaethylporphyrin/benzene solvate of a sample of Ne@C_{60} with >99.5% neon filling.[15] A suitable crystal 0.44×0.27×0.10 mm^3 was selected and mounted on a MITIGEN holder with silicon oil on an Rigaku AFC12 FRE-VHF diffractometer. The crystal was kept at a steady T = 100(2) K during data collection. The structure was solved with the ShelXT[13a] structure solution program using the Intrinsic Phasing solution method and by using Olex2[14] as the graphical interface. The model was refined with version 2016/6 of ShelXL[13b] using Least Squares minimisation.

S3.2.2 Crystal data

$C_{108}H_{56}N_4NeNi$, M_r = 1488.45, triclinic, P-1 (No. 2), a = 14.09380(10) Å, b = 14.37270(10) Å, c = 17.1780(2) Å, α = 87.5290(10)°, β = 75.7750(10)°, γ = 75.6020(10)°, V = 3266.45(5) Å^3, T = 100(2) K, Z = 2, Z' = 1, □(MoKα) = 0.368, 160930 reflections measured, 16553 unique (R_{int} = 0.0388) which were used in all calculations. The final wR_2 was 0.0908 (all data) and R_1 was 0.0327 (I > 2(I)).

CCDC	1953465	Z	2
Formula	$C_{108}H_{56}N_4NeNi$	Z'	1
$D_{calc.}$/ g cm^{-3}	1.513	Wavelength/Å	0.71073
μ/mm^{-1}	0.368	Radiation type	MoKα
Formula Weight	1488.45	Θ_{min}/°	1.844
Colour	dark orange	Θ_{max}/°	28.499

Shape	plate	Measured Refl.	160930
Size/mm^3	0.44×0.27×0.10	Independent Refl.	16553
T/K	100(2)	Reflections with I > 2(I)	15199
Crystal System	triclinic	R_{int}	0.0388
Space Group	P-1	Parameters	1035
a/Å	14.09380(10)	Restraints	0
b/Å	14.37270(10)	Largest Peak	0.382
c/Å	17.1780(2)	Deepest Hole	-0.501
α/°	87.5290(10)	GooF	1.044
β/°	75.7750(10)	wR_2 (all data)	0.0908
γ/°	75.6020(10)	wR_2	0.0889
V/Å^3	3266.45(5)	R_1 (all data)	0.0360
Formula	$C_{108}H_{56}N_4NeNi$	R_1	0.0327

S3.2.3 Structure Quality Indicators

**Reflections:**	lo) 0.74	I/σ 58.5	Rint 3.88%	complete 100% (IUCr) 100%
**Refinement:**	0.001	Max Peak 0.4	Min Peak -0.5	GooF 1.044

A dark orange plate-shaped crystal with dimensions 0.44×0.27×0.10 mm^3 was mounted on a MITIGEN holder with silicon oil. X-ray diffraction data were collected using a Rigaku AFC12 FRE-VHF diffractometer equipped with an Oxford Cryosystems low-temperature device, operating at T = 100(2) K.

Data were measured using profile data from □-scans of 0.5 ° per frame for 6.0 s using MoK$_\alpha$ radiation (Rotating-anode X-ray tube, 45.0 kV, 55.0 mA). The total number of runs and images was based on the strategy calculation from the program CrysAlisPro (Rigaku, V1.171.40.37a, 2019). The maximum resolution achieved was Θ = 28.499°.

Cell parameters were retrieved using the CrysAlisPro (Rigaku, V1.171.40.37a, 2019) software and refined using CrysAlisPro (Rigaku, V1.171.40.37a, 2019) on 105286 reflections, 65 % of the observed reflections. Data reduction was performed using the CrysAlisPro (Rigaku, V1.171.40.37a, 2019) software that corrects for Lorentz polarisation. The final completeness is 99.90 % out to 28.499° in Θ.

A multi-scan absorption correction was performed using CrysAlisPro 1.171.40.37a (Rigaku Oxford Diffraction, 2019) using spherical harmonicsas implemented in SCALE3 ABSPACK. The absorption coefficient □ of this material is 0.368 mm^{-1} at this wavelength (λ = 0.711Å) and the minimum and maximum transmissions are 0.723 and 1.000

The structure was solved in the space group P-1 (# 2) by Intrinsic Phasing using the ShelXT[13a] structure solution program and refined by Least Squares using version 2016/6 of ShelXL.[13b] All non-hydrogen atoms were refined anisotropically. Hydrogen atom positions were calculated geometrically and refined using the riding model.

_exptl_absorpt_process_details: CrysAlisPro 1.171.40.37a (Rigaku Oxford Diffraction, 2019) using spherical harmonicsas implemented in SCALE3 ABSPACK.

There is a single molecule in the asymmetric unit, which is represented by the reported sum formula. In other words: Z is 2 and Z' is 1.

S3.2.4 Data Plots: Diffraction Data

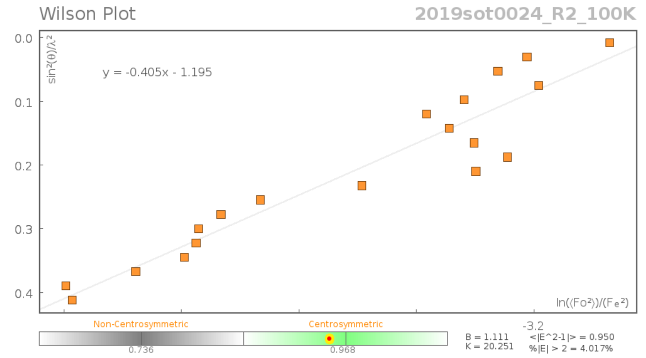

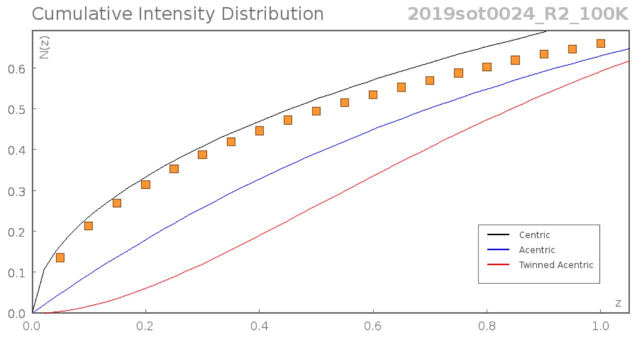

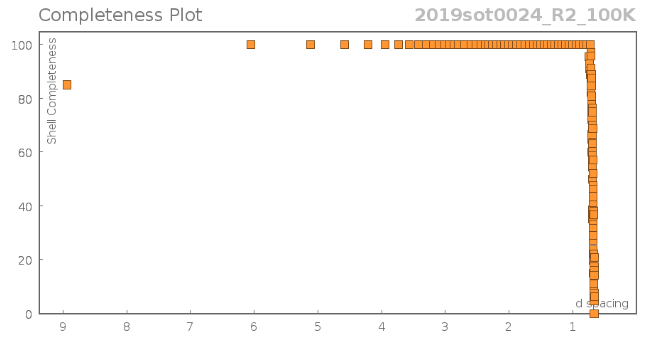

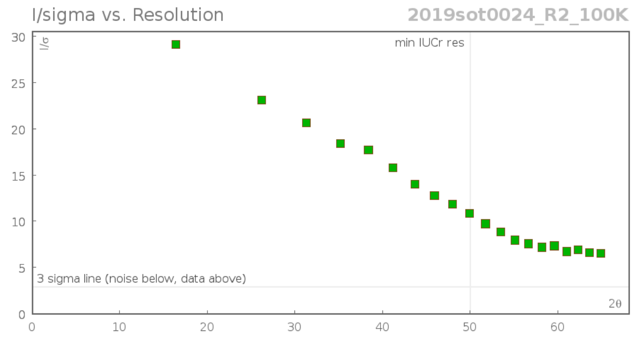

S3.2.5 Data Plots: Refinement and Data

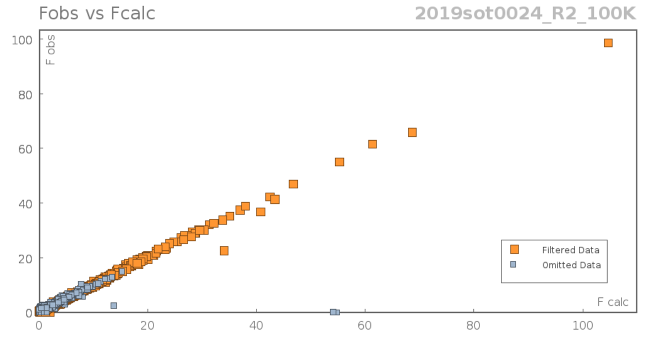

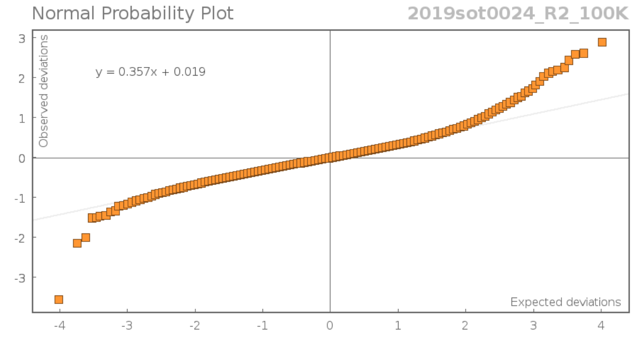

S3.2.6 Reflection Statistics

Total reflections (after filtering)	160945	Unique reflections	16553
Completeness	1.0	Mean I/□	44.08
hkl$_{max}$ collected	(20, 20, 25)	hkl$_{min}$ collected	(-21, -21, -25)
hkl$_{max}$ used	(18, 19, 23)	hkl$_{min}$ used	(-18, -19, 0)
Lim d$_{max}$ collected	100.0	Lim d$_{min}$ collected	0.74
d$_{max}$ used	11.86	d$_{min}$ used	0.74
Friedel pairs	18921	Friedel pairs merged	1
Inconsistent equivalents	15	R$_{int}$	0.0388
R$_{sigma}$	0.0171	Intensity transformed	0

Omitted reflections	0	Omitted by user (OMIT hkl)	15
Multiplicity	(5592, 5865, 6088, 6420, 4710, 4027, 3061, 1978, 1325, 884, 383, 128, 36, 9)	Maximum multiplicity	21
Removed systematic absences	0	Filtered off (Shel/OMIT)	12392

S3.2.7 Images of the Crystal on the Diffractometer

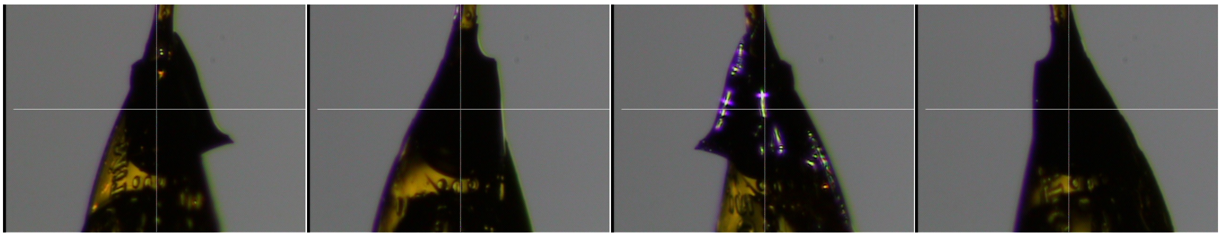

Table S3.2.1 Fractional Atomic Coordinates (×10^4) and Equivalent Isotropic Displacement Parameters (Å^2×10^3) for Ne@C$_{60}$. U_{eq} is defined as 1/3 of the trace of the orthogonalised U_{ij}.

Atom	x	y	z	U_{eq}
Ni1	4656.9(2)	5862.2(2)	8873.2(2)	8.40(4)
N1	4164.4(7)	5252.2(7)	8114.4(5)	11.17(17)
N2	3484.6(7)	6947.7(6)	8995.9(5)	10.61(17)
N3	5157.0(7)	6483.7(6)	9621.6(5)	9.97(16)
N4	5839.0(7)	4781.7(6)	8738.4(5)	10.14(16)
C62	4670.1(8)	4456.4(8)	7628.4(6)	12.4(2)
C63	4058.7(9)	4231.7(8)	7134.7(7)	13.7(2)
C64	3147.4(9)	4879.2(8)	7348.1(7)	14.2(2)
C65	3229.7(8)	5517.7(8)	7940.5(7)	12.6(2)
C66	2496.0(8)	6335.0(8)	8244.0(7)	13.5(2)
C67	2636.6(8)	7020.6(8)	8711.8(6)	12.0(2)
C68	1928.0(8)	7943.1(8)	8925.2(7)	12.9(2)
C69	2369.8(8)	8452.7(8)	9313.8(7)	12.8(2)
C70	3321.3(8)	7820.4(8)	9372.7(6)	11.38(19)
C71	3942.0(8)	8044.5(8)	9806.0(6)	11.91(19)
C72	4784.4(8)	7402.5(8)	9946.7(6)	10.87(19)
C73	5379.3(8)	7602.0(8)	10465.9(6)	11.62(19)
C74	6119.9(8)	6786.6(8)	10465.6(6)	11.45(19)
C75	5973.8(8)	6106.5(8)	9938.2(6)	10.39(19)
C76	6620.2(8)	5209.1(8)	9746.9(6)	11.27(19)
C77	6576.2(8)	4611.4(7)	9158.9(6)	10.55(19)
C78	7352.6(8)	3749.2(8)	8869.0(7)	11.79(19)
C79	7087.0(8)	3392.7(8)	8256.4(7)	13.0(2)
C80	6145.1(8)	4034.0(8)	8184.5(6)	11.36(19)
C81	5612.9(8)	3897.4(8)	7643.4(7)	13.2(2)
C82	4391.4(9)	3437.9(9)	6516.7(7)	17.6(2)
C83	4216.9(12)	2478.0(9)	6842.3(8)	26.6(3)
C84	2226.7(9)	4946.1(9)	7036.9(7)	18.0(2)
C85	1450.9(11)	4491.8(11)	7590.1(10)	30.4(3)
C86	900.3(8)	8225.6(8)	8764.7(7)	16.3(2)
C87	132.5(9)	7804.7(10)	9369.8(8)	22.2(2)
C88	1958.5(9)	9458.0(8)	9656.2(7)	16.5(2)
C89	1403.0(11)	9495.2(10)	10541.6(8)	25.7(3)
C90	5208.8(9)	8534.0(8)	10904.2(7)	14.7(2)
C91	5920.0(12)	9144.1(10)	10495.8(9)	31.0(3)
C92	6963.8(8)	6609.4(8)	10881.6(7)	14.0(2)
C93	7969.9(9)	6707.7(9)	10331.6(7)	19.4(2)

Atom	x	y	z	U_{eq}
C94	8278.3(8)	3385.4(8)	9179.4(7)	14.4(2)
C95	9091.5(9)	3935.4(9)	8856.2(8)	18.7(2)
C96	7644.2(9)	2518.8(8)	7739.0(7)	17.5(2)
C97	8454.3(11)	2714.9(10)	7026.7(8)	27.0(3)
C1	6529.6(11)	10245.6(9)	5698.1(9)	26.1(3)
C2	7192.9(11)	10493.3(8)	6138.4(8)	24.1(3)
C3	8231.0(11)	10206.2(9)	5841.3(8)	23.8(3)
C4	8653.7(11)	9658.7(10)	5094.0(8)	26.5(3)
C5	8019.0(12)	9417.9(11)	4674.6(8)	28.0(3)
C6	6934.7(12)	9721.8(10)	4982.6(8)	27.7(3)
C7	6508.2(12)	8953.1(11)	4811.0(8)	27.5(3)
C8	5693.5(11)	8746.4(10)	5365.9(8)	25.7(3)
C9	5270.3(10)	9294.8(10)	6113.9(9)	24.7(3)
C10	5679.2(10)	10028.5(9)	6275.9(9)	25.0(3)
C11	5817.3(10)	10139.4(9)	7073.4(8)	23.2(3)
C12	6752.0(10)	10429.9(8)	6988.8(8)	22.5(3)
C13	7369.3(10)	10080.2(8)	7506.1(8)	20.7(2)
C14	8453.3(10)	9779.5(9)	7198.1(8)	20.6(2)
C15	8876.4(10)	9840.5(9)	6382.3(8)	21.8(2)
C16	9695.0(10)	9066.3(10)	5969.5(8)	24.0(3)
C17	9559.7(11)	8951.3(11)	5173.0(8)	26.6(3)
C18	9788.7(11)	8038.0(11)	4834.1(8)	29.4(3)
C19	9129.1(12)	7789.3(12)	4394.1(8)	29.9(3)
C20	8265.9(12)	8463.0(11)	4314.9(7)	29.6(3)
C21	7331.1(12)	8178.7(11)	4401.3(7)	28.3(3)
C22	7304.4(12)	7229.3(11)	4562.8(7)	27.1(3)
C23	6449.4(11)	7015.1(10)	5141.7(8)	24.1(3)
C24	5664.6(10)	7755.0(10)	5534.1(8)	23.5(3)
C25	5224.2(9)	7690.8(10)	6385.2(8)	21.3(2)
C26	4979.3(9)	8643.0(10)	6743.4(8)	22.2(3)
C27	5110.6(9)	8748.3(9)	7507.3(8)	20.8(2)
C28	5541.2(9)	9514.7(9)	7672.9(8)	21.6(2)
C29	6182.9(10)	9150.8(9)	8214.1(7)	19.9(2)
C30	7077.5(10)	9428.4(9)	8134.6(7)	19.8(2)
C31	7976.9(10)	8726.9(9)	8216.1(7)	19.3(2)
C32	8829.4(10)	8942.6(9)	7636.0(7)	20.2(2)
C33	9610.4(9)	8202.1(10)	7242.2(8)	22.1(3)
C34	10053.8(9)	8262.5(10)	6391.1(8)	24.0(3)
C35	10294.5(9)	7311.7(11)	6036.4(9)	27.1(3)
C36	10166.2(10)	7202.6(11)	5273.7(9)	29.3(3)
C37	9739.0(10)	6436.3(11)	5105.4(9)	30.2(3)
C38	9103.1(11)	6796.7(11)	4559.7(8)	30.4(3)
C39	8203.8(12)	6523.7(11)	4642.1(8)	28.4(3)
C40	7911.9(11)	5871.7(9)	5272.5(8)	26.1(3)
C41	6824.8(11)	6177.0(9)	5579.5(8)	23.1(3)
C42	6401.9(10)	6115.9(9)	6393.9(8)	20.6(2)
C43	5581.7(9)	6890.5(9)	6806.7(8)	19.4(2)
C44	5718.5(9)	7001.8(9)	7603.1(7)	18.9(2)
C45	5488.5(9)	7911.7(9)	7944.1(7)	19.3(2)
C46	6149.5(9)	8160.3(9)	8382.7(7)	19.1(2)
C47	7013.5(10)	7489.6(9)	8461.4(7)	19.1(2)
C48	7944.9(10)	7776.8(9)	8372.6(7)	19.8(2)
C49	8765.8(10)	7002.6(9)	7963.8(8)	21.7(2)
C50	9581.1(10)	7209.2(10)	7412.0(8)	23.6(3)
C51	10005.3(9)	6661.0(10)	6664.7(9)	26.3(3)
C52	9593.4(10)	5927.6(9)	6503.0(9)	27.3(3)
C53	9458.7(10)	5814.8(10)	5710.2(9)	28.6(3)
C54	8523.3(11)	5525.2(9)	5791.8(9)	26.5(3)
C55	8084.5(11)	5458.1(8)	6640.9(9)	24.2(3)
C56	7044.0(10)	5750.0(8)	6936.0(8)	20.8(2)
C57	6624.9(10)	6298.8(9)	7681.0(7)	19.1(2)
C58	7257.6(10)	6535.1(9)	8102.7(7)	19.6(2)
C59	8338.4(10)	6235.4(9)	7795.5(8)	22.5(3)

**Atom**	**x**	**y**	**z**	U_{eq}
C60	8744.2(10)	5707.6(9)	7081.1(9)	24.5(3)
Ne1	7639.5(6)	7980.3(6)	6386.6(5)	43.03(19)
C98	3122.3(10)	6320.4(10)	4658.9(9)	24.8(3)
C99	2625.0(10)	7282.4(10)	4794.5(9)	24.6(3)
C100	2654.3(10)	7762.7(10)	5466.3(9)	27.4(3)
C101	3190.4(11)	7286.8(11)	6000.8(9)	29.1(3)
C102	3715.2(12)	6331.5(11)	5854.4(10)	30.6(3)
C103	3670.5(11)	5847.6(10)	5188.8(10)	29.5(3)
C104	2342.1(11)	9893.4(10)	7312.6(9)	29.7(3)
C105	1350.4(11)	9979.6(10)	7302.2(8)	26.2(3)
C106	623.1(11)	10792.8(12)	7628.3(9)	31.4(3)
C107	892.3(12)	11515.9(11)	7964.0(10)	36.4(4)
C108	1886.0(13)	11427.9(11)	7974.5(10)	32.7(3)
C109	2613.7(11)	10614.7(11)	7645.4(10)	32.1(3)

Table S3.2.2 Anisotropic Displacement Parameters (×10^4) Ne@C_{60}. The anisotropic displacement factor exponent takes the form: $-2□^2[h^2a^{*2} \times U_{11}+ ... +2hka^* \times b^* \times U_{12}]$

**Atom**	U_{11}	U_{22}	U_{33}	U_{23}	U_{13}	U_{12}
Ni1	7.42(7)	8.47(7)	9.62(7)	-0.54(5)	-2.44(5)	-2.04(5)
N1	10.3(4)	11.3(4)	12.4(4)	0.6(3)	-3.2(3)	-3.0(3)
N2	9.4(4)	10.9(4)	12.0(4)	0.2(3)	-2.9(3)	-3.1(3)
N3	8.8(4)	10.2(4)	10.6(4)	0.3(3)	-1.7(3)	-2.5(3)
N4	9.8(4)	9.8(4)	11.1(4)	0.2(3)	-2.0(3)	-3.6(3)
C62	14.4(5)	13.3(5)	10.9(5)	-0.2(4)	-3.2(4)	-5.8(4)
C63	15.8(5)	15.6(5)	12.3(5)	0.0(4)	-5.3(4)	-6.3(4)
C64	15.5(5)	15.5(5)	14.0(5)	1.0(4)	-6.3(4)	-5.9(4)
C65	12.9(5)	13.7(5)	13.4(5)	1.9(4)	-5.4(4)	-5.3(4)
C66	11.4(5)	15.8(5)	14.7(5)	1.6(4)	-5.9(4)	-3.5(4)
C67	10.0(5)	12.8(5)	12.5(5)	2.2(4)	-2.7(4)	-1.8(4)
C68	11.1(5)	12.6(5)	14.1(5)	2.3(4)	-3.5(4)	-1.0(4)
C69	10.4(5)	11.4(5)	15.4(5)	2.0(4)	-2.5(4)	-1.4(4)
C70	9.7(5)	10.5(5)	13.1(5)	1.3(4)	-1.3(4)	-2.5(4)
C71	11.2(5)	9.8(5)	14.2(5)	-1.5(4)	-1.2(4)	-3.3(4)
C72	10.2(5)	11.7(5)	10.3(5)	-1.0(4)	0.0(4)	-4.3(4)
C73	11.0(5)	13.9(5)	10.3(5)	-1.6(4)	-0.9(4)	-5.0(4)
C74	11.5(5)	14.0(5)	9.5(4)	-0.2(4)	-1.5(4)	-5.3(4)
C75	9.7(4)	12.6(5)	9.4(4)	0.6(4)	-1.9(4)	-4.3(4)
C76	9.5(4)	12.8(5)	11.9(5)	2.4(4)	-3.5(4)	-3.2(4)
C77	9.0(4)	9.9(4)	12.4(5)	2.0(4)	-1.8(4)	-2.7(4)
C78	9.6(5)	10.1(5)	14.5(5)	1.9(4)	-1.2(4)	-2.2(4)
C79	10.9(5)	10.3(5)	16.4(5)	0.1(4)	-1.1(4)	-2.3(4)
C80	9.9(5)	10.7(5)	12.9(5)	-0.1(4)	-0.8(4)	-3.5(4)
C81	13.1(5)	12.7(5)	13.4(5)	-3.0(4)	-1.1(4)	-4.0(4)
C82	18.5(5)	20.4(6)	15.3(5)	-5.2(4)	-5.7(4)	-5.0(4)
C83	42.8(8)	14.0(6)	23.5(6)	-3.3(5)	-14.0(6)	-1.4(5)
C84	17.6(5)	19.9(5)	20.3(6)	-0.7(4)	-10.5(4)	-5.8(4)
C85	21.6(6)	36.2(8)	41.4(8)	13.5(6)	-16.8(6)	-14.6(6)
C86	12.7(5)	15.9(5)	20.7(5)	2.3(4)	-7.9(4)	-0.8(4)
C87	13.4(5)	26.0(6)	28.7(6)	4.9(5)	-7.8(5)	-5.6(5)
C88	13.7(5)	10.3(5)	24.8(6)	-0.5(4)	-5.6(4)	-0.6(4)
C89	27.7(7)	19.2(6)	25.4(6)	-7.0(5)	-1.8(5)	-0.5(5)
C90	15.3(5)	14.5(5)	14.4(5)	-4.4(4)	-2.9(4)	-3.9(4)
C91	35.5(8)	19.8(6)	33.3(7)	-11.2(5)	9.7(6)	-14.6(6)
C92	14.4(5)	17.0(5)	12.5(5)	-0.2(4)	-5.5(4)	-4.8(4)
C93	13.0(5)	26.5(6)	20.5(6)	1.6(5)	-6.0(4)	-6.7(4)
C94	12.0(5)	12.6(5)	18.0(5)	2.2(4)	-4.7(4)	-1.3(4)
C95	12.6(5)	20.0(6)	24.5(6)	2.2(4)	-6.0(4)	-4.5(4)
C96	16.5(5)	12.8(5)	21.5(6)	-4.8(4)	-4.4(4)	0.3(4)
C97	26.5(7)	28.1(7)	19.7(6)	-7.7(5)	1.0(5)	0.4(5)
C1	35.1(7)	14.3(5)	33.7(7)	12.4(5)	-19.6(6)	-5.6(5)
C2	36.9(7)	9.2(5)	29.8(7)	7.4(5)	-14.0(6)	-7.7(5)
C3	37.2(7)	16.3(6)	24.7(6)	8.8(5)	-9.8(5)	-17.9(5)

Atom	U_{11}	U_{22}	U_{33}	U_{23}	U_{13}	U_{12}
C4	37.3(7)	27.8(7)	19.8(6)	11.4(5)	-4.3(5)	-22.5(6)
C5	43.8(8)	31.2(7)	15.4(6)	13.8(5)	-8.7(5)	-21.7(6)
C6	42.1(8)	25.7(7)	23.2(6)	16.6(5)	-19.0(6)	-14.7(6)
C7	40.7(8)	32.1(7)	19.8(6)	12.0(5)	-22.0(6)	-15.0(6)
C8	28.5(7)	30.1(7)	27.2(7)	7.6(5)	-22.2(6)	-8.8(5)
C9	17.7(6)	24.4(6)	33.3(7)	5.8(5)	-15.8(5)	1.1(5)
C10	23.5(6)	14.5(5)	35.9(7)	4.9(5)	-15.3(5)	4.4(5)
C11	21.1(6)	11.1(5)	32.6(7)	-4.6(5)	-6.6(5)	5.7(4)
C12	29.6(7)	7.2(5)	29.7(7)	-2.9(4)	-7.6(5)	-1.8(4)
C13	30.0(7)	11.9(5)	21.7(6)	-6.3(4)	-6.3(5)	-7.1(5)
C14	28.4(6)	17.2(5)	23.2(6)	-1.6(4)	-10.1(5)	-14.3(5)
C15	27.2(6)	19.3(6)	26.2(6)	3.7(5)	-8.0(5)	-17.9(5)
C16	20.0(6)	30.7(7)	26.2(6)	3.1(5)	-1.8(5)	-19.6(5)
C17	26.5(7)	35.3(7)	20.7(6)	4.8(5)	3.2(5)	-22.1(6)
C18	23.8(6)	41.7(8)	19.5(6)	-3.4(5)	10.8(5)	-17.6(6)
C19	34.1(7)	42.2(8)	11.6(5)	-5.2(5)	9.1(5)	-19.6(6)
C20	45.3(8)	41.4(8)	7.4(5)	5.4(5)	-2.5(5)	-25.0(7)
C21	43.1(8)	40.3(8)	9.4(5)	3.8(5)	-10.9(5)	-20.6(6)
C22	40.3(8)	37.2(7)	10.7(5)	-5.3(5)	-7.3(5)	-20.1(6)
C23	33.5(7)	30.7(7)	17.4(6)	-3.0(5)	-12.0(5)	-18.4(6)
C24	24.6(6)	32.5(7)	23.2(6)	3.6(5)	-16.3(5)	-14.9(5)
C25	13.5(5)	29.3(6)	26.3(6)	0.0(5)	-9.1(5)	-10.6(5)
C26	9.1(5)	25.9(6)	31.5(7)	0.3(5)	-7.7(5)	-1.5(4)
C27	8.7(5)	23.6(6)	25.2(6)	-4.4(5)	1.5(4)	-0.1(4)
C28	14.9(5)	19.0(6)	24.4(6)	-9.3(5)	1.0(5)	3.7(4)
C29	21.0(6)	21.2(6)	13.3(5)	-9.0(4)	2.3(4)	-2.1(5)
C30	27.3(6)	18.9(6)	13.4(5)	-8.2(4)	-3.0(4)	-6.7(5)
C31	25.5(6)	25.5(6)	12.0(5)	-1.6(4)	-9.3(4)	-10.7(5)
C32	23.7(6)	24.7(6)	19.9(6)	0.2(5)	-12.2(5)	-13.5(5)
C33	15.7(5)	29.6(7)	28.0(6)	3.9(5)	-12.7(5)	-11.5(5)
C34	11.3(5)	32.8(7)	30.6(7)	0.3(5)	-3.6(5)	-11.9(5)
C35	7.5(5)	32.5(7)	37.3(7)	-3.0(6)	0.8(5)	-3.2(5)
C36	13.8(6)	34.7(7)	31.5(7)	-8.8(6)	10.7(5)	-5.7(5)
C37	19.6(6)	30.8(7)	30.3(7)	-15.9(6)	10.1(5)	-1.4(5)
C38	32.3(7)	35.6(8)	17.0(6)	-14.9(5)	10.8(5)	-11.1(6)
C39	38.6(8)	30.7(7)	15.0(6)	-13.8(5)	2.9(5)	-13.6(6)
C40	35.0(7)	19.1(6)	23.5(6)	-13.7(5)	0.7(5)	-10.4(5)
C41	31.9(7)	20.1(6)	22.9(6)	-7.2(5)	-6.7(5)	-15.2(5)
C42	25.6(6)	16.8(5)	25.0(6)	-1.0(4)	-6.9(5)	-14.6(5)
C43	16.0(5)	24.8(6)	22.5(6)	0.7(5)	-4.2(4)	-14.8(5)
C44	15.6(5)	25.0(6)	17.9(5)	3.9(4)	0.0(4)	-13.0(5)
C45	12.3(5)	27.4(6)	15.6(5)	-0.7(4)	4.1(4)	-7.5(4)
C46	19.9(6)	26.9(6)	9.0(5)	-2.8(4)	3.6(4)	-9.4(5)
C47	26.0(6)	25.0(6)	8.9(5)	5.0(4)	-4.5(4)	-11.4(5)
C48	25.5(6)	26.9(6)	11.8(5)	4.9(4)	-11.0(4)	-9.8(5)
C49	22.8(6)	22.8(6)	24.0(6)	8.9(5)	-16.5(5)	-4.8(5)
C50	15.6(5)	26.2(6)	32.5(7)	5.6(5)	-15.6(5)	-2.9(5)
C51	9.8(5)	25.6(6)	40.3(8)	0.9(5)	-7.5(5)	3.2(5)
C52	16.9(6)	15.8(6)	42.4(8)	-1.2(5)	-5.5(5)	7.3(5)
C53	20.4(6)	17.7(6)	38.4(8)	-11.9(5)	3.1(5)	4.7(5)
C54	28.5(7)	11.3(5)	34.1(7)	-11.1(5)	0.7(5)	-1.0(5)
C55	30.1(7)	6.3(5)	34.5(7)	0.0(4)	-6.8(6)	-2.5(4)
C56	28.2(6)	10.7(5)	26.3(6)	3.2(4)	-5.5(5)	-11.1(5)
C57	24.0(6)	16.3(5)	19.2(6)	7.6(4)	-3.1(5)	-12.0(5)
C58	26.4(6)	18.3(6)	16.8(5)	10.7(4)	-7.2(5)	-10.1(5)
C59	25.5(6)	16.5(6)	27.6(6)	11.9(5)	-13.6(5)	-4.2(5)
C60	23.8(6)	10.5(5)	37.6(7)	6.0(5)	-10.8(5)	1.3(4)
Ne1	43.7(5)	42.9(5)	42.5(5)	0.4(4)	-9.4(4)	-11.7(4)
C98	24.6(6)	22.8(6)	28.6(7)	3.3(5)	-5.8(5)	-10.1(5)
C99	18.0(6)	23.5(6)	34.4(7)	8.8(5)	-8.5(5)	-7.8(5)
C100	16.5(6)	21.6(6)	43.2(8)	-0.9(5)	-2.8(5)	-7.0(5)
C101	28.1(7)	33.1(7)	31.8(7)	0.4(6)	-5.9(6)	-19.4(6)
C102	33.9(7)	28.6(7)	39.9(8)	15.8(6)	-20.2(6)	-18.4(6)

Atom	U_{11}	U_{22}	U_{33}	U_{23}	U_{13}	U_{12}
C103	29.3(7)	16.9(6)	45.5(8)	7.9(6)	-12.9(6)	-8.6(5)
C104	25.4(7)	22.9(6)	35.8(8)	-2.8(5)	-3.7(6)	0.2(5)
C105	32.1(7)	29.6(7)	20.8(6)	4.0(5)	-6.3(5)	-15.2(6)
C106	18.0(6)	41.7(8)	32.9(7)	8.1(6)	-4.6(5)	-7.3(6)
C107	30.0(8)	28.5(7)	39.3(8)	-2.7(6)	1.8(6)	4.1(6)
C108	39.9(8)	26.3(7)	33.7(8)	-3.1(6)	-6.9(6)	-12.9(6)
C109	21.4(6)	33.8(8)	42.5(8)	0.5(6)	-10.4(6)	-6.9(6)

Table S3.2.3 Bond Lengths in Å for Ne@C_{60}.

Atom	Atom	Length/Å	Atom	Atom	Length/Å
Ni1	N1	1.9481(9)	C2	C12	1.4488(19)
Ni1	N2	1.9452(9)	C3	C4	1.449(2)
Ni1	N3	1.9506(9)	C3	C15	1.4501(19)
Ni1	N4	1.9482(9)	C4	C5	1.390(2)
N1	C62	1.3776(14)	C4	C17	1.453(2)
N1	C65	1.3798(14)	C5	C6	1.448(2)
N2	C67	1.3776(14)	C5	C20	1.455(2)
N2	C70	1.3777(14)	C6	C7	1.454(2)
N3	C72	1.3807(13)	C7	C8	1.387(2)
N3	C75	1.3736(13)	C7	C21	1.450(2)
N4	C77	1.3742(14)	C8	C9	1.451(2)
N4	C80	1.3792(13)	C8	C24	1.4503(19)
C62	C63	1.4449(15)	C9	C10	1.388(2)
C62	C81	1.3769(15)	C9	C26	1.4494(19)
C63	C64	1.3615(16)	C10	C11	1.451(2)
C63	C82	1.4974(15)	C11	C12	1.4509(19)
C64	C65	1.4431(15)	C11	C28	1.3839(19)
C64	C84	1.5011(16)	C12	C13	1.3867(19)
C65	C66	1.3802(15)	C13	C14	1.4473(19)
C66	C67	1.3783(16)	C13	C30	1.4486(18)
C67	C68	1.4467(14)	C14	C15	1.3900(18)
C68	C69	1.3576(16)	C14	C32	1.4471(18)
C68	C86	1.4943(15)	C15	C16	1.449(2)
C69	C70	1.4448(14)	C16	C17	1.449(2)
C69	C88	1.5029(15)	C16	C34	1.390(2)
C70	C71	1.3799(15)	C17	C18	1.389(2)
C71	C72	1.3770(15)	C18	C19	1.448(2)
C72	C73	1.4456(15)	C18	C36	1.448(2)
C73	C74	1.3618(15)	C19	C20	1.386(2)
C73	C90	1.5023(15)	C19	C38	1.450(2)
C74	C75	1.4445(15)	C20	C21	1.446(2)
C74	C92	1.4972(15)	C21	C22	1.389(2)
C75	C76	1.3808(15)	C22	C23	1.4508(19)
C76	C77	1.3762(15)	C22	C39	1.445(2)
C77	C78	1.4464(14)	C23	C24	1.385(2)
C78	C79	1.3585(16)	C23	C41	1.448(2)
C78	C94	1.4961(15)	C24	C25	1.4499(19)
C79	C80	1.4454(14)	C25	C26	1.4506(19)
C79	C96	1.4990(15)	C25	C43	1.3858(18)
C80	C81	1.3771(15)	C26	C27	1.3891(19)
C82	C83	1.5203(18)	C27	C28	1.4515(19)
C84	C85	1.5218(18)	C27	C45	1.4467(18)
C86	C87	1.5285(17)	C28	C29	1.4464(18)
C88	C89	1.5264(18)	C29	C30	1.3887(18)
C90	C91	1.5180(17)	C29	C46	1.4504(18)
C92	C93	1.5339(16)	C30	C31	1.4431(18)
C94	C95	1.5320(16)	C31	C32	1.4501(18)
C96	C97	1.5245(18)	C31	C48	1.3899(18)
C1	C2	1.4522(19)	C32	C33	1.3840(19)
C1	C6	1.387(2)	C33	C34	1.4501(19)
C1	C10	1.447(2)	C33	C50	1.4528(19)
C2	C3	1.387(2)	C34	C35	1.447(2)

Atom	Atom	Length/Å
C35	C36	1.386(2)
C35	C51	1.446(2)
C36	C37	1.450(2)
C37	C38	1.446(2)
C37	C53	1.388(2)
C38	C39	1.391(2)
C39	C40	1.451(2)
C40	C41	1.451(2)
C40	C54	1.383(2)
C41	C42	1.3877(18)
C42	C43	1.4505(18)
C42	C56	1.4485(18)
C43	C44	1.4484(17)
C44	C45	1.3858(18)
C44	C57	1.4495(18)
C45	C46	1.4481(18)
C46	C47	1.3843(18)
C47	C48	1.4430(18)
C47	C58	1.4539(18)
C48	C49	1.4483(19)
C49	C50	1.3847(19)
C49	C59	1.4510(19)
C50	C51	1.450(2)

Atom	Atom	Length/Å
C51	C52	1.391(2)
C52	C53	1.442(2)
C52	C60	1.448(2)
C53	C54	1.452(2)
C54	C55	1.447(2)
C55	C56	1.3894(19)
C55	C60	1.449(2)
C56	C57	1.4467(18)
C57	C58	1.3873(18)
C58	C59	1.4432(18)
C59	C60	1.389(2)
C98	C99	1.3866(19)
C98	C103	1.385(2)
C99	C100	1.384(2)
C100	C101	1.383(2)
C101	C102	1.389(2)
C102	C103	1.386(2)
C104	C105	1.377(2)
C104	C109	1.380(2)
C105	C106	1.382(2)
C106	C107	1.383(2)
C107	C108	1.379(2)
C108	C109	1.383(2)

Table S3.2.4 Bond Angles (°) for Ne@C_{60}.

Atom	Atom	Atom	Angle/°
N1	Ni1	N3	179.23(4)
N1	Ni1	N4	89.80(4)
N2	Ni1	N1	90.11(4)
N2	Ni1	N3	89.76(4)
N2	Ni1	N4	179.30(4)
N4	Ni1	N3	90.32(4)
C62	N1	Ni1	127.89(7)
C62	N1	C65	104.15(9)
C65	N1	Ni1	127.96(7)
C67	N2	Ni1	127.54(7)
C67	N2	C70	104.47(9)
C70	N2	Ni1	127.98(7)
C72	N3	Ni1	128.27(7)
C75	N3	Ni1	127.63(7)
C75	N3	C72	104.10(9)
C77	N4	Ni1	127.56(7)
C77	N4	C80	104.35(9)
C80	N4	Ni1	128.06(7)
N1	C62	C63	111.60(10)
C81	C62	N1	124.85(10)
C81	C62	C63	123.41(10)
C62	C63	C82	125.46(10)
C64	C63	C62	106.23(10)
C64	C63	C82	128.31(10)
C63	C64	C65	106.46(10)
C63	C64	C84	128.09(10)
C65	C64	C84	125.44(10)
N1	C65	C64	111.48(10)
C66	C65	N1	124.27(10)
C66	C65	C64	124.04(10)
C67	C66	C65	123.87(10)
N2	C67	C66	125.06(10)
N2	C67	C68	111.16(10)
C66	C67	C68	123.68(10)
C67	C68	C86	124.78(10)

Atom	Atom	Atom	Angle/°
C69	C68	C67	106.55(9)
C69	C68	C86	128.62(10)
C68	C69	C70	106.37(9)
C68	C69	C88	128.26(10)
C70	C69	C88	125.33(10)
N2	C70	C69	111.37(9)
N2	C70	C71	124.80(10)
C71	C70	C69	123.65(10)
C72	C71	C70	123.86(10)
N3	C72	C73	111.52(9)
C71	C72	N3	124.37(10)
C71	C72	C73	124.11(10)
C72	C73	C90	126.10(10)
C74	C73	C72	106.29(9)
C74	C73	C90	127.61(10)
C73	C74	C75	106.26(9)
C73	C74	C92	128.67(10)
C75	C74	C92	125.00(10)
N3	C75	C74	111.82(9)
N3	C75	C76	124.84(10)
C76	C75	C74	123.24(10)
C77	C76	C75	123.75(10)
N4	C77	C76	125.08(10)
N4	C77	C78	111.47(9)
C76	C77	C78	123.32(10)
C77	C78	C94	124.71(10)
C79	C78	C77	106.42(9)
C79	C78	C94	128.80(10)
C78	C79	C80	106.37(9)
C78	C79	C96	128.12(10)
C80	C79	C96	125.51(10)
N4	C80	C79	111.38(9)
C81	C80	N4	124.62(10)
C81	C80	C79	123.98(10)
C80	C81	C62	123.77(10)

Atom	Atom	Atom	Angle/°
C63	C82	C83	114.33(10)
C64	C84	C85	112.99(10)
C68	C86	C87	112.46(9)
C69	C88	C89	113.18(10)
C73	C90	C91	113.30(10)
C74	C92	C93	113.48(9)
C78	C94	C95	113.03(9)
C79	C96	C97	113.15(10)
C6	C1	C2	120.02(14)
C6	C1	C10	120.00(13)
C10	C1	C2	107.99(12)
C3	C2	C1	119.84(13)
C3	C2	C12	120.10(12)
C12	C2	C1	108.14(12)
C2	C3	C4	120.11(13)
C2	C3	C15	119.95(12)
C15	C3	C4	107.93(12)
C3	C4	C17	108.12(12)
C5	C4	C3	120.04(14)
C5	C4	C17	119.63(14)
C4	C5	C6	119.86(13)
C4	C5	C20	119.98(14)
C6	C5	C20	108.39(13)
C1	C6	C5	120.12(13)
C1	C6	C7	120.20(14)
C5	C6	C7	107.59(13)
C8	C7	C6	119.67(14)
C8	C7	C21	120.03(13)
C21	C7	C6	108.10(13)
C7	C8	C9	120.08(13)
C7	C8	C24	119.88(14)
C24	C8	C9	107.95(12)
C10	C9	C8	120.11(13)
C10	C9	C26	119.87(12)
C26	C9	C8	108.03(12)
C1	C10	C11	107.94(12)
C9	C10	C1	119.94(13)
C9	C10	C11	119.98(13)
C12	C11	C10	108.18(12)
C28	C11	C10	120.05(12)
C28	C11	C12	119.88(12)
C2	C12	C11	107.76(12)
C13	C12	C2	119.94(12)
C13	C12	C11	120.09(12)
C12	C13	C14	120.12(12)
C12	C13	C30	119.95(12)
C14	C13	C30	107.95(11)
C13	C14	C32	107.93(11)
C15	C14	C13	119.94(12)
C15	C14	C32	120.05(12)
C14	C15	C3	119.95(12)
C14	C15	C16	120.01(12)
C16	C15	C3	108.00(12)
C15	C16	C17	108.19(12)
C34	C16	C15	119.83(12)
C34	C16	C17	119.88(13)
C16	C17	C4	107.77(12)
C18	C17	C4	120.22(13)
C18	C17	C16	119.84(14)
C17	C18	C19	120.10(15)
C17	C18	C36	120.12(13)
C36	C18	C19	108.05(13)
C18	C19	C38	107.91(14)
C20	C19	C18	119.86(14)
C20	C19	C38	120.38(14)
C19	C20	C5	120.20(13)
C19	C20	C21	119.98(14)
C21	C20	C5	107.70(14)
C20	C21	C7	108.21(13)
C22	C21	C7	120.11(13)
C22	C21	C20	119.69(15)
C21	C22	C23	119.75(14)
C21	C22	C39	120.50(14)
C39	C22	C23	108.03(12)
C24	C23	C22	120.12(13)
C24	C23	C41	120.07(12)
C41	C23	C22	107.93(13)
C23	C24	C8	120.11(13)
C23	C24	C25	119.84(12)
C25	C24	C8	108.02(12)
C24	C25	C26	108.00(12)
C43	C25	C24	120.28(12)
C43	C25	C26	120.01(12)
C9	C26	C25	107.99(12)
C27	C26	C9	120.23(12)
C27	C26	C25	119.71(12)
C26	C27	C28	119.68(12)
C26	C27	C45	120.13(12)
C45	C27	C28	108.13(11)
C11	C28	C27	120.19(12)
C11	C28	C29	120.12(12)
C29	C28	C27	107.91(11)
C28	C29	C46	107.97(11)
C30	C29	C28	120.10(12)
C30	C29	C46	120.20(12)
C29	C30	C13	119.87(12)
C29	C30	C31	119.79(11)
C31	C30	C13	108.16(11)
C30	C31	C32	107.91(11)
C48	C31	C30	119.92(12)
C48	C31	C32	119.89(12)
C14	C32	C31	108.05(11)
C33	C32	C14	119.99(12)
C33	C32	C31	119.91(12)
C32	C33	C34	120.15(12)
C32	C33	C50	120.17(12)
C34	C33	C50	107.91(12)
C16	C34	C33	119.96(12)
C16	C34	C35	120.21(13)
C35	C34	C33	107.99(12)
C36	C35	C34	119.86(13)
C36	C35	C51	120.14(13)
C51	C35	C34	108.20(12)
C18	C36	C37	107.96(14)
C35	C36	C18	120.08(13)
C35	C36	C37	119.98(14)
C38	C37	C36	108.03(14)
C53	C37	C36	119.77(14)
C53	C37	C38	120.30(14)
C37	C38	C19	108.05(13)
C39	C38	C19	119.59(15)
C39	C38	C37	119.95(14)
C22	C39	C40	108.22(13)
C38	C39	C22	119.85(14)
C38	C39	C40	119.77(14)
C39	C40	C41	107.71(13)

Atom	Atom	Atom	Angle/°
C54	C40	C39	120.17(13)
C54	C40	C41	119.98(12)
C23	C41	C40	108.12(12)
C42	C41	C23	120.03(12)
C42	C41	C40	119.82(13)
C41	C42	C43	120.01(12)
C41	C42	C56	120.07(12)
C56	C42	C43	107.96(11)
C25	C43	C42	119.76(12)
C25	C43	C44	120.20(12)
C44	C43	C42	107.98(11)
C43	C44	C57	107.96(11)
C45	C44	C43	119.81(12)
C45	C44	C57	120.01(11)
C27	C45	C46	107.89(11)
C44	C45	C27	120.14(11)
C44	C45	C46	120.10(12)
C45	C46	C29	108.10(11)
C47	C46	C29	119.84(11)
C47	C46	C45	120.05(12)
C46	C47	C48	119.94(11)
C46	C47	C58	119.95(11)
C48	C47	C58	107.96(11)
C31	C48	C47	120.30(12)
C31	C48	C49	120.14(12)
C47	C48	C49	108.03(11)
C48	C49	C59	108.06(11)
C50	C49	C48	119.93(12)
C50	C49	C59	120.01(13)
C49	C50	C33	119.95(12)
C49	C50	C51	119.97(13)
C51	C50	C33	107.87(12)
C35	C51	C50	108.04(12)
C52	C51	C35	119.83(14)
C52	C51	C50	119.97(13)
C51	C52	C53	120.04(14)
C51	C52	C60	120.12(13)
C53	C52	C60	108.00(13)
C37	C53	C52	120.24(14)
C37	C53	C54	119.67(14)
C52	C53	C54	108.32(13)
C40	C54	C53	120.13(13)
C40	C54	C55	120.34(13)
C55	C54	C53	107.59(13)
C54	C55	C60	108.17(12)
C56	C55	C54	119.77(13)
C56	C55	C60	119.74(12)
C55	C56	C42	120.02(12)
C55	C56	C57	119.91(12)
C57	C56	C42	108.05(11)
C56	C57	C44	108.06(11)
C58	C57	C44	119.95(11)
C58	C57	C56	120.28(12)
C57	C58	C47	119.93(11)
C57	C58	C59	119.87(12)
C59	C58	C47	108.08(11)
C58	C59	C49	107.87(11)
C60	C59	C49	120.17(12)
C60	C59	C58	120.05(12)
C52	C60	C55	107.92(13)
C59	C60	C52	119.76(13)
C59	C60	C55	120.15(12)
C103	C98	C99	119.65(14)

Atom	Atom	Atom	Angle/°
C100	C99	C98	120.27(13)
C101	C100	C99	120.04(13)
C100	C101	C102	119.87(14)
C103	C102	C101	119.95(13)
C98	C103	C102	120.17(13)
C105	C104	C109	120.40(13)
C104	C105	C106	119.74(14)
C105	C106	C107	120.01(14)
C108	C107	C106	120.16(14)
C107	C108	C109	119.76(14)
C104	C109	C108	119.93(14)

Table S3.2.5 Hydrogen Fractional Atomic Coordinates (×10[4]) and Equivalent Isotropic Displacement Parameters (Å[2]×10[3]) for Ne@C_{60}. U_{eq} is defined as 1/3 of the trace of the orthogonalised U_{ij}.

Atom	x	y	z	U_{eq}
H66	1856.22	6430.71	8123.34	16
H71	3778.65	8679	10019.86	14
H76	7124.18	4991.75	10037.87	14
H81	5913.6	3388.17	7258.39	16
H82A	5120.6	3354.67	6268.58	21
H82B	4025.71	3630.34	6088.83	21
H83A	4443.62	2000.34	6402.81	40
H83B	3495.58	2548.92	7083.21	40
H83C	4598.83	2266.08	7250.66	40
H84A	2434.7	4623.79	6502.5	22
H84B	1909.37	5630.95	6967.91	22
H85A	872.41	4548.79	7354.91	46
H85B	1222.99	4822.64	8114.82	46
H85C	1757.53	3811.59	7656.83	46
H86A	941.74	8003.36	8217.06	20
H86B	664.48	8935	8784.49	20
H87A	-528.75	8013.68	9243.45	33
H87B	82.08	8028.79	9912.68	33
H87C	351.76	7101.66	9341.3	33
H88A	1489.47	9827.73	9344.78	20
H88B	2523.11	9771.43	9594.36	20
H89A	824.03	9212.38	10604.94	39
H89B	1165.52	10164.98	10733.59	39
H89C	1862.82	9132.12	10854.64	39
H90A	5295.49	8394.32	11455.39	18
H90B	4504.1	8905.66	10948.34	18
H91A	5832.71	9293.17	9951.8	47
H91B	6618.95	8790.25	10466.73	47
H91C	5767.52	9742.79	10805.2	47
H92A	7056.38	5954.05	11102.18	17
H92B	6773.11	7070.41	11338.56	17
H93A	8172.69	6243.6	9883.5	29
H93B	8489.16	6582.01	10637.87	29
H93C	7890.54	7360.27	10121.97	29
H94A	8563.21	2697.41	9027.96	17
H94B	8086.33	3436.61	9773.18	17
H95A	9295.98	3876.78	8268.86	28
H95B	9678.34	3666.63	9077.33	28
H95C	8820.55	4614.77	9015.63	28
H96A	7154.56	2267.71	7536.47	21
H96B	7964.59	2015.87	8070.88	21
H97A	8764.27	2129.66	6694.33	40
H97B	8972.47	2913.03	7222.75	40
H97C	8147	3228.06	6705.07	40
H98	3087.19	5987.4	4205.01	30
H99	2262.8	7613.18	4424.85	30
H100	2305.85	8419.7	5560.05	33
H101	3199.69	7612.69	6467.4	35
H102	4104.4	6009.94	6210.25	37
H103	4016.76	5189.9	5095.7	35
H104	2842.5	9334.05	7089.4	36
H105	1166.76	9481.78	7071.62	31
H106	-61.94	10855.19	7621.91	38
H107	391.47	12074.68	8187.9	44
H108	2070.05	11923.91	8207.02	39
H109	3299.76	10552.93	7648.37	38

S4. References for the supporting information

[1] S. Bloodworth, G. Sitinova, S. Alom, S. Vidal, G. R. Bacanu, S. J. Elliott, M. E. Light, J. M. Herniman, G. J. Langley, M. H. Levitt and R. J. Whitby, *Angew. Chem. Int. Ed.* **2019**, *58*, 5038-5043.

[2] K. Kurotobi and Y. Murata, *Science*, **2011**, *333*, 613-616.

[3] A. Krachmalnicoff, R. Bounds, S. Mamone, S. Alom, M. Concistrè, B. Meier, K. Kouřil, M. E. Light, M. R. Johnson, S. Rols, A. J. Horsewill, A. Shugai, U. Nagel, T. Rõõm, M. Carravetta, M. H. Levitt and R. J. Whitby, *Nat. Chem.*, **2016**, *8*, 953-957.

[4] A. Krachmalnicoff, M. H. Levitt and R. J. Whitby, *Chem. Commun.* **2014**, *50*, 13037-13040.

[5] R. K. Harris, E. D. Becker, S. M. Cabral de Menezes, R. Goodfellow and P. Granger, *Solid State Nucl. Magn. Reson.*, **2002**, *22*, 458-483.

[6] M. Murata, Y. Murata and K. Komatsu, *J. Am. Chem. Soc.*, **2006**, *128*, 8024-8033.

[7] (a) M. Saunders, R. J. Cross, H. A. Jiménez-Vázquez, R. Shimshi and A. Khong, *Science*, **1996**, *271*, 1693-1697. (b) M. Saunders, H. A. Jiménez-Vázquez, R. J. Cross, S. Mroczkowski, D. I. Freedberg and F. A. L. Anet, *Nature*, **1994**, *367*, 256-258.

[8] Gaussian 09, Revision D.01, M. J. Frisch, G. W. Trucks, H. B. Schlegel, G. E. Scuseria, M. A. Robb, J. R. Cheeseman, G. Scalmani, V. Barone, B. Mennucci, G. A. Petersson, H. Nakatsuji, M. Caricato, X. Li, H. P. Hratchian, A. F. Izmaylov, J. Bloino, G. Zheng, J. L. Sonnenberg, M. Hada, M. Ehara, K. Toyota, R. Fukuda, J. Hasegawa, M. Ishida, T. Nakajima, Y. Honda, O. Kitao, H. Nakai, T. Vreven, J. A. Montgomery, Jr., J. E. Peralta, F. Ogliaro, M. Bearpark, J. J. Heyd, E. Brothers, K. N. Kudin, V. N. Staroverov, T. Keith, R. Kobayashi, J. Normand, K. Raghavachari, A. Rendell, J. C. Burant, S. S. Iyengar, J. Tomasi, M. Cossi, N. Rega, J. M. Millam, M. Klene, J. E. Knox, J. B. Cross, V. Bakken, C. Adamo, J. Jaramillo, R. Gomperts, R. E. Stratmann, O. Yazyev, A. J. Austin, R. Cammi, C. Pomelli, J. W. Ochterski, R. L. Martin, K. Morokuma, V. G. Zakrzewski, G. A. Voth, P. Salvador, J. J. Dannenberg, S. Dapprich, A. D. Daniels, O. Farkas, J. B. Foresman, J. V. Ortiz, J. Cioslowski, and D. J. Fox, Gaussian, Inc., Wallingford CT, **2013**.

[9] Y. Zhao and D. G. Truhlar, *Theor. Chem. Acc.*, **2008**, *120*, 215-241.

[10] T. H. Dunning Jr., *J. Chem. Phys.*, **1989**, *90*, 1007-1023.

[11] S. F. Boys and F. Bernardi, *Mol. Phys.*, **1970**, *19*, 553-566.

[12] J. W. Ochterski, Thermochemistry in Gaussian, 2000. https://gaussian.com/thermo/

[13] (a) Sheldrick, G.M, *Acta Cryst.*, **2015**, *A71*, 3-8. (b) Sheldrick, G.M, *Acta Cryst.*, **2015**, *C27*, 3-8.

[14] O.V. Dolomanov, L.J. Bourhis, R.J. Gildea, J.A.K. Howard and H. Puschmann, *J. Appl. Cryst.*, **2009**, *42*, 339-341.

[15] M. M. Olmstead, D. A. costa, K. Maitra, B. C. Noll, S. L. Phillips, P. M. van Calcar and A. L. Balch, *J. Am. Chem. Soc.*, **1999**, *121*, 7090-7097.

S5. Author contributions

The project was conceived and coordinated by R.J.W and M.H.L. The manuscript was written by R.J.W and S.B. The pressure intensifier apparatus for filling experiments was designed and built by R.J.W. Synthesis and purification of all compounds was carried out by G.H, M.C.W, J.G and S.A, verification of some procedures was conducted by S.B. NMR analysis of $Ne@C_{60}$, $^{3}He@C_{60}$ and $^{4}He@C_{60}$ was carried out by G.R.B. Crystal structures were acquired and solved by M.E.L.
